# Supplementary material for: Substituent-Controllable Cascade Regioselective Annulation of β-Enaminones with N-Sulfonyl Triazoles for Modular Access to Imidazoles and Pyrroles
Source: Molecules. 2023 May 29;28(11):4416. doi: 10.3390/molecules28114416 (PMC10254510; doi:10.3390/molecules28114416)

Supporting Information

Substituent-controllable regioselective annulation of *β*-enaminones with *N*-sulfonyl triazoles for modular access to imidazoles and pyrroles

Hua Wang,[1] Tongtong Zhou,[1] Mengdi Wu,[1] Qingqing Ye,[2,*] and Xinwei He,[1,*]

[1] Key Laboratory of Functional Molecular Solids, Ministry of Education, Anhui Laboratory of Molecule-Based Materials (State Key Laboratory Cultivation Base), College of Chemistry and Materials Science, Anhui Normal University, Wuhu 241000, China

[2] Department of Medicine, Chuzhou City Vocation College, Chuzhou 239000, China

*Corresponding authors: yeqingqing1983@sohu.com; xinweihe@mail.ahnu.edu.cn

Table of contents

1. X-ray crystallographic data of compound **3a**--S2

2. X-ray crystallographic data of compound **5a**--S2

3. NMR spectra for all compounds--S3

4. GC-MS spectra for mechanistic investigations--S56

1. X-ray crystallographic data of compound 3a

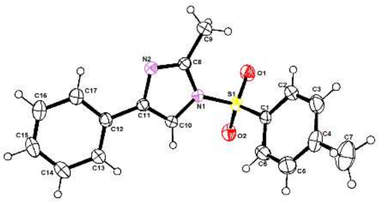

Figure S1. ORTEP drawing of compound **3a** (30% probability for the thermal ellipsoid).

The purified compound **3a** is dissolved in a mixed solvent of ethyl acetate and petroleum ether, and placed in a dark cabinet to slowly evaporate. After several days, a colourless bulk crystal was obtained. The X-ray crystal-structure determinations were obtained on a Bruker Smart CCDC APEX-2 diffractometer (graphite- monochromated Mo *Kα* radiation, λ=0.71073 nm) at 298 K.

Table S1. Crystal data and structure refinement for compound **3a**.

CCDC number	2260879	
Identification code	20191014c	
Empirical formula	C17 H16 N2 O2 S	
Formula weight	312.38	
Temperature	298.15 K	
Wavelength	0.71073 Å	
Crystal system	Monoclinic	
Space group	$P2_1/c$	
Unit cell dimensions	a = 12.6175(5) Å	α = 90°.
	b = 12.5587(5) Å	β = 99.2860(10).
	c = 10.4154(4) Å	γ = 90°.
Volume	1628.79(11) Å^3	
Z	4	
Density (calculated)	1.274 g/cm^3	
μ	0.207 mm^{-1}	
F(000)	656.0	
Crystal size	0.21 × 0.2 × 0.19 mm^3	
2Θ range for data collection	5.722 to 55.072°	
Index ranges	-16 ≤ h ≤ 16, -16 ≤ k ≤ 16, -13 ≤ l ≤ 13	
Reflections collected	42190	
Independent reflections	3738 [R(int) = 0.0258, R(sigma) = 0.0119]	
Data / restraints / parameters	3738 / 0 / 201	
Goodness-of-fit on F^2	1.088	
Final R indices [I>2sigma(I)]	R_1 = 0.0429, wR_2 = 0.1112	
Final R indices (all data)	R_1 = 0.0511, wR_2 = 0.1204	
Largest diff. peak and hole	0.21 and -0.43 eÅ^{-3}	

1. X-ray crystallographic data of compound 5a

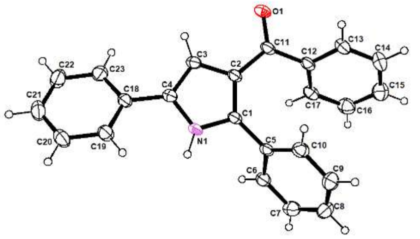

Figure S2. ORTEP drawing of compound **5a** (30% probability for the thermal ellipsoid).

The purified compound **5a** is dissolved in a mixed solvent of ethyl acetate and petroleum ether, and placed in a dark cabinet to slowly evaporate. After several days, a colourless bulk crystal was obtained. The X-ray crystal-structure determinations were obtained on a Bruker Smart CCDC APEX-2 diffractometer (graphite- monochromated Mo *Kα* radiation, λ=0.71073 nm) at 298 K.

Table S2. Crystal data and structure refinement for compound **5a**.

CCDC number	2260880	
Identification code	mo_20200109b_0m_a	
Empirical formula	C23 H17 N O	
Formula weight	323.38	
Temperature	298.15 K	
Wavelength	0.71073 Å	
Crystal system	Triclinic	
Space group	P-1	
Unit cell dimensions	a = 9.9209(10) Å	α = 113.914(4)°.
	b = 13.3600(16) Å	β = 91.364(4)°.
	c = 14.6495(16) Å	γ = 102.145(5)°.
Volume	1722.3(3) $Å^3$	
Z	4	
Density (calculated)	1.247 g/cm^3	
μ	0.076 mm^{-1}	
F(000)	680.0	
Crystal size	0.23 × 0.21 × 0.2 mm^3	
2Θ range for data collection	5.84 to 55.168°	
Index ranges	$-12 \leq h \leq 12$, $-17 \leq k \leq 17$, $-19 \leq l \leq 19$	
Reflections collected	68223	
Independent reflections	7947 [R(int) = 0.0439, R(sigma) = 0.0241]	
Data / restraints / parameters	7947 / 0 / 451	
Goodness-of-fit on F^2	1.046	
Final R indices [I>2sigma(I)]	R_1 = 0.0547, wR_2 = 0.1552	
Final R indices (all data)	R_1 = 0.0784, wR_2 = 0.1779	
Largest diff. peak and hole	0.26 and -0.29 $eÅ^{-3}$	

3. NMR spectra for all compounds

2-Methyl-4-phenyl-1-tosyl-1*H*-imidazole (3a)

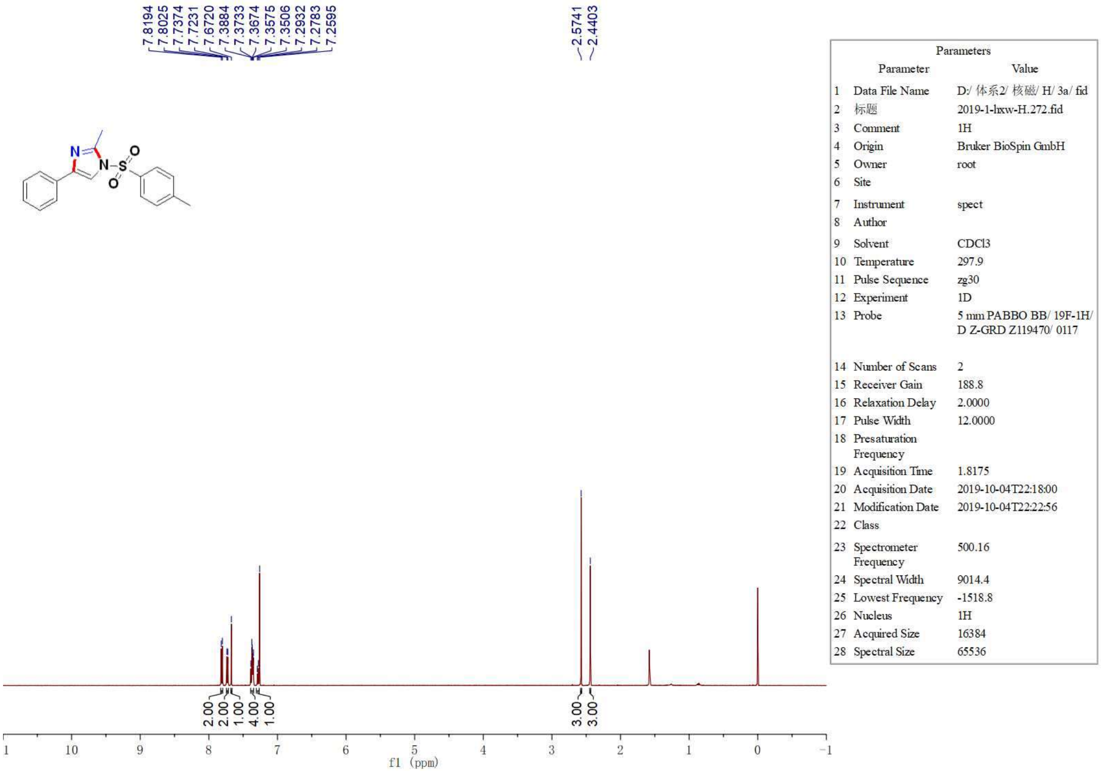

Parameter	Value
1 Data File Name	D:/ 体系2/ 核磁/ H/ 3a/ fid
2 标题	2019-1-hxw-H.272.fid
3 Comment	1H
4 Origin	Bruker BioSpin GmbH
5 Owner	root
6 Site	
7 Instrument	spect
8 Author	
9 Solvent	CDCl3
10 Temperature	297.9
11 Pulse Sequence	zg30
12 Experiment	1D
13 Probe	5 mm PABBO BB/ 19F-1H/ D Z-GRD Z119470/ 0117
14 Number of Scans	2
15 Receiver Gain	188.8
16 Relaxation Delay	2.0000
17 Pulse Width	12.0000
18 Presaturation Frequency	
19 Acquisition Time	1.8175
20 Acquisition Date	2019-10-04T22:18:00
21 Modification Date	2019-10-04T22:22:56
22 Class	
23 Spectrometer Frequency	500.16
24 Spectral Width	9014.4
25 Lowest Frequency	-1518.8
26 Nucleus	1H
27 Acquired Size	16384
28 Spectral Size	65536

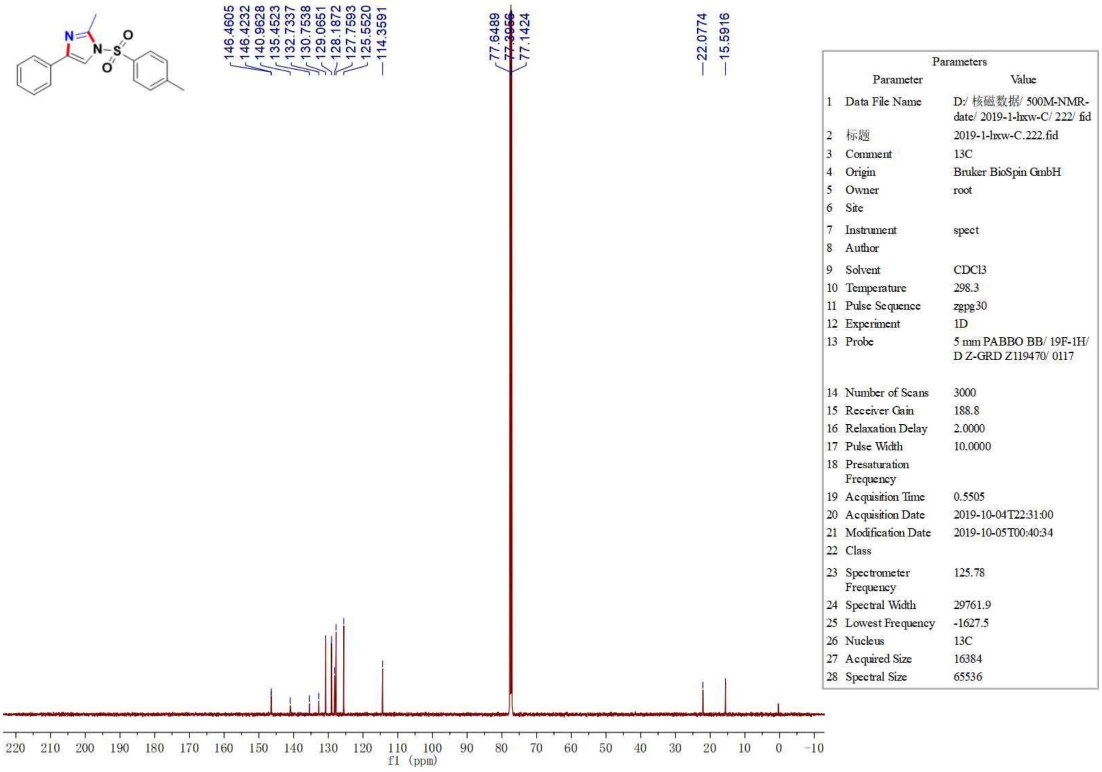

Parameter	Value
1 Data File Name	D:/ 核磁数据/ 500M-NMR-date/ 2019-1-hxw-C/ 222/ fid
2 标题	2019-1-hxw-C.222.fid
3 Comment	13C
4 Origin	Bruker BioSpin GmbH
5 Owner	root
6 Site	
7 Instrument	spect
8 Author	
9 Solvent	CDCl3
10 Temperature	298.3
11 Pulse Sequence	zgpg30
12 Experiment	1D
13 Probe	5 mm PABBO BB/ 19F-1H/ D Z-GRD Z119470/ 0117
14 Number of Scans	3000
15 Receiver Gain	188.8
16 Relaxation Delay	2.0000
17 Pulse Width	10.0000
18 Presaturation Frequency	
19 Acquisition Time	0.5505
20 Acquisition Date	2019-10-04T22:31:00
21 Modification Date	2019-10-05T00:40:34
22 Class	
23 Spectrometer Frequency	125.78
24 Spectral Width	29761.9
25 Lowest Frequency	-1627.5
26 Nucleus	13C
27 Acquired Size	16384
28 Spectral Size	65536

2-Methyl-4-(*p*-tolyl)-1-tosyl-1*H*-imidazole (3b)

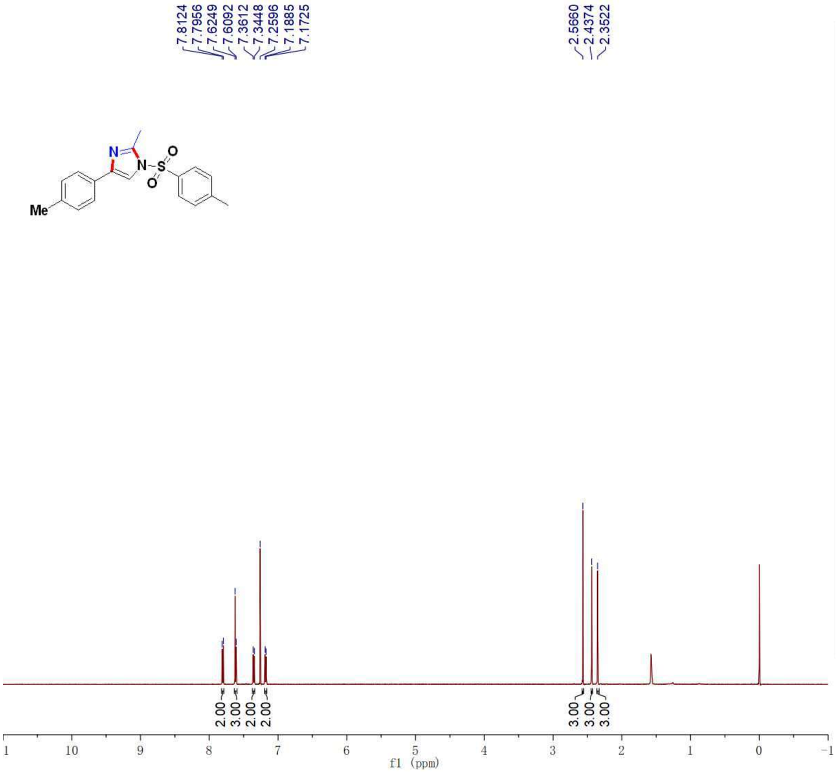

Parameter		Value
1	Data File Name	D:/ 体系2/ 核磁/ H/ 4-甲基/ fid
2	标题	2019-1-hxw-H.275.fid
3	Comment	1H
4	Origin	Bruker BioSpin GmbH
5	Owner	root
6	Site	
7	Instrument	spect
8	Author	
9	Solvent	CDCl3
10	Temperature	297.7
11	Pulse Sequence	zg30
12	Experiment	1D
13	Probe	5 mm PABBO BB/ 19F-1H/ D Z-GRD Z119470/ 0117
14	Number of Scans	2
15	Receiver Gain	150.9
16	Relaxation Delay	2.0000
17	Pulse Width	12.0000
18	Presaturation Frequency	
19	Acquisition Time	1.8175
20	Acquisition Date	2019-10-10T11:17:00
21	Modification Date	2019-10-10T11:17:38
22	Class	
23	Spectrometer Frequency	500.16
24	Spectral Width	9014.4
25	Lowest Frequency	-1518.8
26	Nucleus	1H
27	Acquired Size	16384
28	Spectral Size	65536

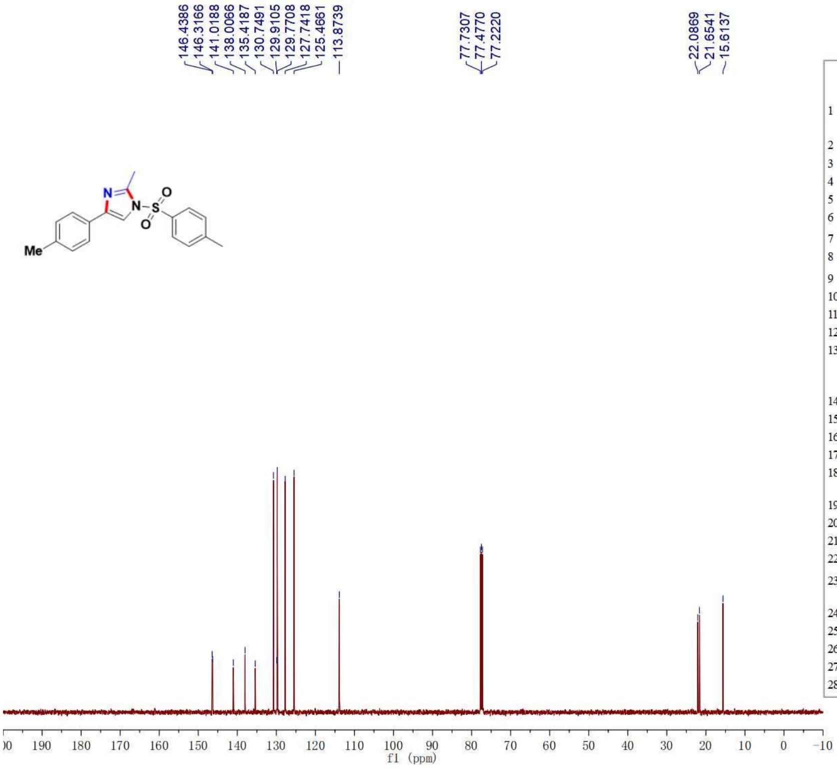

Parameter		Value
1	Data File Name	D:/ 核磁数据/ 500M-NMR-date/ 2019-1-hxw-C/ 227/ fid
2	标题	2019-1-hxw-C.227.fid
3	Comment	13C
4	Origin	Bruker BioSpin GmbH
5	Owner	root
6	Site	
7	Instrument	spect
8	Author	
9	Solvent	CDCl3
10	Temperature	298.2
11	Pulse Sequence	zgpg30
12	Experiment	1D
13	Probe	5 mm PABBO BB/ 19F-1H/ D Z-GRD Z119470/ 0117
14	Number of Scans	40
15	Receiver Gain	188.8
16	Relaxation Delay	2.0000
17	Pulse Width	10.0000
18	Presaturation Frequency	
19	Acquisition Time	0.5505
20	Acquisition Date	2019-10-10T22:36:00
21	Modification Date	2019-10-10T22:37:28
22	Class	
23	Spectrometer Frequency	125.78
24	Spectral Width	29761.9
25	Lowest Frequency	-1627.5
26	Nucleus	13C
27	Acquired Size	16384
28	Spectral Size	65536

4-(4-Ethylphenyl)-2-methyl-1-tosyl-1*H*-imidazole (3c)

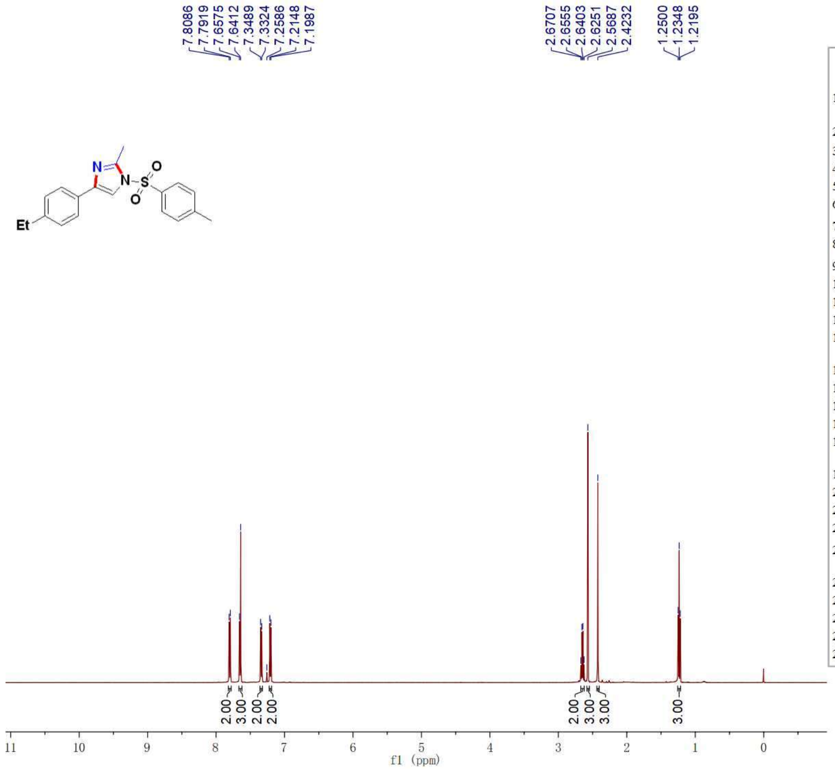

Parameters		
	Parameter	Value
1	Data File Name	D:/ 体系2/ 核磁/ H/ 4-乙基/ fid
2	标题	2020-1-syj-H.16.fid
3	Comment	1H ztt
4	Origin	Bruker BioSpin GmbH
5	Owner	root
6	Site	
7	Instrument	spect
8	Author	
9	Solvent	CDCl3
10	Temperature	294.6
11	Pulse Sequence	zg30
12	Experiment	1D
13	Probe	Z119470_0117 (PA BBO 500S1 BBF-H-D-05 Z SP)
14	Number of Scans	2
15	Receiver Gain	30.7
16	Relaxation Delay	2.0000
17	Pulse Width	12.0000
18	Presaturation Frequency	
19	Acquisition Time	1.8175
20	Acquisition Date	2019-12-30T11:00:05
21	Modification Date	2019-12-30T11:00:06
22	Class	
23	Spectrometer Frequency	500.16
24	Spectral Width	9014.4
25	Lowest Frequency	-1519.4
26	Nucleus	1H
27	Acquired Size	16384
28	Spectral Size	65536

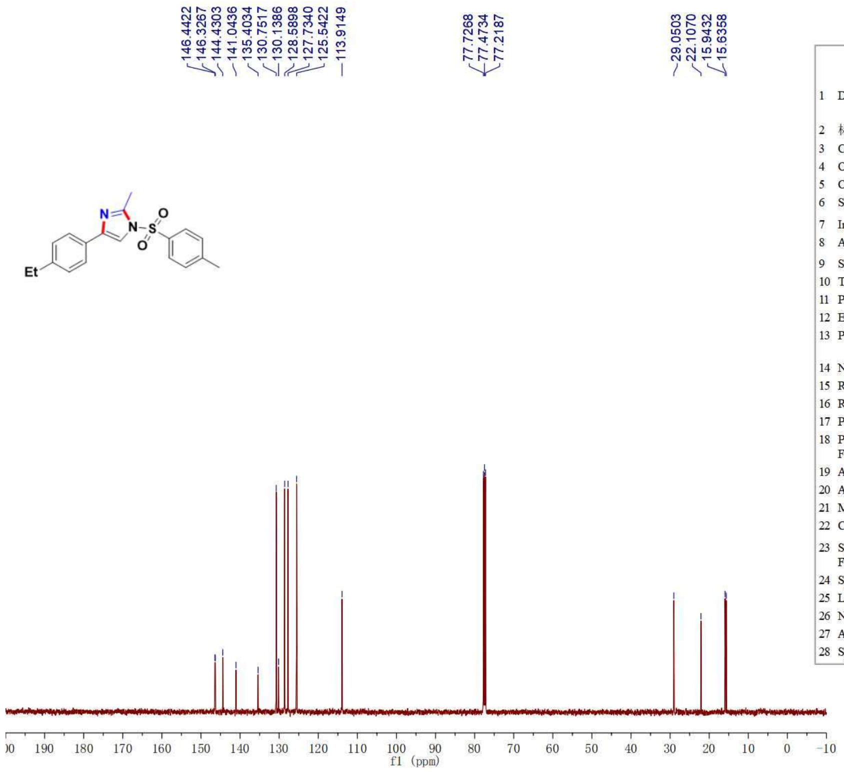

Parameters		
	Parameter	Value
1	Data File Name	D:/ 核磁数据/ nmr/ nmr/ 2020-1-syj-C/ 1/ fid
2	标题	2020-1-syj-C.1.fid
3	Comment	13C
4	Origin	Bruker BioSpin GmbH
5	Owner	root
6	Site	
7	Instrument	spect
8	Author	
9	Solvent	CDCl3
10	Temperature	295.3
11	Pulse Sequence	zgpg30
12	Experiment	1D
13	Probe	Z119470_0117 (PA BBO 500S1 BBF-H-D-05 Z SP)
14	Number of Scans	55
15	Receiver Gain	188.8
16	Relaxation Delay	2.0000
17	Pulse Width	10.0000
18	Presaturation Frequency	
19	Acquisition Time	0.5505
20	Acquisition Date	2019-12-30T11:31:59
21	Modification Date	2019-12-30T11:32:02
22	Class	
23	Spectrometer Frequency	125.78
24	Spectral Width	29761.9
25	Lowest Frequency	-1627.5
26	Nucleus	13C
27	Acquired Size	16384
28	Spectral Size	65536

4-(4-Methoxyphenyl)-2-methyl-1-tosyl-1*H*-imidazole (3d)

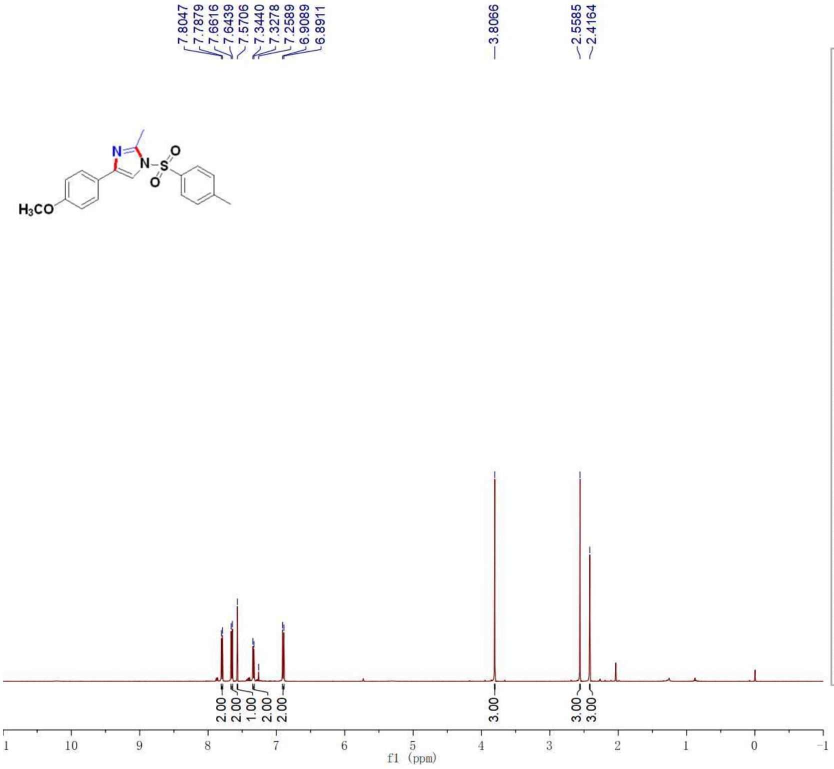

	Parameters	
	Parameter	Value
1	Data File Name	D:/ 体系2/ 核磁/ H/ 4-甲氧基/ fid
2	标题	2019-1-lxw-H.340.fid
3	Comment	1H
4	Origin	Bruker BioSpin GmbH
5	Owner	root
6	Site	
7	Instrument	spect
8	Author	
9	Solvent	CDCl3
10	Temperature	295.9
11	Pulse Sequence	zg30
12	Experiment	1D
13	Probe	5 mm PABBO BB/ 19F-1H/ D Z-GRD Z119470/ 0117
14	Number of Scans	2
15	Receiver Gain	30.7
16	Relaxation Delay	2.0000
17	Pulse Width	12.0000
18	Presaturation Frequency	
19	Acquisition Time	1.8175
20	Acquisition Date	2019-12-10T12:42:00
21	Modification Date	2019-12-10T12:42:30
22	Class	
23	Spectrometer Frequency	500.16
24	Spectral Width	9014.4
25	Lowest Frequency	-1518.8
26	Nucleus	1H
27	Acquired Size	16384
28	Spectral Size	65536

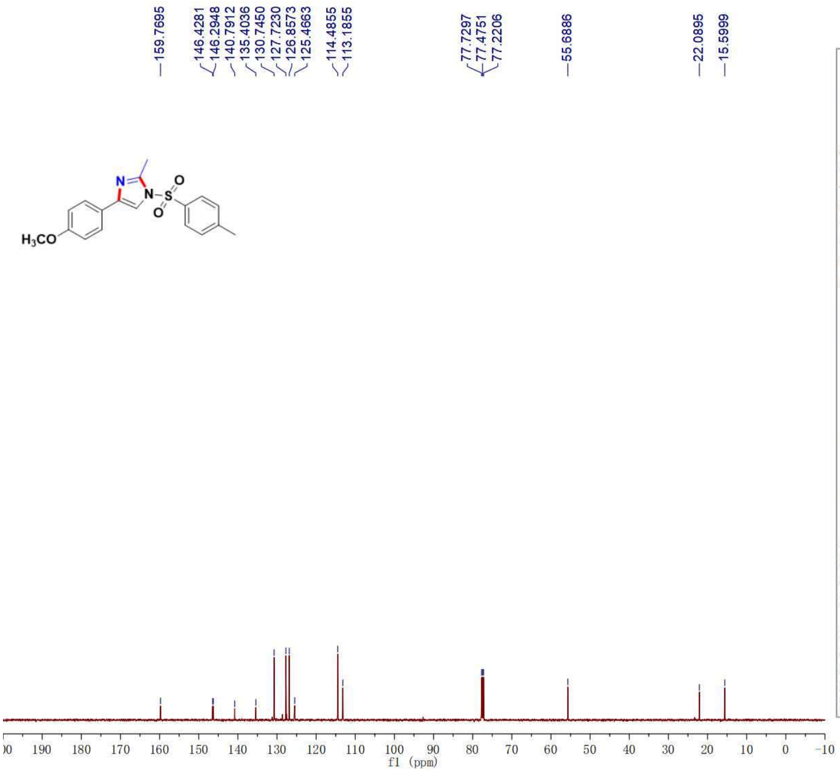

	Parameters	
	Parameter	Value
1	Data File Name	D:/ 核磁数据/ 500M-NMR-date/ 2019-1-lxw-C/ 262/ fid
2	标题	2019-1-lxw-C.262.fid
3	Comment	13C
4	Origin	Bruker BioSpin GmbH
5	Owner	root
6	Site	
7	Instrument	spect
8	Author	
9	Solvent	CDCl3
10	Temperature	296.3
11	Pulse Sequence	zgpg30
12	Experiment	1D
13	Probe	5 mm PABBO BB/ 19F-1H/ D Z-GRD Z119470/ 0117
14	Number of Scans	30
15	Receiver Gain	188.8
16	Relaxation Delay	2.0000
17	Pulse Width	10.0000
18	Presaturation Frequency	
19	Acquisition Time	0.5505
20	Acquisition Date	2019-12-11T11:27:00
21	Modification Date	2019-12-11T11:28:54
22	Class	
23	Spectrometer Frequency	125.78
24	Spectral Width	29761.9
25	Lowest Frequency	-1627.5
26	Nucleus	13C
27	Acquired Size	16384
28	Spectral Size	65536

4-(4-Fluorophenyl)-2-methyl-1-tosyl-1*H*-imidazole (3e)

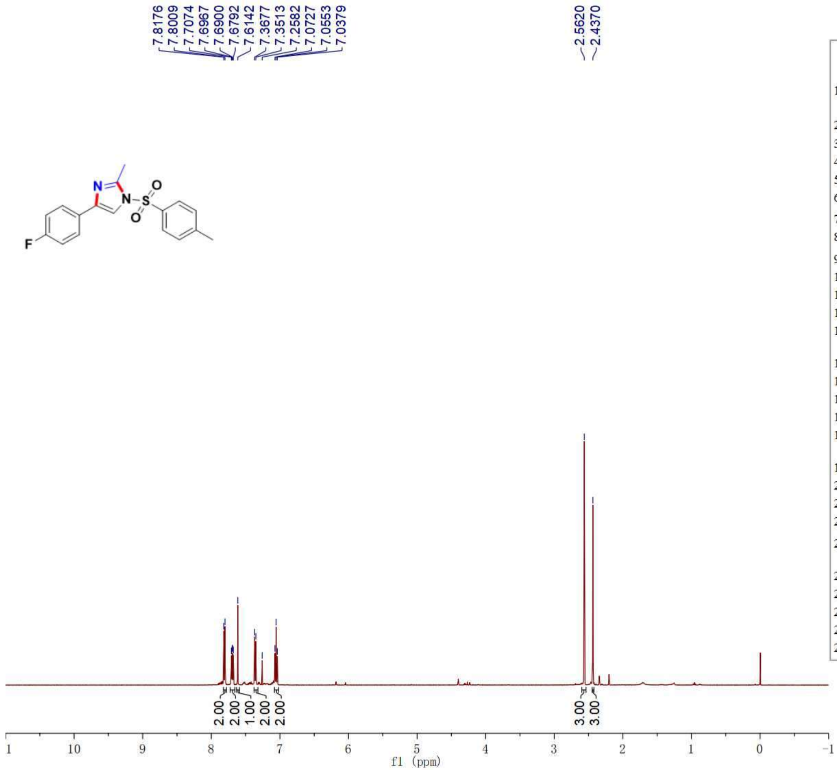

Parameters		
	Parameter	Value
1	Data File Name	D:/ 核磁数据/ 2020-1-lxw-H/ 15/ fid
2	标题	2020-1-lxw-H.15.fid
3	Comment	1H
4	Origin	Bruker BioSpin GmbH
5	Owner	root
6	Site	
7	Instrument	spect
8	Author	
9	Solvent	CDCl3
10	Temperature	295.7
11	Pulse Sequence	zg30
12	Experiment	1D
13	Probe	Z119470_0117 (PA BBO 500S1 BBF-H-D-05 Z SP)
14	Number of Scans	2
15	Receiver Gain	87.0
16	Relaxation Delay	2.0000
17	Pulse Width	12.0000
18	Presaturation Frequency	
19	Acquisition Time	1.8175
20	Acquisition Date	2019-12-26T11:01:59
21	Modification Date	2019-12-26T11:02:00
22	Class	
23	Spectrometer Frequency	500.16
24	Spectral Width	9014.4
25	Lowest Frequency	-1519.4
26	Nucleus	1H
27	Acquired Size	16384
28	Spectral Size	65536

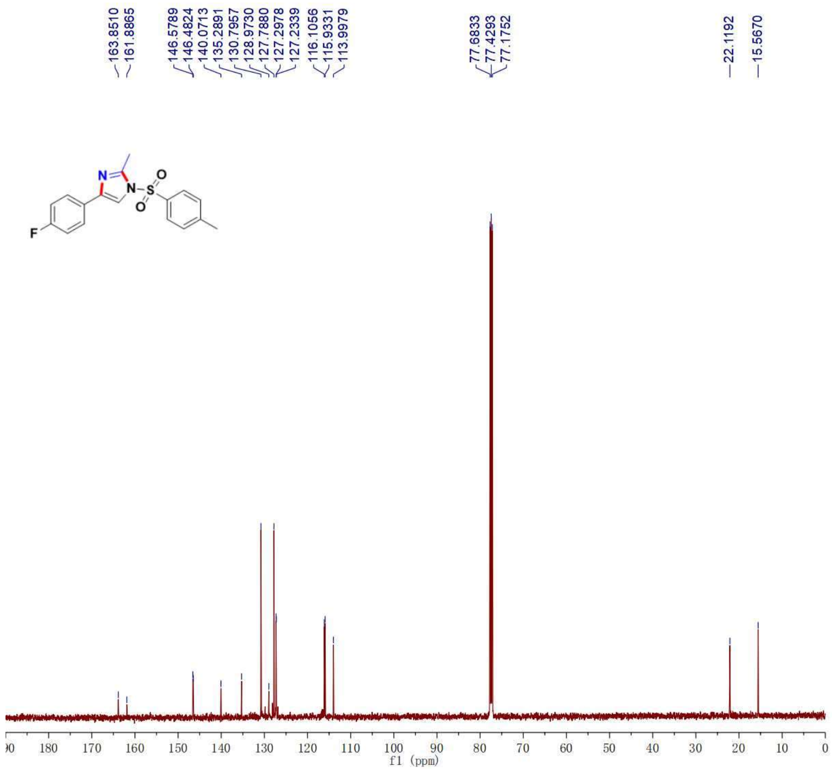

Parameters		
	Parameter	Value
1	Data File Name	D:/ 核磁数据/ 2020-1-lxw-C/ 15/ fid
2	标题	2020-1-lxw-C.15.fid
3	Comment	13C
4	Origin	Bruker BioSpin GmbH
5	Owner	root
6	Site	
7	Instrument	spect
8	Author	
9	Solvent	CDCl3
10	Temperature	296.0
11	Pulse Sequence	zgpg30
12	Experiment	1D
13	Probe	Z119470_0117 (PA BBO 500S1 BBF-H-D-05 Z SP)
14	Number of Scans	180
15	Receiver Gain	188.8
16	Relaxation Delay	2.0000
17	Pulse Width	10.0000
18	Presaturation Frequency	
19	Acquisition Time	0.5505
20	Acquisition Date	2019-12-26T11:12:06
21	Modification Date	2019-12-26T11:12:08
22	Class	
23	Spectrometer Frequency	125.78
24	Spectral Width	29761.9
25	Lowest Frequency	-1627.5
26	Nucleus	13C
27	Acquired Size	16384
28	Spectral Size	65536

4-(4-Chlorophenyl)-2-methyl-1-tosyl-1*H*-imidazole (3f)

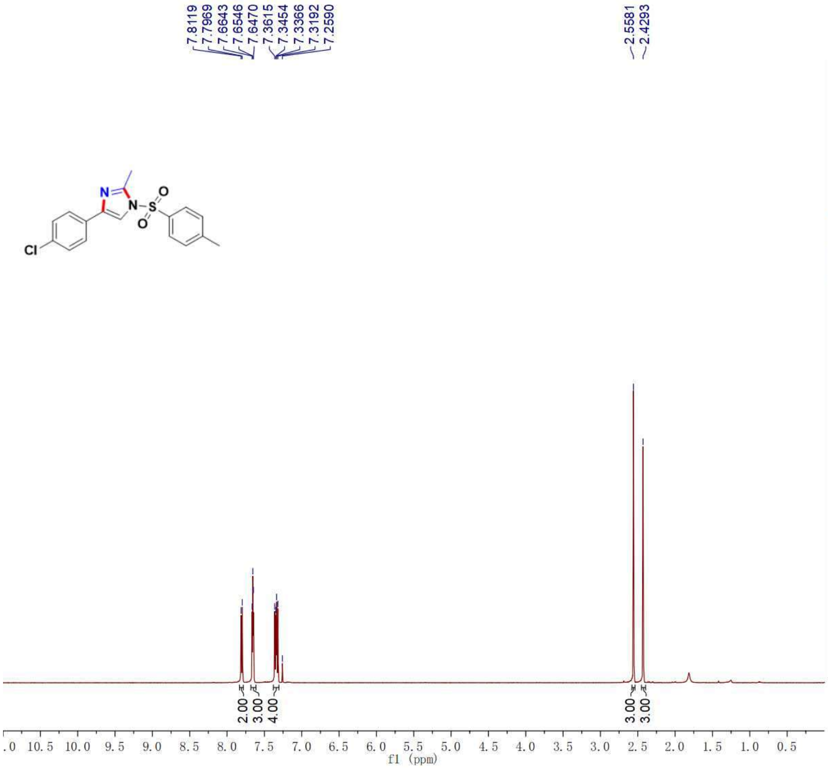

Parameters	Parameter	Value
1	Data File Name	D:/ 核磁数据/ 20201011/ 2020-1-syj-H/ 274/ fid
2	标题	2020-1-syj-H.274.fid
3	Comment	1H ztt
4	Origin	Bruker BioSpin GmbH
5	Owner	nmrsu
6	Site	
7	Instrument	spect
8	Author	
9	Solvent	CDCl3
10	Temperature	299.0
11	Pulse Sequence	zg30
12	Experiment	1D
13	Probe	Z119470_0117 (PA BBO 500S1 BBF-H-D-05 Z SP)
14	Number of Scans	2
15	Receiver Gain	66.5
16	Relaxation Delay	2.0000
17	Pulse Width	12.0000
18	Presaturation Frequency	
19	Acquisition Time	1.8175
20	Acquisition Date	2020-08-01T10:45:24
21	Modification Date	2020-08-01T10:45:26
22	Class	
23	Spectrometer Frequency	500.16
24	Spectral Width	9014.4
25	Lowest Frequency	-1519.4
26	Nucleus	1H
27	Acquired Size	16384
28	Spectral Size	65536

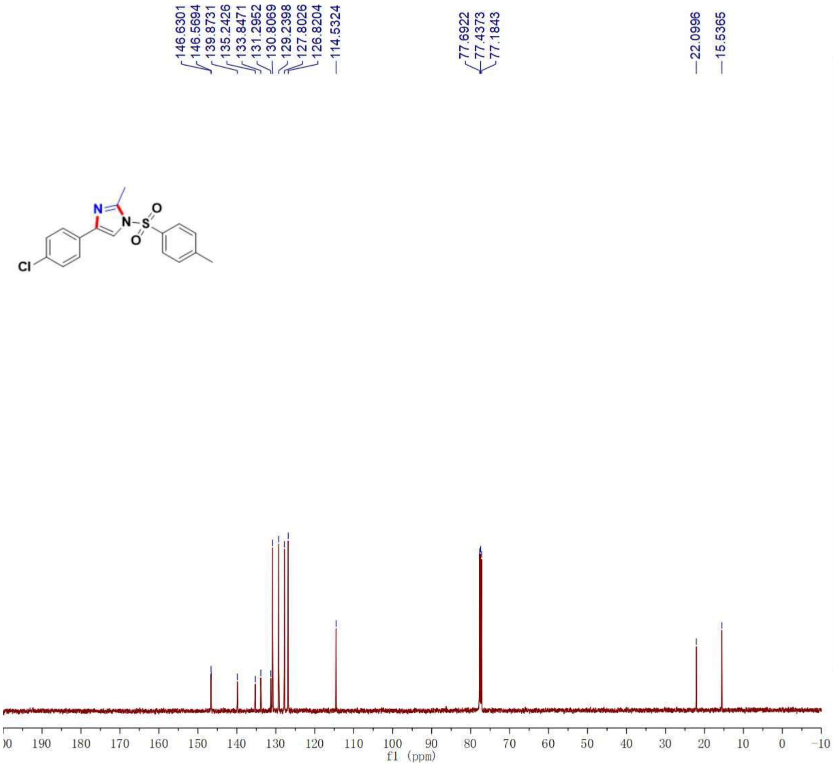

Parameters	Parameter	Value
1	Data File Name	D:/ 核磁数据/ 20201011/ 2020-1-syj-C/ 117/ fid
2	标题	2020-1-syj-C.117.fid
3	Comment	13C ztt
4	Origin	Bruker BioSpin GmbH
5	Owner	nmrsu
6	Site	
7	Instrument	spect
8	Author	
9	Solvent	CDCl3
10	Temperature	300.7
11	Pulse Sequence	zgpg30
12	Experiment	1D
13	Probe	Z119470_0117 (PA BBO 500S1 BBF-H-D-05 Z SP)
14	Number of Scans	90
15	Receiver Gain	188.8
16	Relaxation Delay	2.0000
17	Pulse Width	10.0000
18	Presaturation Frequency	
19	Acquisition Time	0.5505
20	Acquisition Date	2020-08-01T16:31:49
21	Modification Date	2020-08-01T16:31:52
22	Class	
23	Spectrometer Frequency	125.78
24	Spectral Width	29761.9
25	Lowest Frequency	-1627.5
26	Nucleus	13C
27	Acquired Size	16384
28	Spectral Size	65536

4-(4-Bromophenyl)-2-methyl-1-tosyl-1*H*-imidazole (3g)

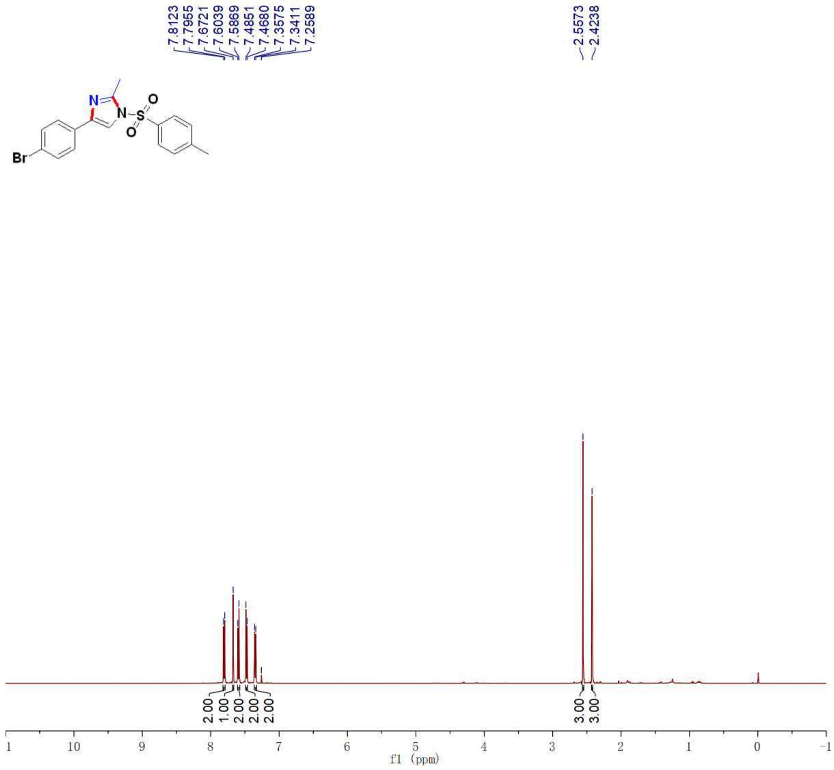

Parameter		Value
1	Data File Name	D:/ 核磁数据/ nmr/ nmr/ 2020-1-syj-H/ 17/ fid
2	标题	2020-1-syj-H.17.fid
3	Comment	1H ztt
4	Origin	Bruker BioSpin GmbH
5	Owner	root
6	Site	
7	Instrument	spect
8	Author	
9	Solvent	CDCl3
10	Temperature	294.6
11	Pulse Sequence	zg30
12	Experiment	1D
13	Probe	Z119470_0117 (PA BBO 500S1 BBF-H-D-05 Z SP)
14	Number of Scans	2
15	Receiver Gain	48.1
16	Relaxation Delay	2.0000
17	Pulse Width	12.0000
18	Presaturation Frequency	
19	Acquisition Time	1.8175
20	Acquisition Date	2019-12-30T11:03:18
21	Modification Date	2019-12-30T11:03:20
22	Class	
23	Spectrometer Frequency	500.16
24	Spectral Width	9014.4
25	Lowest Frequency	-1519.4
26	Nucleus	1H
27	Acquired Size	16384
28	Spectral Size	65536

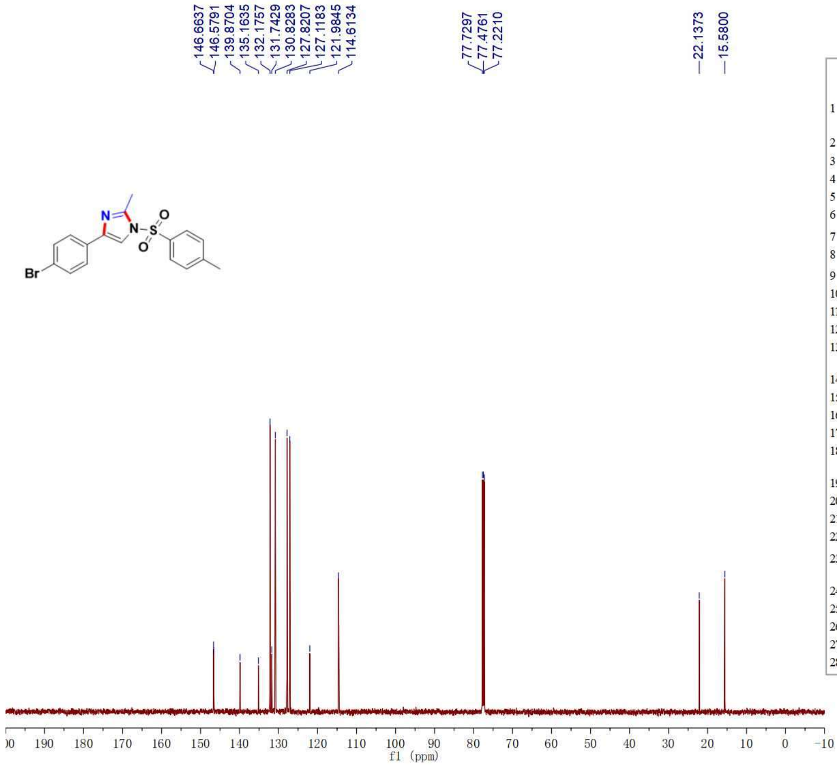

Parameter		Value
1	Data File Name	D:/ 核磁数据/ nmr/ nmr/ 2020-1-syj-C/ 2/ fid
2	标题	2020-1-syj-C.2.fid
3	Comment	13C ztt
4	Origin	Bruker BioSpin GmbH
5	Owner	root
6	Site	
7	Instrument	spect
8	Author	
9	Solvent	CDCl3
10	Temperature	295.0
11	Pulse Sequence	zgpg30
12	Experiment	1D
13	Probe	Z119470_0117 (PA BBO 500S1 BBF-H-D-05 Z SP)
14	Number of Scans	60
15	Receiver Gain	188.8
16	Relaxation Delay	2.0000
17	Pulse Width	10.0000
18	Presaturation Frequency	
19	Acquisition Time	0.5505
20	Acquisition Date	2019-12-30T11:38:07
21	Modification Date	2019-12-30T11:38:10
22	Class	
23	Spectrometer Frequency	125.78
24	Spectral Width	29761.9
25	Lowest Frequency	-1627.5
26	Nucleus	13C
27	Acquired Size	16384
28	Spectral Size	65536

4-(4-(*tert*-Butyl)phenyl)-2-methyl-1-tosyl-1*H*-imidazole (3h)

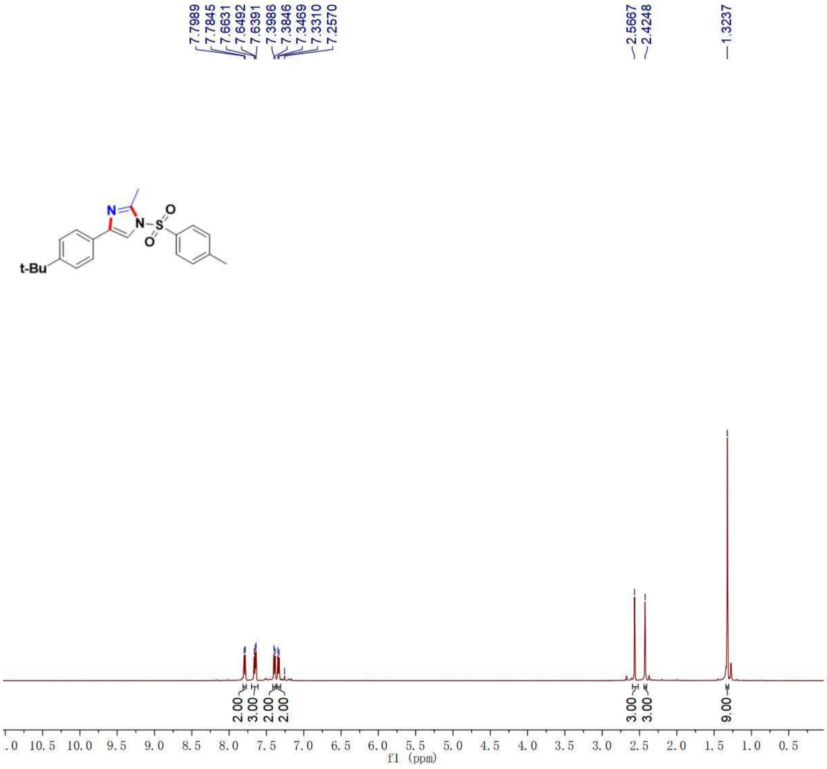

Parameter		Value
1	Data File Name	D:/ 核磁数据/ 20201011/ 2020-1-syj-H/ 284/ fid
2	标题	2020-1-syj-H.284.fid
3	Comment	1H ztt
4	Origin	Bruker BioSpin GmbH
5	Owner	nmrsu
6	Site	
7	Instrument	spect
8	Author	
9	Solvent	CDCl3
10	Temperature	301.3
11	Pulse Sequence	zg30
12	Experiment	1D
13	Probe	Z119470_0117 (PA BBO 500S1 BBF-H-D-05 Z SP)
14	Number of Scans	2
15	Receiver Gain	38.5
16	Relaxation Delay	2.0000
17	Pulse Width	12.0000
18	Presaturation Frequency	
19	Acquisition Time	1.8175
20	Acquisition Date	2020-08-03T19:54:48
21	Modification Date	2020-08-03T19:54:50
22	Class	
23	Spectrometer Frequency	500.16
24	Spectral Width	9014.4
25	Lowest Frequency	-1519.4
26	Nucleus	1H
27	Acquired Size	16384
28	Spectral Size	65536

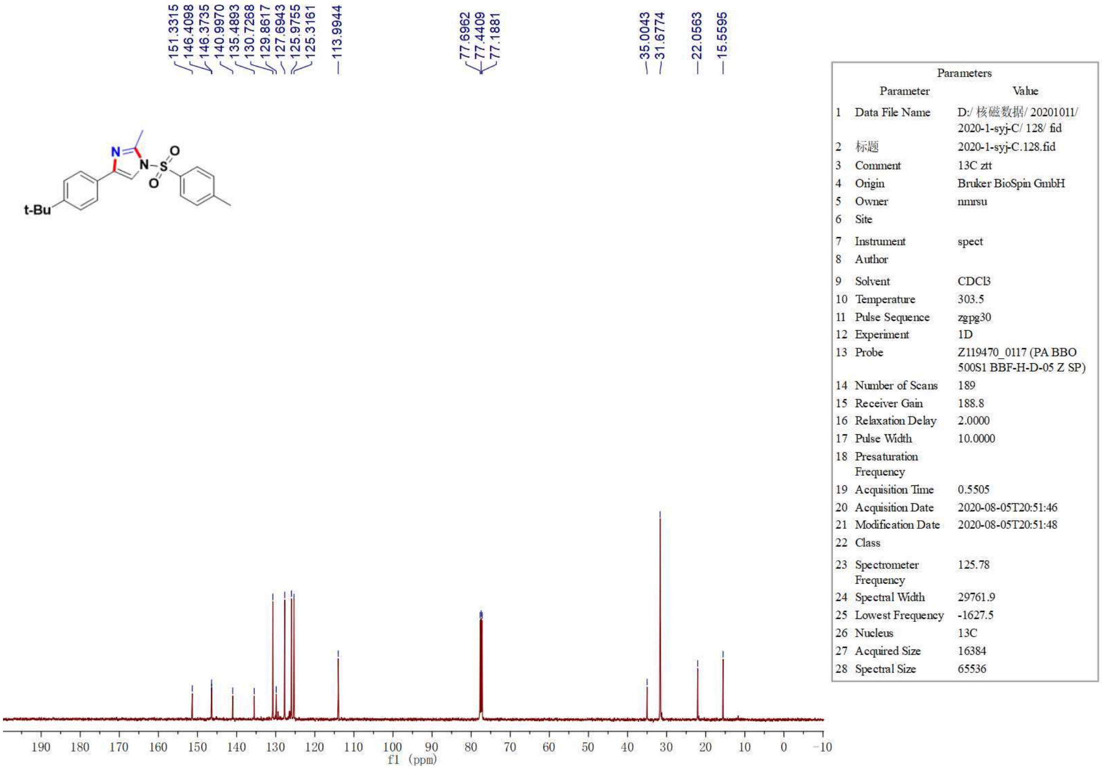

Parameter		Value
1	Data File Name	D:/ 核磁数据/ 20201011/ 2020-1-syj-C/ 128/ fid
2	标题	2020-1-syj-C.128.fid
3	Comment	13C ztt
4	Origin	Bruker BioSpin GmbH
5	Owner	nmrsu
6	Site	
7	Instrument	spect
8	Author	
9	Solvent	CDCl3
10	Temperature	303.5
11	Pulse Sequence	zgpg30
12	Experiment	1D
13	Probe	Z119470_0117 (PA BBO 500S1 BBF-H-D-05 Z SP)
14	Number of Scans	189
15	Receiver Gain	188.8
16	Relaxation Delay	2.0000
17	Pulse Width	10.0000
18	Presaturation Frequency	
19	Acquisition Time	0.5505
20	Acquisition Date	2020-08-05T20:51:46
21	Modification Date	2020-08-05T20:51:48
22	Class	
23	Spectrometer Frequency	125.78
24	Spectral Width	29761.9
25	Lowest Frequency	-1627.5
26	Nucleus	13C
27	Acquired Size	16384
28	Spectral Size	65536

2-Methyl-1-tosyl-4-(4-(trifluoromethyl)phenyl)-1*H*-imidazole (3i)

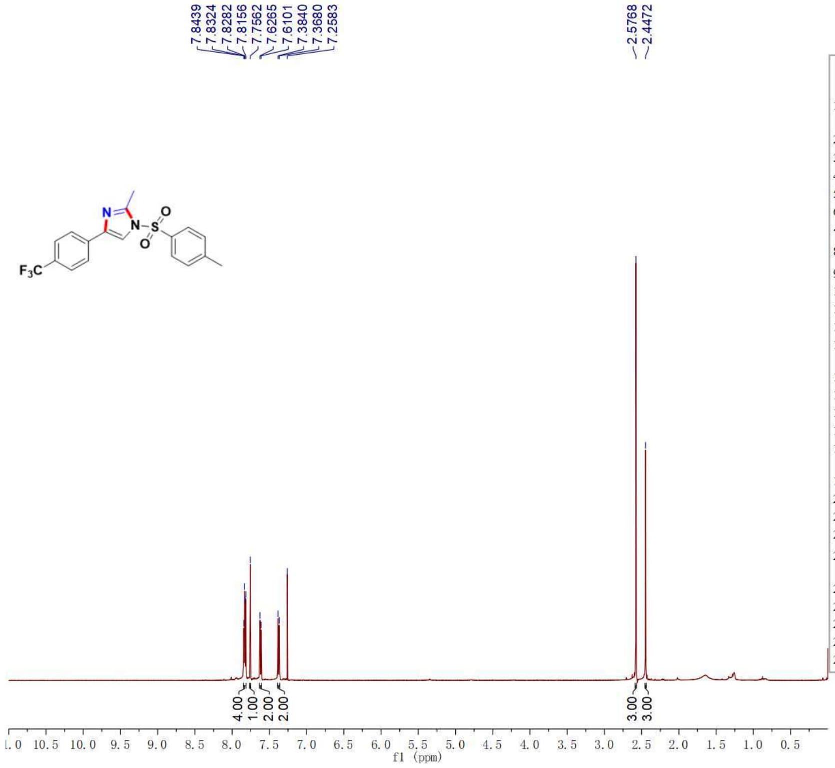

Parameters		
	Parameter	Value
1	Data File Name	D:/ 核磁数据/ 20201011/ 2020-1-syj-H/ 412/ fid
2	标题	2020-1-syj-H.412.fid
3	Comment	1H ztt
4	Origin	Bruker BioSpin GmbH
5	Owner	nmrsu
6	Site	
7	Instrument	spect
8	Author	
9	Solvent	CDCl3
10	Temperature	296.8
11	Pulse Sequence	zg30
12	Experiment	1D
13	Probe	Z119470_0117 (PA BBO 500S1 BBF-H-D-05 Z SP)
14	Number of Scans	2
15	Receiver Gain	150.9
16	Relaxation Delay	2.0000
17	Pulse Width	12.0000
18	Presaturation Frequency	
19	Acquisition Time	1.8175
20	Acquisition Date	2020-09-27T10:16:24
21	Modification Date	2020-09-27T10:16:26
22	Class	
23	Spectrometer Frequency	500.16
24	Spectral Width	9014.4
25	Lowest Frequency	-1519.4
26	Nucleus	1H
27	Acquired Size	16384
28	Spectral Size	65536

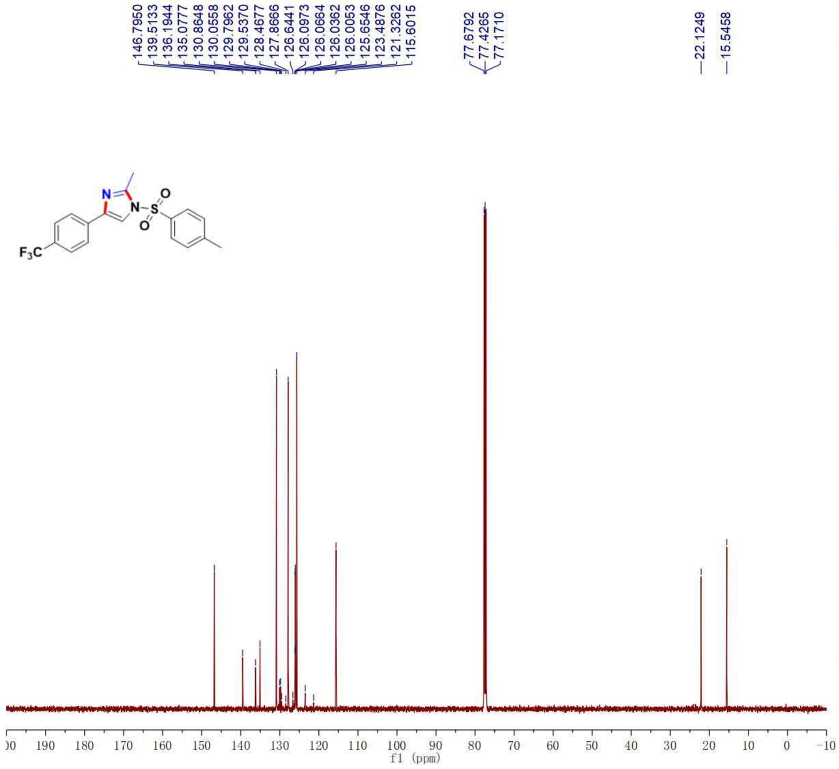

Parameters		
	Parameter	Value
1	Data File Name	D:/ 核磁数据/ nmr/ nmr/ 2020-1-syj-C/ 37/ fid
2	标题	2020-1-syj-C.37.fid
3	Comment	13C ztt
4	Origin	Bruker BioSpin GmbH
5	Owner	nmrsu
6	Site	
7	Instrument	spect
8	Author	
9	Solvent	CDCl3
10	Temperature	295.8
11	Pulse Sequence	zgpg30
12	Experiment	1D
13	Probe	Z119470_0117 (PA BBO 500S1 BBF-H-D-05 Z SP)
14	Number of Scans	434
15	Receiver Gain	188.8
16	Relaxation Delay	2.0000
17	Pulse Width	10.0000
18	Presaturation Frequency	
19	Acquisition Time	0.5505
20	Acquisition Date	2020-05-29T11:02:37
21	Modification Date	2020-05-29T11:02:38
22	Class	
23	Spectrometer Frequency	125.78
24	Spectral Width	29761.9
25	Lowest Frequency	-1627.5
26	Nucleus	13C
27	Acquired Size	16384
28	Spectral Size	65536

2-Methyl-4-(4'-propyl-[1, 1'-biphenyl]-4-yl)-1-tosyl-1*H*-imidazole (3j)

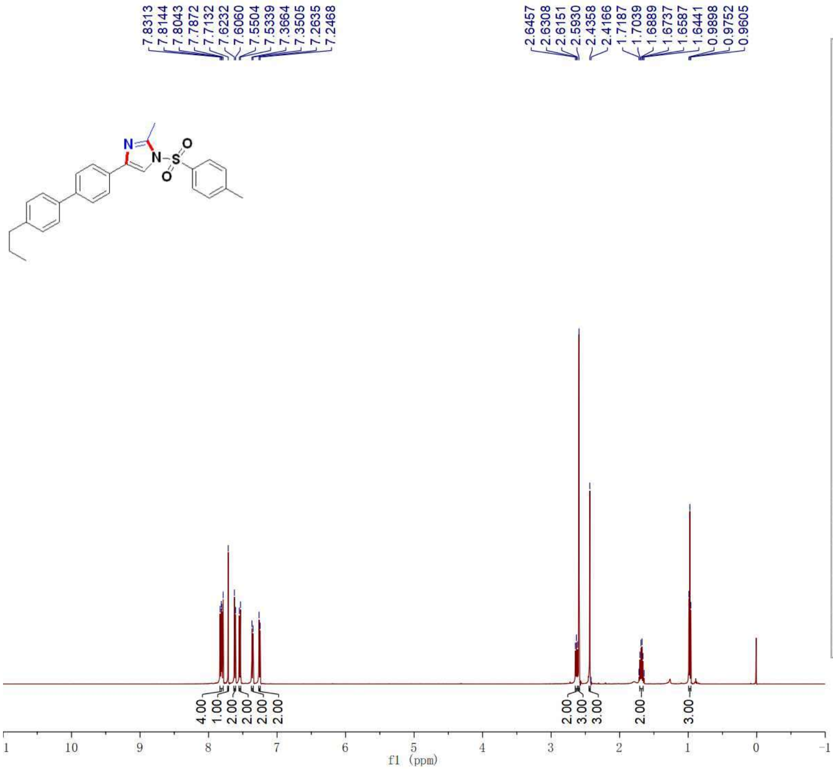

Parameter		Value
1	Data File Name	D:/ 核磁数据/ nmr/ nmr/ 2020-1-syj-H/ 112/ fid
2	标题	2020-1-syj-H.112.fid
3	Comment	1H ztt
4	Origin	Bruker BioSpin GmbH
5	Owner	nmrsu
6	Site	
7	Instrument	spect
8	Author	
9	Solvent	CDCl3
10	Temperature	295.5
11	Pulse Sequence	zg30
12	Experiment	1D
13	Probe	Z119470_0117 (PA BBO 500S1 BBF-H-D-05 Z SP)
14	Number of Scans	2
15	Receiver Gain	38.5
16	Relaxation Delay	2.0000
17	Pulse Width	12.0000
18	Presaturation Frequency	
19	Acquisition Time	1.8175
20	Acquisition Date	2020-06-01T10:28:16
21	Modification Date	2020-06-01T10:28:18
22	Class	
23	Spectrometer Frequency	500.16
24	Spectral Width	9014.4
25	Lowest Frequency	-1519.4
26	Nucleus	1H
27	Acquired Size	16384
28	Spectral Size	65536

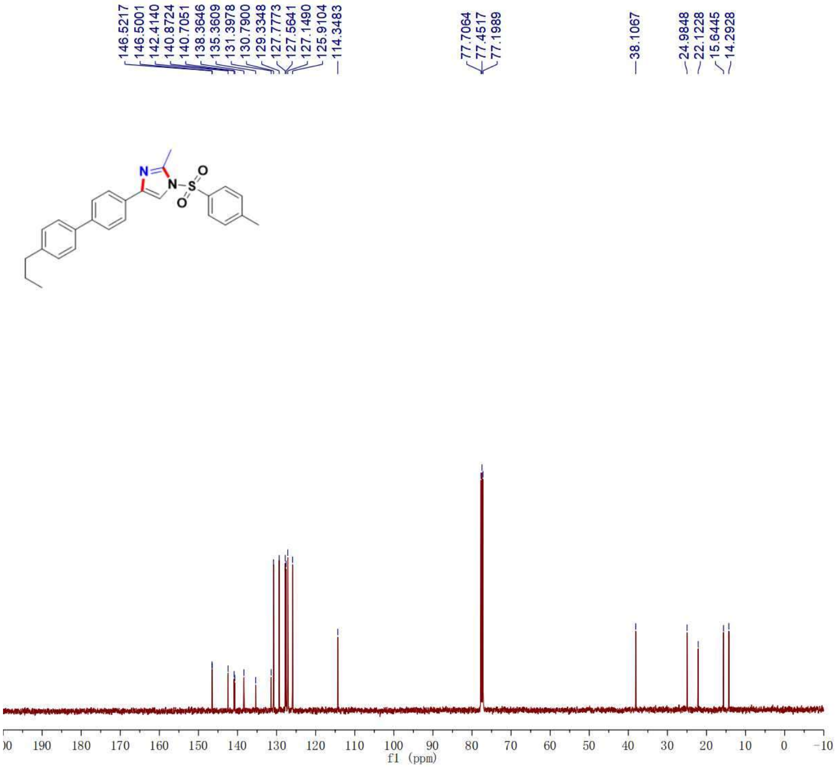

Parameter		Value
1	Data File Name	D:/ 核磁数据/ nmr/ nmr/ 2020-1-syj-C/ 40/ fid
2	标题	2020-1-syj-C.40.fid
3	Comment	13C ztt
4	Origin	Bruker BioSpin GmbH
5	Owner	nmrsu
6	Site	
7	Instrument	spect
8	Author	
9	Solvent	CDCl3
10	Temperature	295.8
11	Pulse Sequence	zgpg30
12	Experiment	1D
13	Probe	Z119470_0117 (PA BBO 500S1 BBF-H-D-05 Z SP)
14	Number of Scans	71
15	Receiver Gain	188.8
16	Relaxation Delay	2.0000
17	Pulse Width	10.0000
18	Presaturation Frequency	
19	Acquisition Time	0.5505
20	Acquisition Date	2020-06-01T10:33:47
21	Modification Date	2020-06-01T10:33:50
22	Class	
23	Spectrometer Frequency	125.78
24	Spectral Width	29761.9
25	Lowest Frequency	-1627.5
26	Nucleus	13C
27	Acquired Size	16384
28	Spectral Size	65536

2-Methyl-4-(*m*-tolyl)-1-tosyl-1*H*-imidazole (3k)

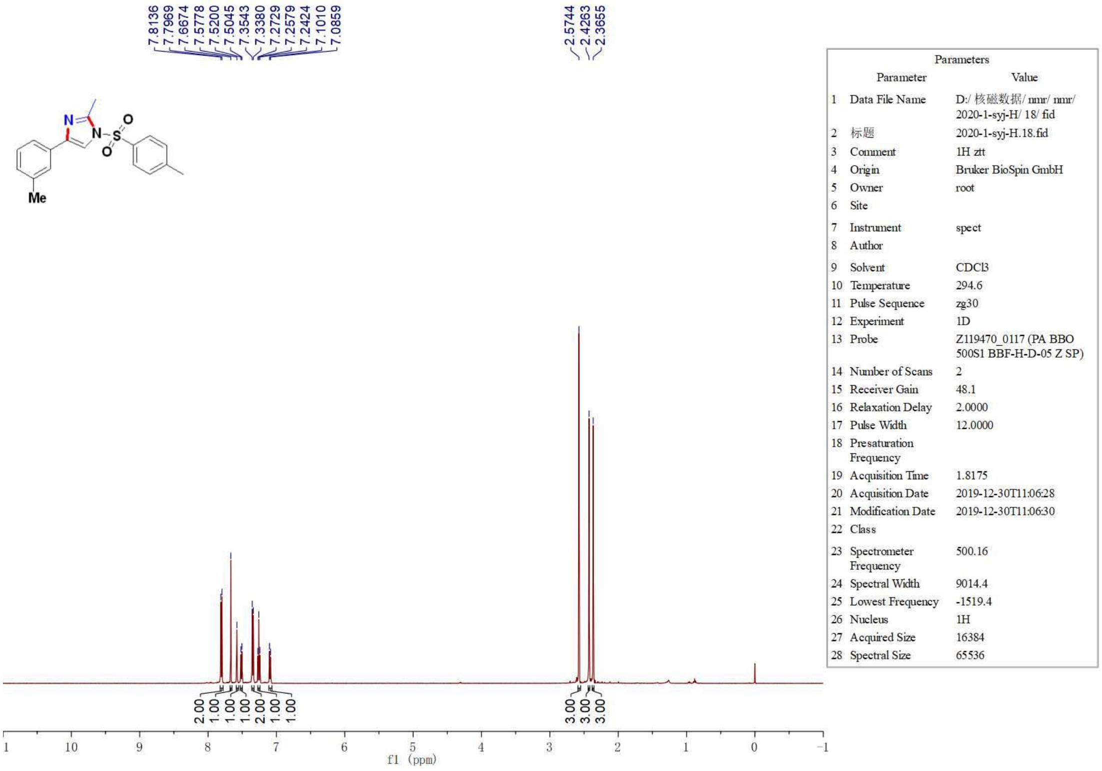

Parameter		Value
1	Data File Name	D:/ 核磁数据/ nmr/ nmr/ 2020-1-syj-H/ 18/ fid
2	标题	2020-1-syj-H.18.fid
3	Comment	1H ztt
4	Origin	Bruker BioSpin GmbH
5	Owner	root
6	Site	
7	Instrument	spect
8	Author	
9	Solvent	CDCl3
10	Temperature	294.6
11	Pulse Sequence	zg30
12	Experiment	1D
13	Probe	Z119470_0117 (PA BBO 500S1 BBF-H-D-05 Z SP)
14	Number of Scans	2
15	Receiver Gain	48.1
16	Relaxation Delay	2.0000
17	Pulse Width	12.0000
18	Presaturation Frequency	
19	Acquisition Time	1.8175
20	Acquisition Date	2019-12-30T11:06:28
21	Modification Date	2019-12-30T11:06:30
22	Class	
23	Spectrometer Frequency	500.16
24	Spectral Width	9014.4
25	Lowest Frequency	-1519.4
26	Nucleus	1H
27	Acquired Size	16384
28	Spectral Size	65536

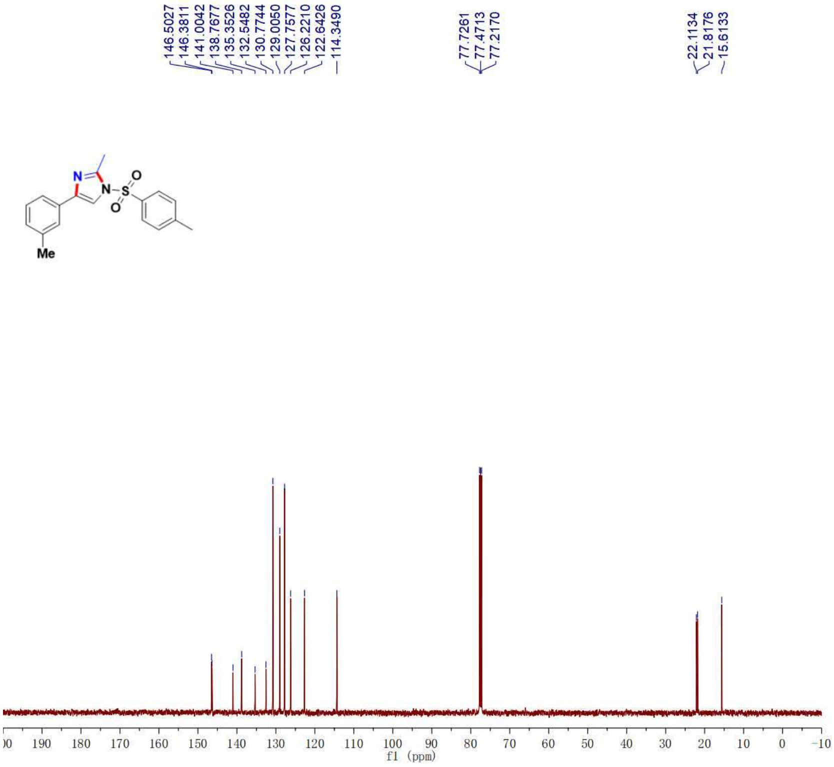

Parameter		Value
1	Data File Name	D:/ 核磁数据/ nmr/ nmr/ 2020-1-syj-C/ 3/ fid
2	标题	2020-1-syj-C.3.fid
3	Comment	13C ztt
4	Origin	Bruker BioSpin GmbH
5	Owner	root
6	Site	
7	Instrument	spect
8	Author	
9	Solvent	CDCl3
10	Temperature	295.4
11	Pulse Sequence	zgpg30
12	Experiment	1D
13	Probe	Z119470_0117 (PA BBO 500S1 BBF-H-D-05 Z SP)
14	Number of Scans	60
15	Receiver Gain	188.8
16	Relaxation Delay	2.0000
17	Pulse Width	10.0000
18	Presaturation Frequency	
19	Acquisition Time	0.5505
20	Acquisition Date	2019-12-30T11:43:02
21	Modification Date	2019-12-30T11:43:04
22	Class	
23	Spectrometer Frequency	125.78
24	Spectral Width	29761.9
25	Lowest Frequency	-1627.5
26	Nucleus	13C
27	Acquired Size	16384
28	Spectral Size	65536

4-(3-Chlorophenyl)-2-methyl-1-tosyl-1*H*-imidazole (3l)

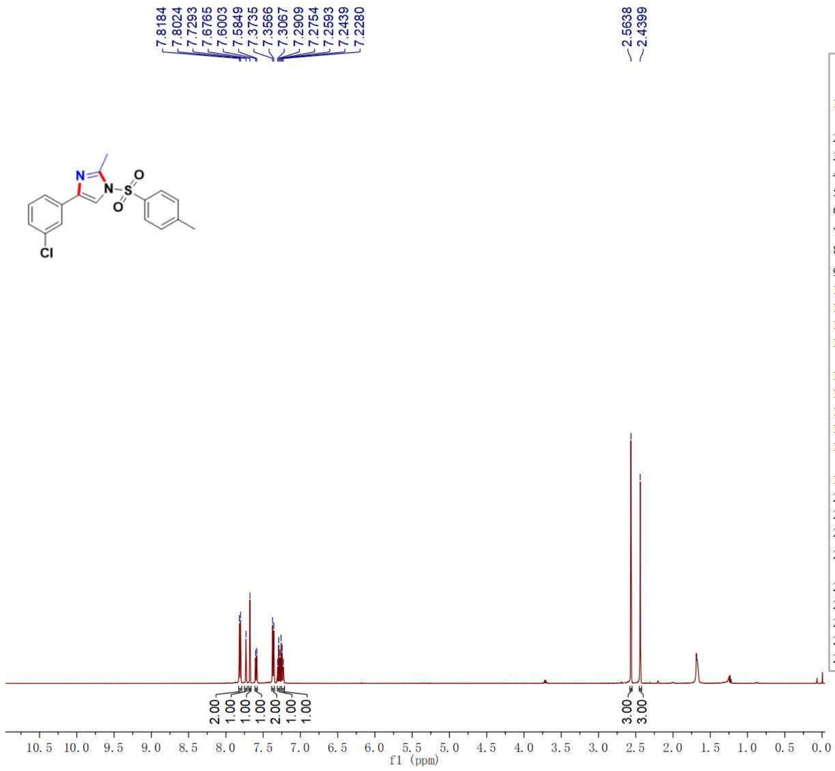

Parameters		
	Parameter	Value
1	Data File Name	D:/ 核磁数据/ 20201011/ 2020-1-syj-H/ 251/ fid
2	标题	2020-1-syj-H.251.fid
3	Comment	1H ztt
4	Origin	Bruker BioSpin GmbH
5	Owner	nmrsu
6	Site	
7	Instrument	spect
8	Author	
9	Solvent	CDCl3
10	Temperature	297.5
11	Pulse Sequence	zg30
12	Experiment	1D
13	Probe	Z119470_0117 (PA BBO 500S1 BBF-H-D-05 Z SP)
14	Number of Scans	2
15	Receiver Gain	108.7
16	Relaxation Delay	2.0000
17	Pulse Width	12.0000
18	Presaturation Frequency	
19	Acquisition Time	1.8175
20	Acquisition Date	2020-07-19T21:15:02
21	Modification Date	2020-07-19T21:15:04
22	Class	
23	Spectrometer Frequency	500.16
24	Spectral Width	9014.4
25	Lowest Frequency	-1519.4
26	Nucleus	1H
27	Acquired Size	16384
28	Spectral Size	65536

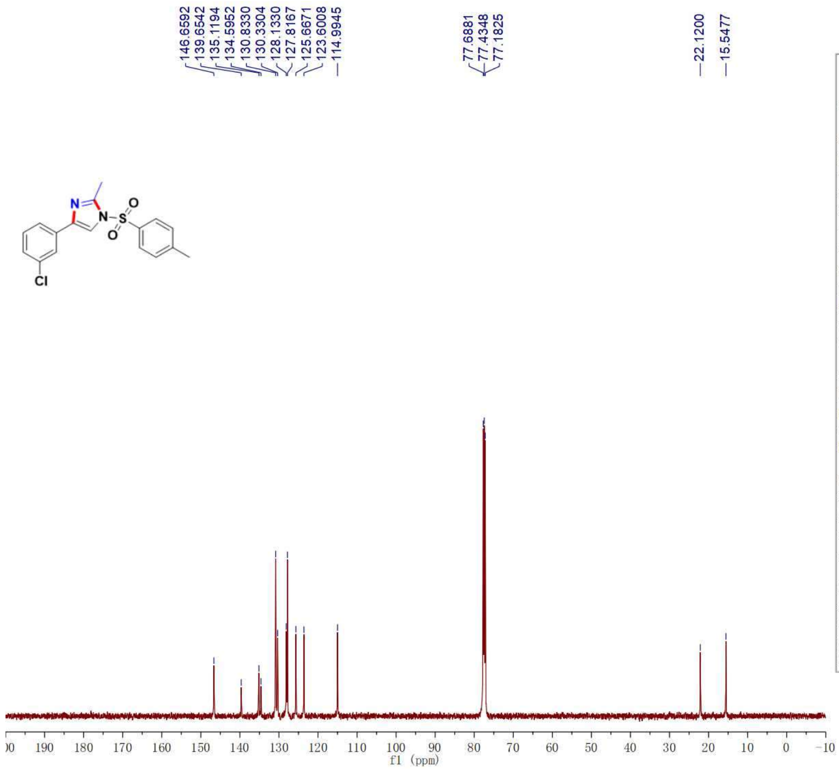

Parameters		
	Parameter	Value
1	Data File Name	D:/ 核磁数据/ 20201011/ 2020-1-syj-C/ 97/ fid
2	标题	2020-1-syj-C.97.fid
3	Comment	13C ztt
4	Origin	Bruker BioSpin GmbH
5	Owner	nmrsu
6	Site	
7	Instrument	spect
8	Author	
9	Solvent	CDCl3
10	Temperature	297.3
11	Pulse Sequence	zgpg30
12	Experiment	1D
13	Probe	Z119470_0117 (PA BBO 500S1 BBF-H-D-05 Z SP)
14	Number of Scans	2000
15	Receiver Gain	188.8
16	Relaxation Delay	2.0000
17	Pulse Width	10.0000
18	Presaturation Frequency	
19	Acquisition Time	0.5505
20	Acquisition Date	2020-07-17T13:43:42
21	Modification Date	2020-07-17T13:43:44
22	Class	
23	Spectrometer Frequency	125.78
24	Spectral Width	29761.9
25	Lowest Frequency	-1627.5
26	Nucleus	13C
27	Acquired Size	16384
28	Spectral Size	65536

4-(3-Bromophenyl)-2-methyl-1-tosyl-1*H*-imidazole (3m)

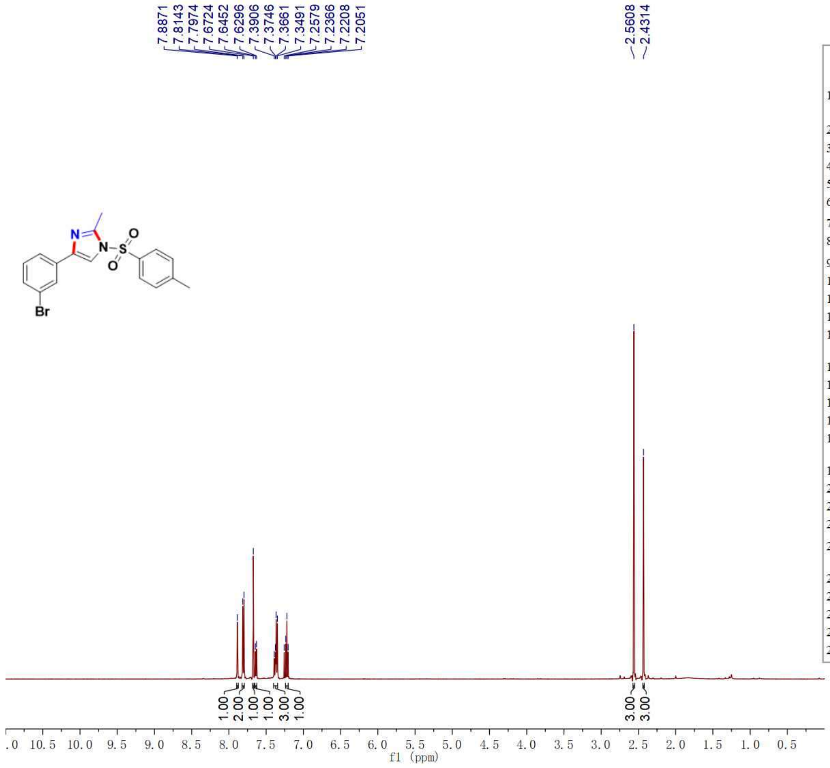

	Parameter	Value
1	Data File Name	D:/ 核磁数据/ 20201011/ 2020-1-syj-H/ 275/ fid
2	标题	2020-1-syj-H.275.fid
3	Comment	1H ztt
4	Origin	Bruker BioSpin GmbH
5	Owner	nmrsu
6	Site	
7	Instrument	spect
8	Author	
9	Solvent	CDCl3
10	Temperature	299.0
11	Pulse Sequence	zg30
12	Experiment	1D
13	Probe	Z119470_0117 (PA BBO 500S1 BBF-H-D-05 Z SP)
14	Number of Scans	2
15	Receiver Gain	66.5
16	Relaxation Delay	2.0000
17	Pulse Width	12.0000
18	Presaturation Frequency	
19	Acquisition Time	1.8175
20	Acquisition Date	2020-08-01T10:49:13
21	Modification Date	2020-08-01T10:49:14
22	Class	
23	Spectrometer Frequency	500.16
24	Spectral Width	9014.4
25	Lowest Frequency	-1519.4
26	Nucleus	1H
27	Acquired Size	16384
28	Spectral Size	65536

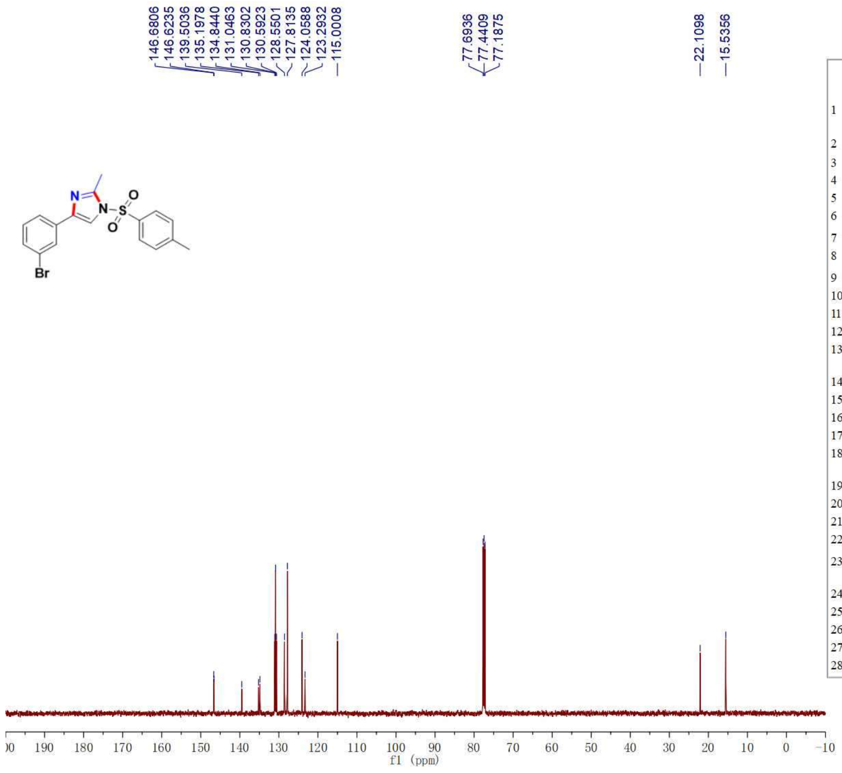

	Parameter	Value
1	Data File Name	D:/ 核磁数据/ 20201011/ 2020-1-syj-C/ 118/ fid
2	标题	2020-1-syj-C.118.fid
3	Comment	13C ztt
4	Origin	Bruker BioSpin GmbH
5	Owner	nmrsu
6	Site	
7	Instrument	spect
8	Author	
9	Solvent	CDCl3
10	Temperature	300.6
11	Pulse Sequence	zgpg30
12	Experiment	1D
13	Probe	Z119470_0117 (PA BBO 500S1 BBF-H-D-05 Z SP)
14	Number of Scans	60
15	Receiver Gain	188.8
16	Relaxation Delay	2.0000
17	Pulse Width	10.0000
18	Presaturation Frequency	
19	Acquisition Time	0.5505
20	Acquisition Date	2020-08-01T16:37:30
21	Modification Date	2020-08-01T16:37:32
22	Class	
23	Spectrometer Frequency	125.78
24	Spectral Width	29761.9
25	Lowest Frequency	-1627.5
26	Nucleus	13C
27	Acquired Size	16384
28	Spectral Size	65536

4-(2-Fluorophenyl)-2-methyl-1-tosyl-1H-imidazole (3n)

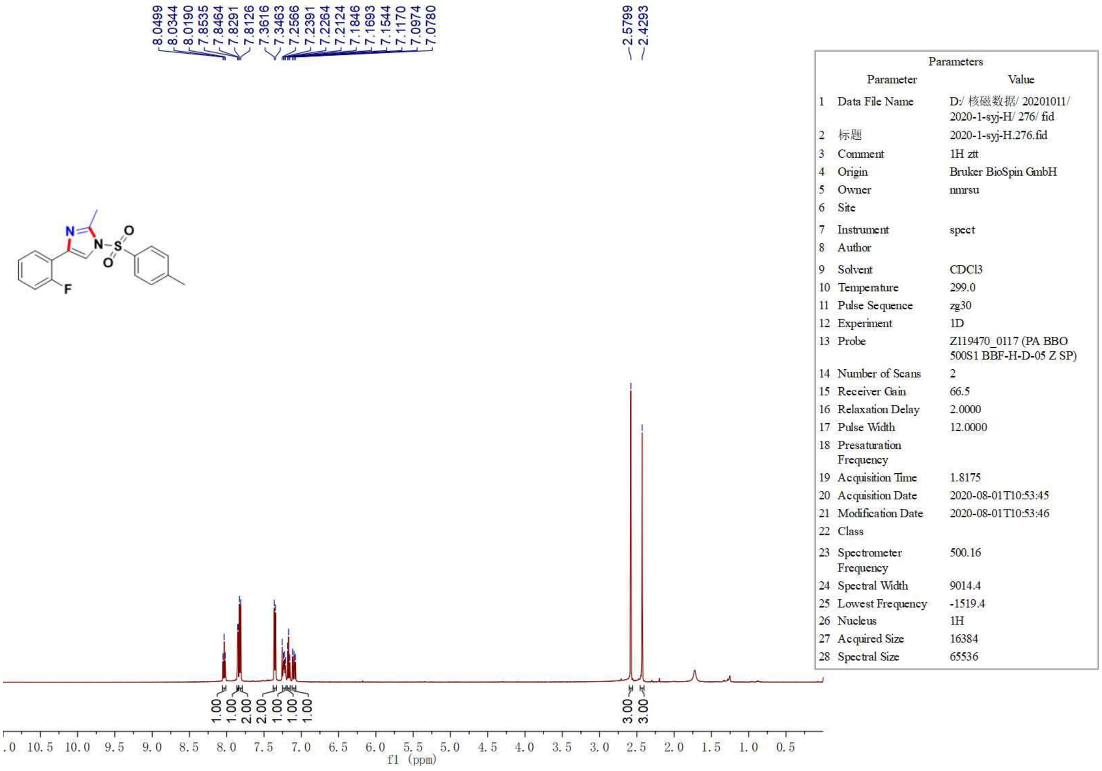

Parameters

	Parameter	Value
1	Data File Name	D:/ 核磁数据/ 20201011/ 2020-1-syj-H/ 276/ fid
2	标题	2020-1-syj-H.276.fid
3	Comment	1H ztt
4	Origin	Bruker BioSpin GmbH
5	Owner	nmrsu
6	Site	
7	Instrument	spect
8	Author	
9	Solvent	CDCl3
10	Temperature	299.0
11	Pulse Sequence	zg30
12	Experiment	1D
13	Probe	Z119470_0117 (PA BBO 500S1 BBF-H-D-05 Z SP)
14	Number of Scans	2
15	Receiver Gain	66.5
16	Relaxation Delay	2.0000
17	Pulse Width	12.0000
18	Presaturation Frequency	
19	Acquisition Time	1.8175
20	Acquisition Date	2020-08-01T10:53:45
21	Modification Date	2020-08-01T10:53:46
22	Class	
23	Spectrometer Frequency	500.16
24	Spectral Width	9014.4
25	Lowest Frequency	-1519.4
26	Nucleus	1H
27	Acquired Size	16384
28	Spectral Size	65536

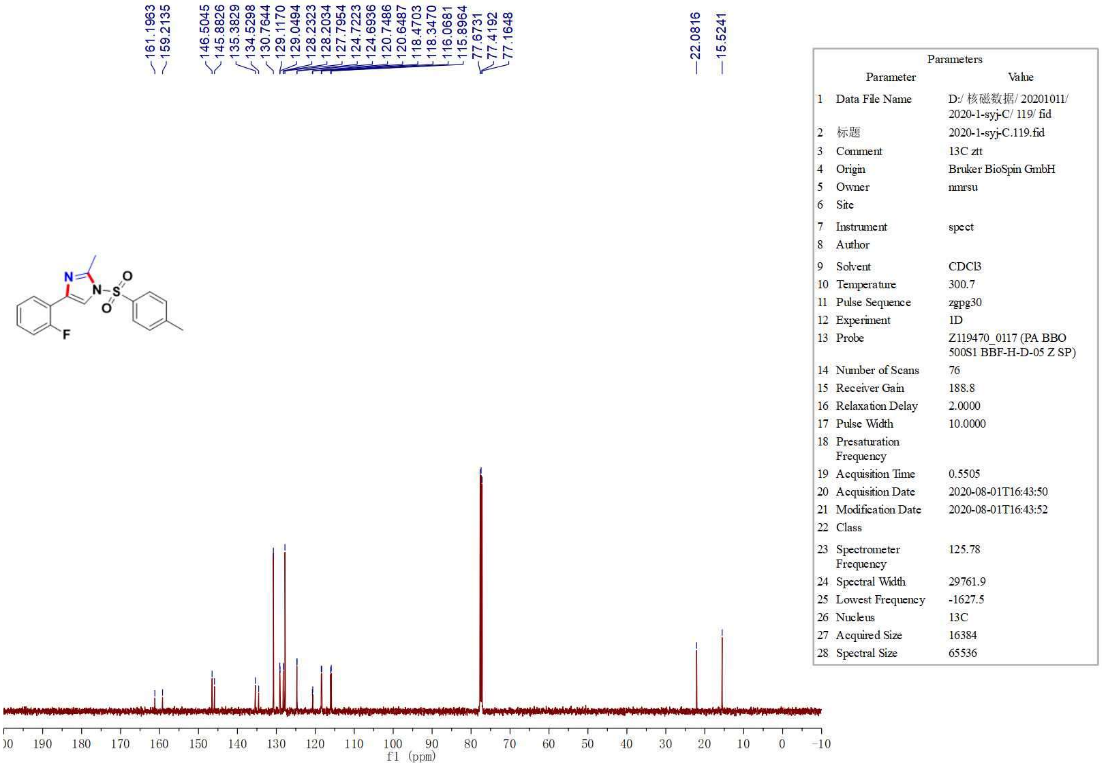

Parameters

	Parameter	Value
1	Data File Name	D:/ 核磁数据/ 20201011/ 2020-1-syj-C/ 119/ fid
2	标题	2020-1-syj-C.119.fid
3	Comment	13C ztt
4	Origin	Bruker BioSpin GmbH
5	Owner	nmrsu
6	Site	
7	Instrument	spect
8	Author	
9	Solvent	CDCl3
10	Temperature	300.7
11	Pulse Sequence	zgpg30
12	Experiment	1D
13	Probe	Z119470_0117 (PA BBO 500S1 BBF-H-D-05 Z SP)
14	Number of Scans	76
15	Receiver Gain	188.8
16	Relaxation Delay	2.0000
17	Pulse Width	10.0000
18	Presaturation Frequency	
19	Acquisition Time	0.5505
20	Acquisition Date	2020-08-01T16:43:50
21	Modification Date	2020-08-01T16:43:52
22	Class	
23	Spectrometer Frequency	125.78
24	Spectral Width	29761.9
25	Lowest Frequency	-1627.5
26	Nucleus	13C
27	Acquired Size	16384
28	Spectral Size	65536

1-((4-Fluorophenyl)sulfonyl)-2-methyl-4-phenyl-1*H*-imidazole (3o)

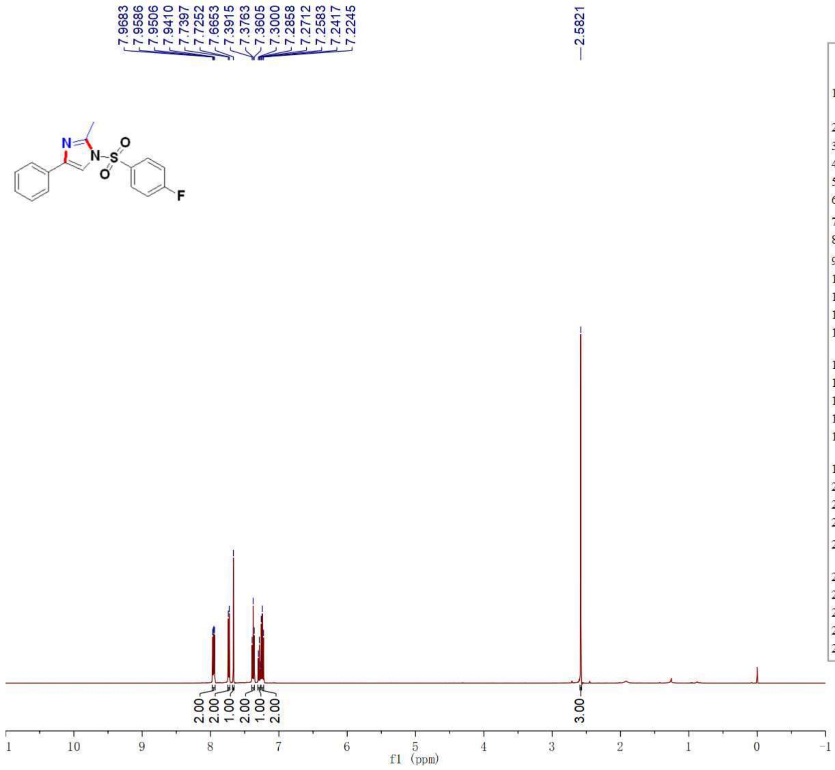

Parameter		Value
1	Data File Name	D:/ 核磁数据/ nmr/ nmr/ 2020-1-syj-H/ 114/ fid
2	标题	2020-1-syj-H.114.fid
3	Comment	1H ztt
4	Origin	Bruker BioSpin GmbH
5	Owner	nmrsu
6	Site	
7	Instrument	spect
8	Author	
9	Solvent	CDCl3
10	Temperature	295.7
11	Pulse Sequence	zg30
12	Experiment	1D
13	Probe	Z119470_0117 (PA BBO 500S1 BBF-H-D-05 Z SP)
14	Number of Scans	2
15	Receiver Gain	59.2
16	Relaxation Delay	2.0000
17	Pulse Width	12.0000
18	Presaturation Frequency	
19	Acquisition Time	1.8175
20	Acquisition Date	2020-06-01T10:45:16
21	Modification Date	2020-06-01T10:45:18
22	Class	
23	Spectrometer Frequency	500.16
24	Spectral Width	9014.4
25	Lowest Frequency	-1519.4
26	Nucleus	1H
27	Acquired Size	16384
28	Spectral Size	65536

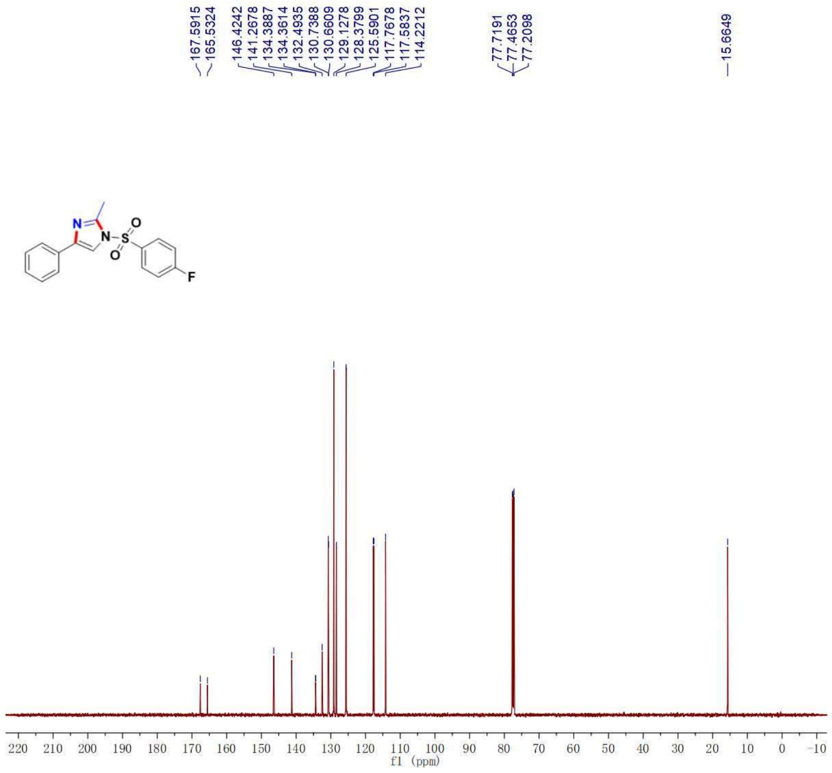

Parameter		Value
1	Data File Name	D:/ 核磁数据/ nmr/ nmr/ 2020-1-syj-C/ 42/ fid
2	标题	2020-1-syj-C.42.fid
3	Comment	13C ztt
4	Origin	Bruker BioSpin GmbH
5	Owner	nmrsu
6	Site	
7	Instrument	spect
8	Author	
9	Solvent	CDCl3
10	Temperature	295.6
11	Pulse Sequence	zgpg30
12	Experiment	1D
13	Probe	Z119470_0117 (PA BBO 500S1 BBF-H-D-05 Z SP)
14	Number of Scans	294
15	Receiver Gain	188.8
16	Relaxation Delay	2.0000
17	Pulse Width	10.0000
18	Presaturation Frequency	
19	Acquisition Time	0.5505
20	Acquisition Date	2020-06-01T11:00:15
21	Modification Date	2020-06-01T11:00:18
22	Class	
23	Spectrometer Frequency	125.78
24	Spectral Width	29761.9
25	Lowest Frequency	-1627.5
26	Nucleus	13C
27	Acquired Size	16384
28	Spectral Size	65536

1-((4-Bromophenyl)sulfonyl)-2-methyl-4-phenyl-1*H*-imidazole (3p)

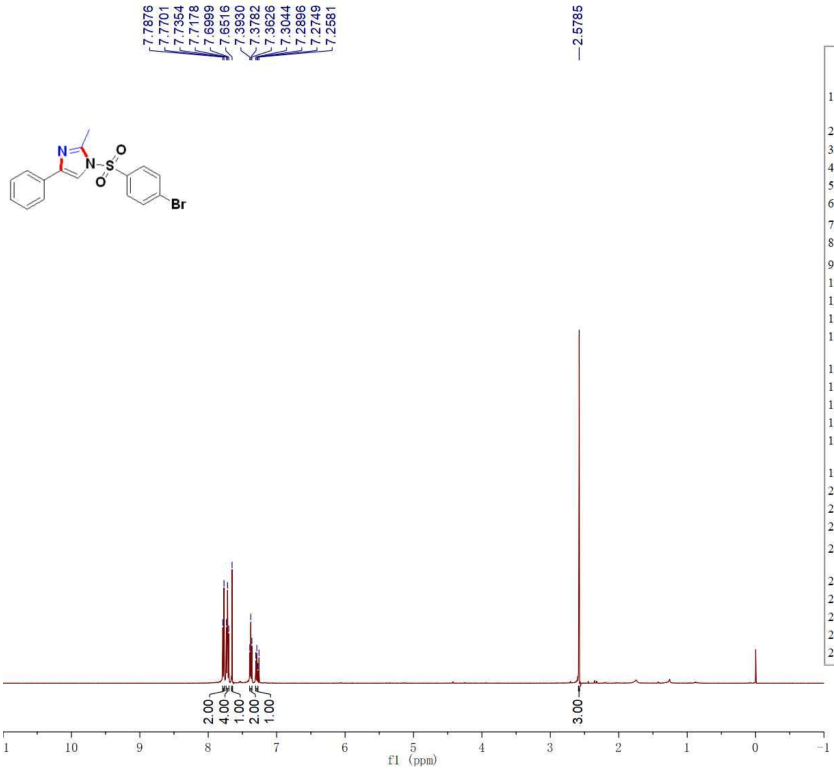

Parameters		
	Parameter	Value
1	Data File Name	D:/ 核磁数据/ nmr/ nmr/ 2020-1-syj-H/ 113/ fid
2	标题	2020-1-syj-H.113.fid
3	Comment	1H ztt
4	Origin	Bruker BioSpin GmbH
5	Owner	nmrsu
6	Site	
7	Instrument	spect
8	Author	
9	Solvent	CDCl3
10	Temperature	295.6
11	Pulse Sequence	zg30
12	Experiment	1D
13	Probe	Z119470_0117 (PA BBO 500S1 BBF-H-D-05 Z SP)
14	Number of Scans	2
15	Receiver Gain	87.0
16	Relaxation Delay	2.0000
17	Pulse Width	12.0000
18	Presaturation Frequency	
19	Acquisition Time	1.8175
20	Acquisition Date	2020-06-01T10:36:18
21	Modification Date	2020-06-01T10:36:20
22	Class	
23	Spectrometer Frequency	500.16
24	Spectral Width	9014.4
25	Lowest Frequency	-1519.4
26	Nucleus	1H
27	Acquired Size	16384
28	Spectral Size	65536

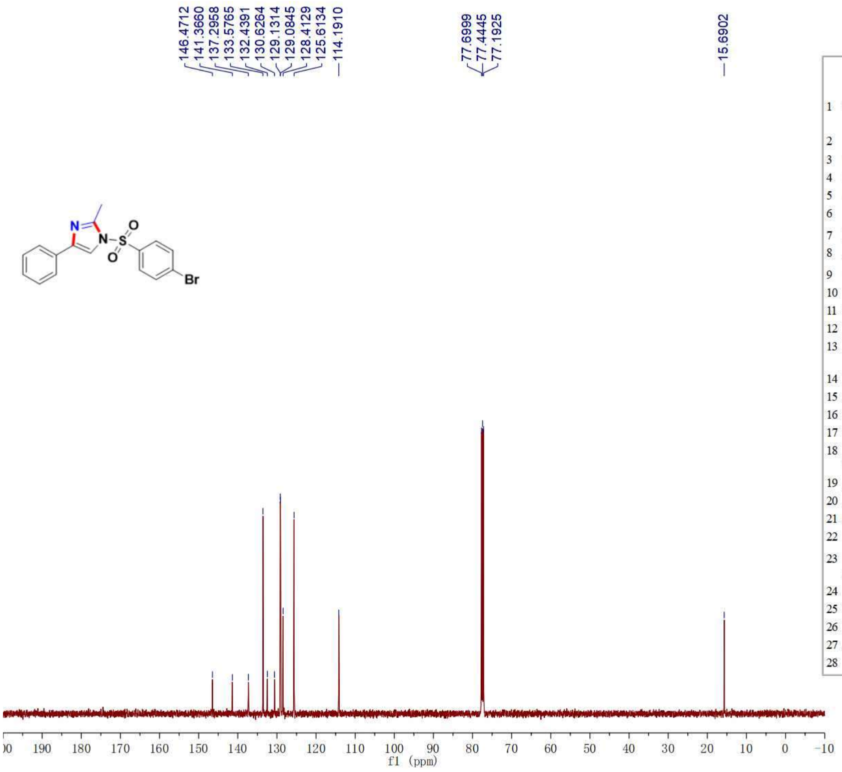

Parameters		
	Parameter	Value
1	Data File Name	D:/ 核磁数据/ nmr/ nmr/ 2020-1-syj-C/ 41/ fid
2	标题	2020-1-syj-C.41.fid
3	Comment	13C ztt
4	Origin	Bruker BioSpin GmbH
5	Owner	nmrsu
6	Site	
7	Instrument	spect
8	Author	
9	Solvent	CDCl3
10	Temperature	295.7
11	Pulse Sequence	zgpg30
12	Experiment	1D
13	Probe	Z119470_0117 (PA BBO 500S1 BBF-H-D-05 Z SP)
14	Number of Scans	100
15	Receiver Gain	188.8
16	Relaxation Delay	2.0000
17	Pulse Width	10.0000
18	Presaturation Frequency	
19	Acquisition Time	0.5505
20	Acquisition Date	2020-06-01T10:42:46
21	Modification Date	2020-06-01T10:42:48
22	Class	
23	Spectrometer Frequency	125.78
24	Spectral Width	29761.9
25	Lowest Frequency	-1627.5
26	Nucleus	13C
27	Acquired Size	16384
28	Spectral Size	65536

2-Ethyl-4-phenyl-1-tosyl-1*H*-imidazole (3q)

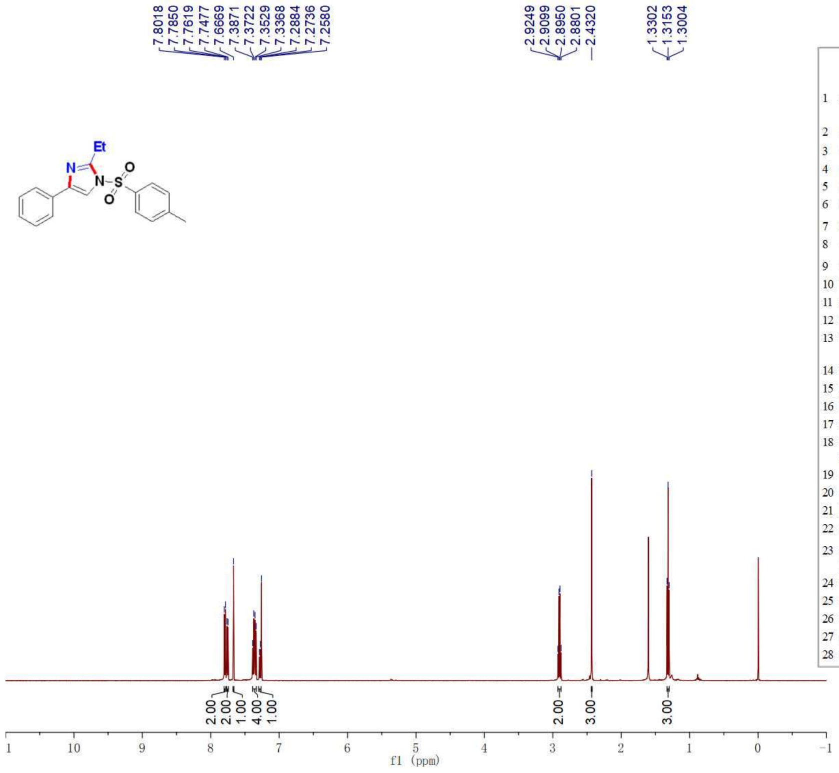

	Parameters	
	Parameter	Value
1	Data File Name	D:/ 核磁数据/ nmr/ nmr/ 2020-1-syj-H/ 226/ fid
2	标题	2020-1-syj-H.226.fid
3	Comment	1H ztt
4	Origin	Bruker BioSpin GmbH
5	Owner	nmrsu
6	Site	
7	Instrument	spect
8	Author	
9	Solvent	CDCl3
10	Temperature	297.4
11	Pulse Sequence	zg30
12	Experiment	1D
13	Probe	Z119470_0117 (PA BBO 500S1 BBF-H-D-05 Z SP)
14	Number of Scans	2
15	Receiver Gain	128.6
16	Relaxation Delay	2.0000
17	Pulse Width	12.0000
18	Presaturation Frequency	
19	Acquisition Time	1.8175
20	Acquisition Date	2020-07-13T17:04:09
21	Modification Date	2020-07-13T17:04:10
22	Class	
23	Spectrometer Frequency	500.16
24	Spectral Width	9014.4
25	Lowest Frequency	-1519.4
26	Nucleus	1H
27	Acquired Size	16384
28	Spectral Size	65536

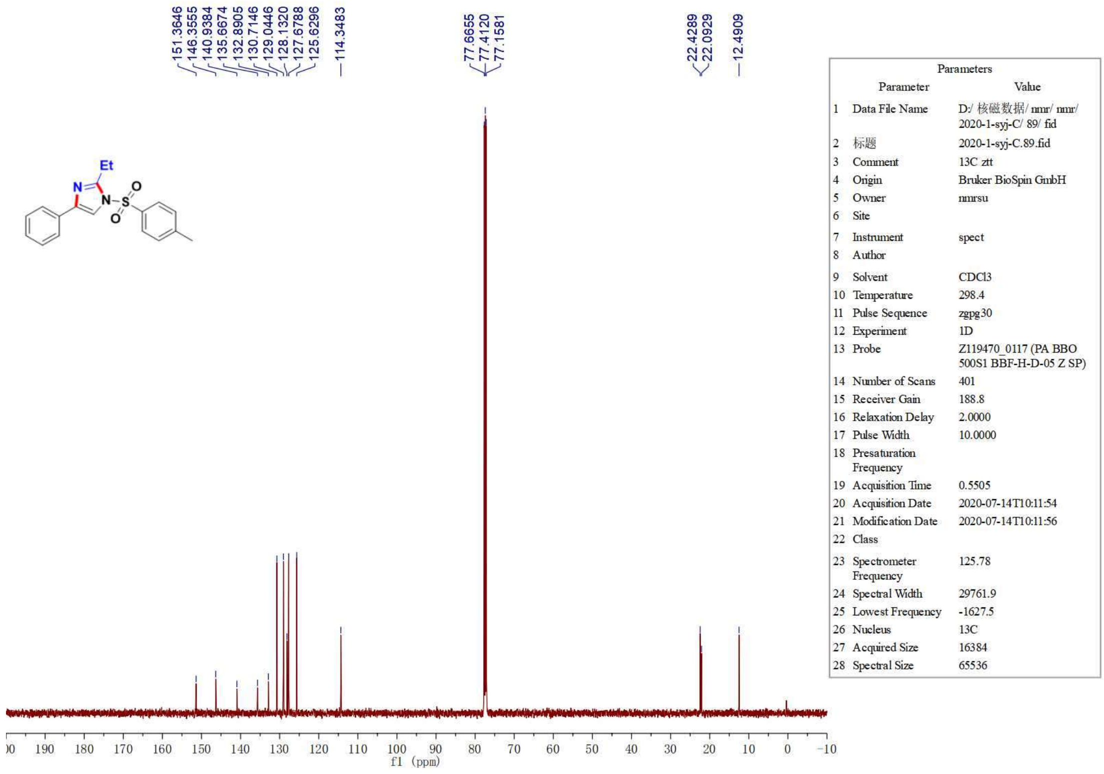

	Parameters	
	Parameter	Value
1	Data File Name	D:/ 核磁数据/ nmr/ nmr/ 2020-1-syj-C/ 89/ fid
2	标题	2020-1-syj-C.89.fid
3	Comment	13C ztt
4	Origin	Bruker BioSpin GmbH
5	Owner	nmrsu
6	Site	
7	Instrument	spect
8	Author	
9	Solvent	CDCl3
10	Temperature	298.4
11	Pulse Sequence	zgpg30
12	Experiment	1D
13	Probe	Z119470_0117 (PA BBO 500S1 BBF-H-D-05 Z SP)
14	Number of Scans	401
15	Receiver Gain	188.8
16	Relaxation Delay	2.0000
17	Pulse Width	10.0000
18	Presaturation Frequency	
19	Acquisition Time	0.5505
20	Acquisition Date	2020-07-14T10:11:54
21	Modification Date	2020-07-14T10:11:56
22	Class	
23	Spectrometer Frequency	125.78
24	Spectral Width	29761.9
25	Lowest Frequency	-1627.5
26	Nucleus	13C
27	Acquired Size	16384
28	Spectral Size	65536

4-Phenyl-2-propyl-1-tosyl-1*H*-imidazole (3r)

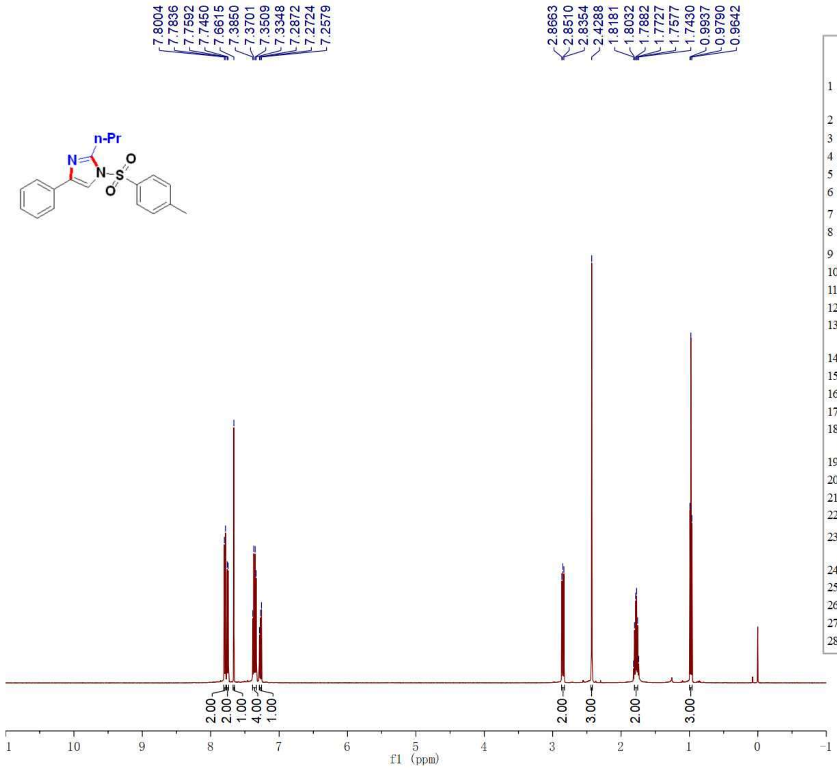

Parameters		
	Parameter	Value
1	Data File Name	D:/ 核磁数据/ nmr/ nmr/ 2020-1-syj-H/ 224/ fid
2	标题	2020-1-syj-H.224.fid
3	Comment	1H ztt
4	Origin	Bruker BioSpin GmbH
5	Owner	nmrsu
6	Site	
7	Instrument	spect
8	Author	
9	Solvent	CDCl3
10	Temperature	297.4
11	Pulse Sequence	zg30
12	Experiment	1D
13	Probe	Z119470_0117 (PA BBO 500S1 BBF-H-D-05 Z SP)
14	Number of Scans	2
15	Receiver Gain	66.5
16	Relaxation Delay	2.0000
17	Pulse Width	12.0000
18	Presaturation Frequency	
19	Acquisition Time	1.8175
20	Acquisition Date	2020-07-13T16:48:36
21	Modification Date	2020-07-13T16:48:38
22	Class	
23	Spectrometer Frequency	500.16
24	Spectral Width	9014.4
25	Lowest Frequency	-1519.4
26	Nucleus	1H
27	Acquired Size	16384
28	Spectral Size	65536

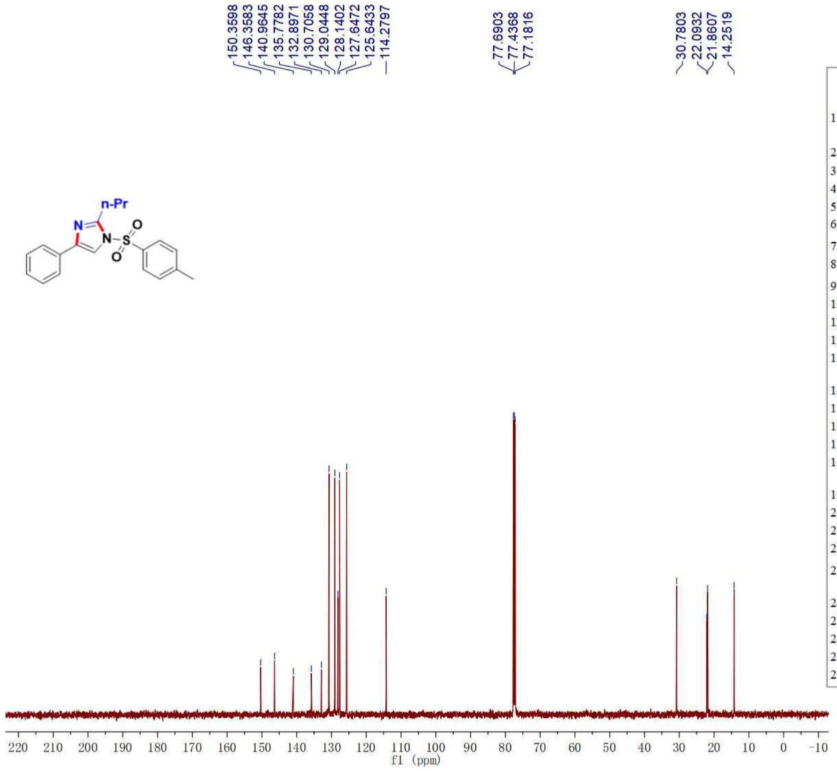

Parameters		
	Parameter	Value
1	Data File Name	D:/ 核磁数据/ nmr/ nmr/ 2020-1-syj-C/ 87/ fid
2	标题	2020-1-syj-C.87.fid
3	Comment	13C ztt
4	Origin	Bruker BioSpin GmbH
5	Owner	nmrsu
6	Site	
7	Instrument	spect
8	Author	
9	Solvent	CDCl3
10	Temperature	297.9
11	Pulse Sequence	zgpg30
12	Experiment	1D
13	Probe	Z119470_0117 (PA BBO 500S1 BBF-H-D-05 Z SP)
14	Number of Scans	100
15	Receiver Gain	188.8
16	Relaxation Delay	2.0000
17	Pulse Width	10.0000
18	Presaturation Frequency	
19	Acquisition Time	0.5505
20	Acquisition Date	2020-07-14T09:18:30
21	Modification Date	2020-07-14T09:18:32
22	Class	
23	Spectrometer Frequency	125.78
24	Spectral Width	29761.9
25	Lowest Frequency	-1627.5
26	Nucleus	13C
27	Acquired Size	16384
28	Spectral Size	65536

2-Butyl-4-phenyl-1-tosyl-1*H*-imidazole (3s)

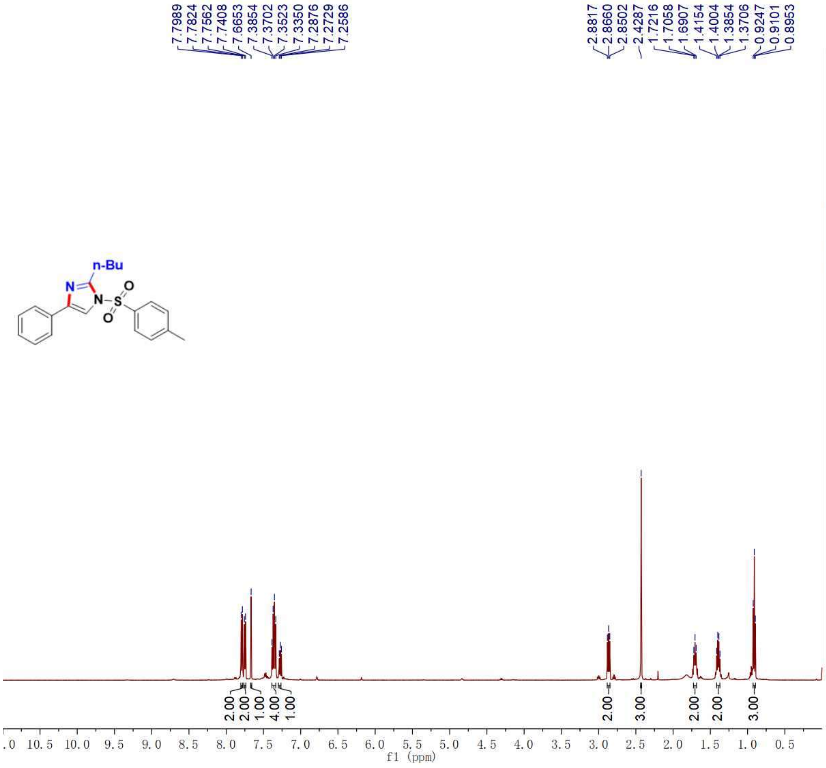

Parameters

	Parameter	Value
1	Data File Name	D:/ 核磁数据/ 20201011/ 2020-1-syj-H/ 294/ fid
2	标题	2020-1-syj-H.294.fid
3	Comment	1H ztt
4	Origin	Bruker BioSpin GmbH
5	Owner	nmrsu
6	Site	
7	Instrument	spect
8	Author	
9	Solvent	CDCl3
10	Temperature	292.9
11	Pulse Sequence	zg30
12	Experiment	1D
13	Probe	Z119470_0117 (PA BBO 500S1 BBF-H-D-05 Z SP)
14	Number of Scans	4
15	Receiver Gain	66.5
16	Relaxation Delay	2.0000
17	Pulse Width	12.0000
18	Presaturation Frequency	
19	Acquisition Time	1.8175
20	Acquisition Date	2020-08-10T11:17:01
21	Modification Date	2020-08-10T11:17:02
22	Class	
23	Spectrometer Frequency	500.16
24	Spectral Width	9014.4
25	Lowest Frequency	-1519.4
26	Nucleus	1H
27	Acquired Size	16384
28	Spectral Size	65536

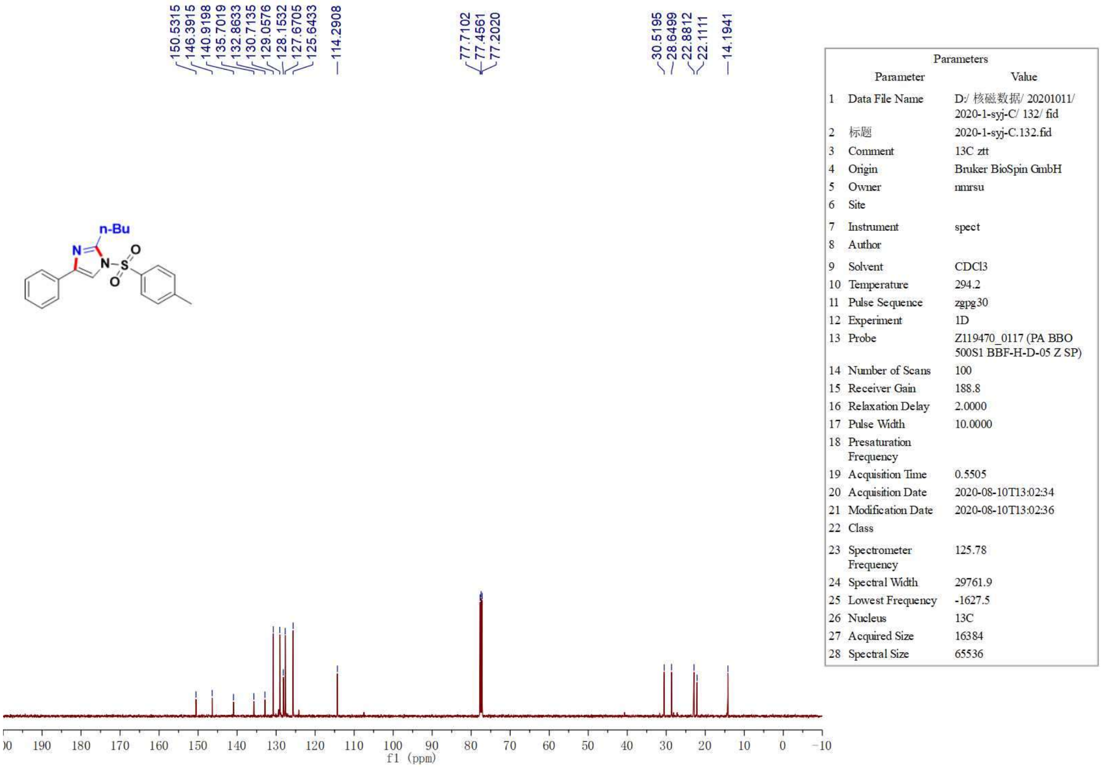

Parameters

	Parameter	Value
1	Data File Name	D:/ 核磁数据/ 20201011/ 2020-1-syj-C/ 132/ fid
2	标题	2020-1-syj-C.132.fid
3	Comment	13C ztt
4	Origin	Bruker BioSpin GmbH
5	Owner	nmrsu
6	Site	
7	Instrument	spect
8	Author	
9	Solvent	CDCl3
10	Temperature	294.2
11	Pulse Sequence	zgpg30
12	Experiment	1D
13	Probe	Z119470_0117 (PA BBO 500S1 BBF-H-D-05 Z SP)
14	Number of Scans	100
15	Receiver Gain	188.8
16	Relaxation Delay	2.0000
17	Pulse Width	10.0000
18	Presaturation Frequency	
19	Acquisition Time	0.5505
20	Acquisition Date	2020-08-10T13:02:34
21	Modification Date	2020-08-10T13:02:36
22	Class	
23	Spectrometer Frequency	125.78
24	Spectral Width	29761.9
25	Lowest Frequency	-1627.5
26	Nucleus	13C
27	Acquired Size	16384
28	Spectral Size	65536

(2,5-Diphenyl-1*H*-pyrrol-3-yl)(phenyl)methanone (5a)

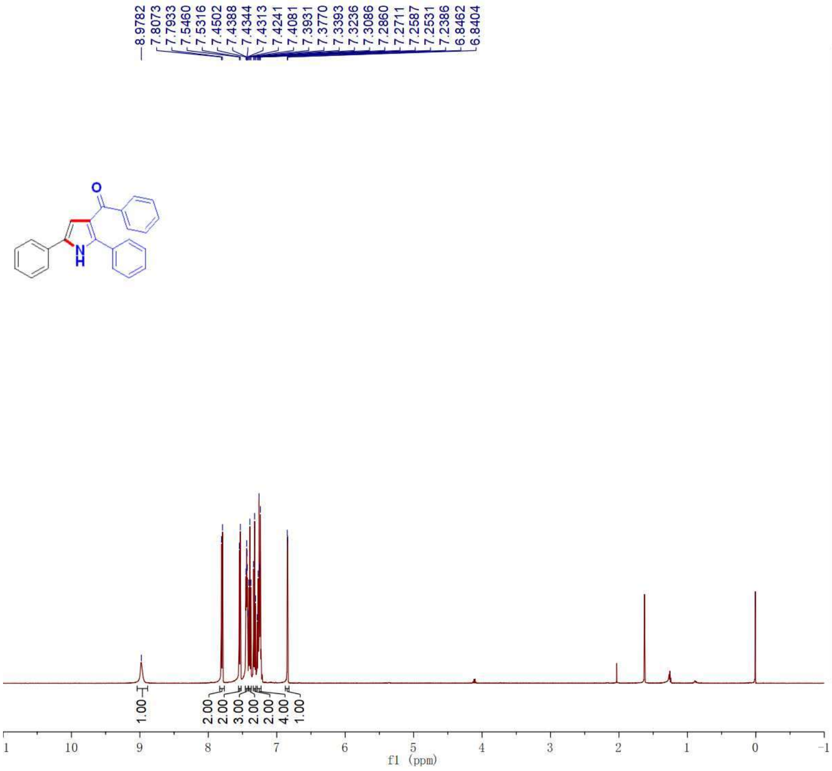

Parameters

	Parameter	Value
1	Data File Name	D:/ 核磁数据/ 500M-NMR-date/ 2019-1-hxw-H/ 332/ fid
2	标题	2019-1-hxw-H.332.fid
3	Comment	1H
4	Origin	Bruker BioSpin GmbH
5	Owner	root
6	Site	
7	Instrument	spect
8	Author	
9	Solvent	CDCl3
10	Temperature	295.2
11	Pulse Sequence	zg30
12	Experiment	1D
13	Probe	Z119470_0117 (PA BBO 500S1 BBF-H-D-05 Z SP)
14	Number of Scans	2
15	Receiver Gain	76.5
16	Relaxation Delay	2.0000
17	Pulse Width	12.0000
18	Presaturation Frequency	
19	Acquisition Time	1.8175
20	Acquisition Date	2019-11-29T11:27:38
21	Modification Date	2019-11-29T11:27:40
22	Class	
23	Spectrometer Frequency	500.16
24	Spectral Width	9014.4
25	Lowest Frequency	-1518.8
26	Nucleus	1H
27	Acquired Size	16384
28	Spectral Size	65536

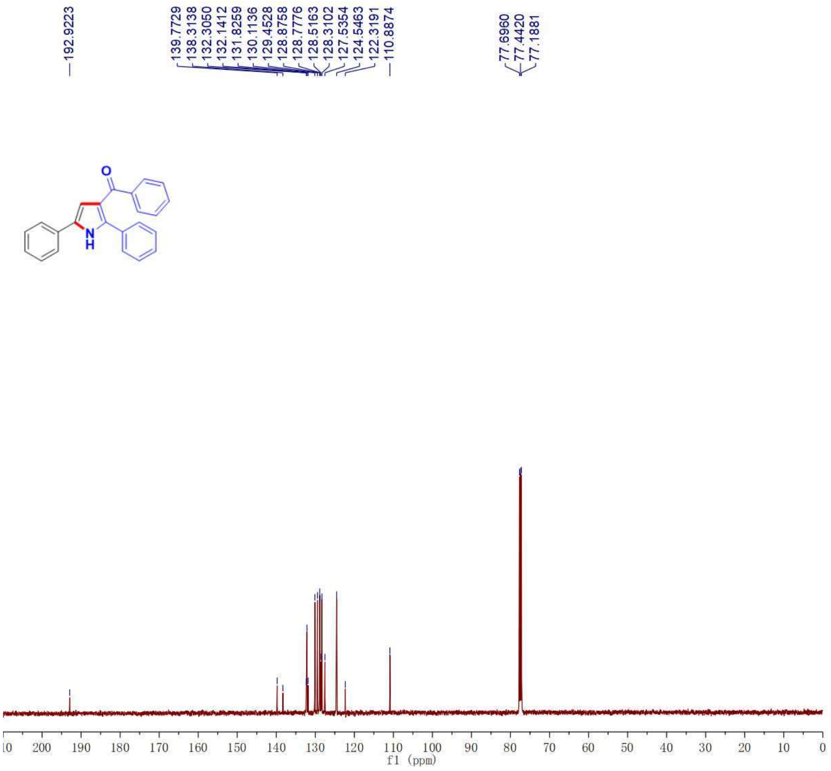

Parameters

	Parameter	Value
1	Data File Name	D:/ 核磁数据/ 500M-NMR-date/ 2019-1-hxw-C/ 255/ fid
2	标题	2019-1-hxw-C.255.fid
3	Comment	13C
4	Origin	Bruker BioSpin GmbH
5	Owner	root
6	Site	
7	Instrument	spect
8	Author	
9	Solvent	CDCl3
10	Temperature	295.2
11	Pulse Sequence	zgpg30
12	Experiment	1D
13	Probe	Z119470_0117 (PA BBO 500S1 BBF-H-D-05 Z SP)
14	Number of Scans	151
15	Receiver Gain	188.8
16	Relaxation Delay	2.0000
17	Pulse Width	10.0000
18	Presaturation Frequency	
19	Acquisition Time	0.5505
20	Acquisition Date	2019-11-29T11:37:06
21	Modification Date	2019-11-29T11:37:08
22	Class	
23	Spectrometer Frequency	125.78
24	Spectral Width	29761.9
25	Lowest Frequency	-1627.5
26	Nucleus	13C
27	Acquired Size	16384
28	Spectral Size	65536

Phenyl(2-phenyl-5-(*p*-tolyl)-1*H*-pyrrol-3-yl)methanone (5b)

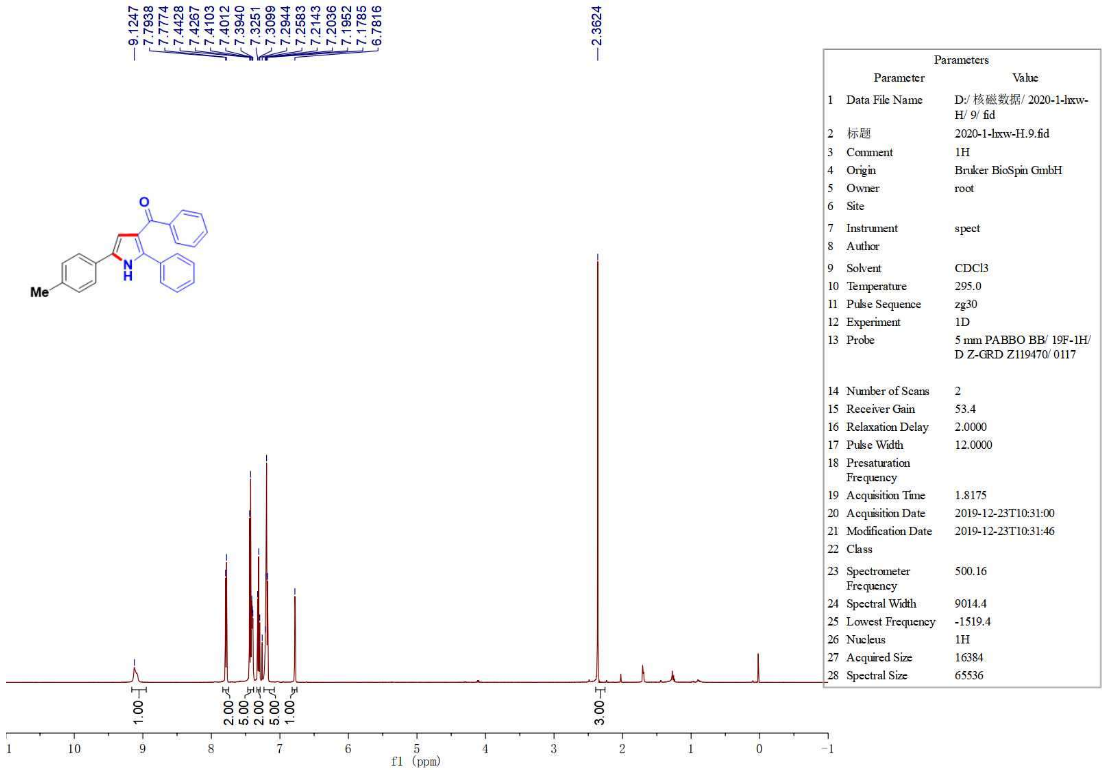

	Parameters	
	Parameter	Value
1	Data File Name	D:/ 核磁数据/ 2020-1-lxw-H/ 9/ fid
2	标题	2020-1-lxw-H.9.fid
3	Comment	1H
4	Origin	Bruker BioSpin GmbH
5	Owner	root
6	Site	
7	Instrument	spect
8	Author	
9	Solvent	CDCl3
10	Temperature	295.0
11	Pulse Sequence	zg30
12	Experiment	1D
13	Probe	5 mm PABBO BB/ 19F-1H/ D Z-GRD Z119470/ 0117
14	Number of Scans	2
15	Receiver Gain	53.4
16	Relaxation Delay	2.0000
17	Pulse Width	12.0000
18	Presaturation Frequency	
19	Acquisition Time	1.8175
20	Acquisition Date	2019-12-23T10:31:00
21	Modification Date	2019-12-23T10:31:46
22	Class	
23	Spectrometer Frequency	500.16
24	Spectral Width	9014.4
25	Lowest Frequency	-1519.4
26	Nucleus	1H
27	Acquired Size	16384
28	Spectral Size	65536

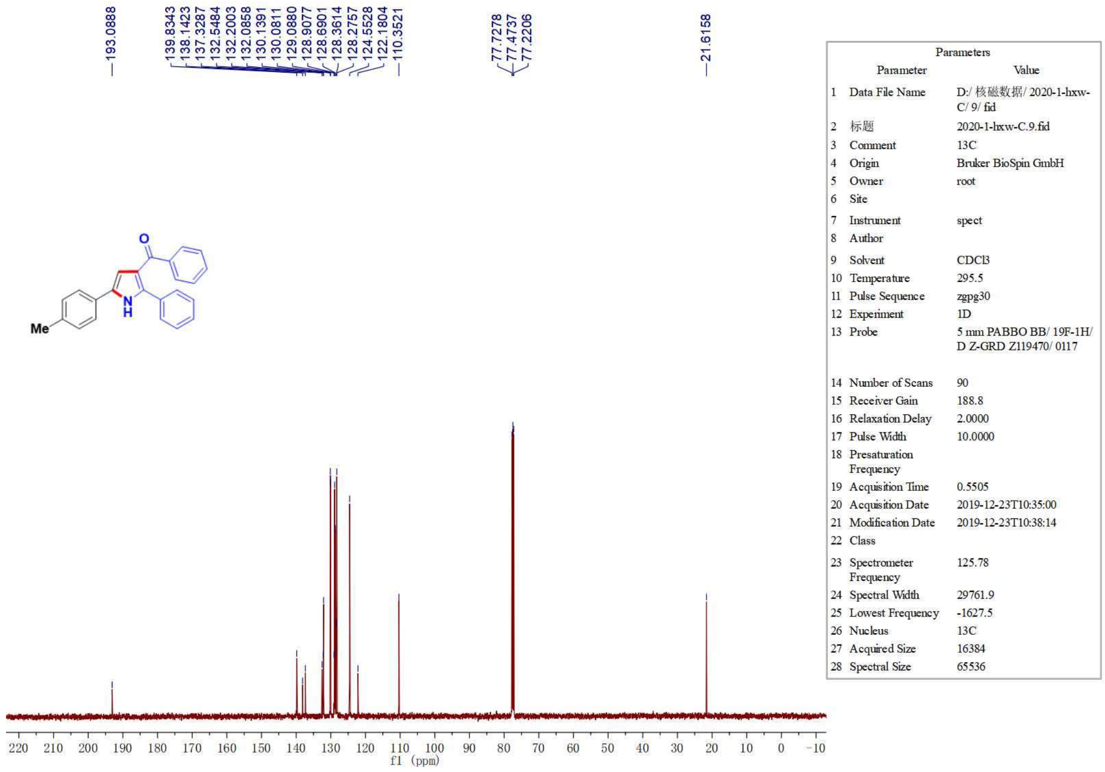

	Parameters	
	Parameter	Value
1	Data File Name	D:/ 核磁数据/ 2020-1-lxw-C/ 9/ fid
2	标题	2020-1-lxw-C.9.fid
3	Comment	13C
4	Origin	Bruker BioSpin GmbH
5	Owner	root
6	Site	
7	Instrument	spect
8	Author	
9	Solvent	CDCl3
10	Temperature	295.5
11	Pulse Sequence	zgpg30
12	Experiment	1D
13	Probe	5 mm PABBO BB/ 19F-1H/ D Z-GRD Z119470/ 0117
14	Number of Scans	90
15	Receiver Gain	188.8
16	Relaxation Delay	2.0000
17	Pulse Width	10.0000
18	Presaturation Frequency	
19	Acquisition Time	0.5505
20	Acquisition Date	2019-12-23T10:35:00
21	Modification Date	2019-12-23T10:38:14
22	Class	
23	Spectrometer Frequency	125.78
24	Spectral Width	29761.9
25	Lowest Frequency	-1627.5
26	Nucleus	13C
27	Acquired Size	16384
28	Spectral Size	65536

(5-(4-Ethylphenyl)-2-phenyl-1*H*-pyrrol-3-yl)(phenyl)methanone (5c)

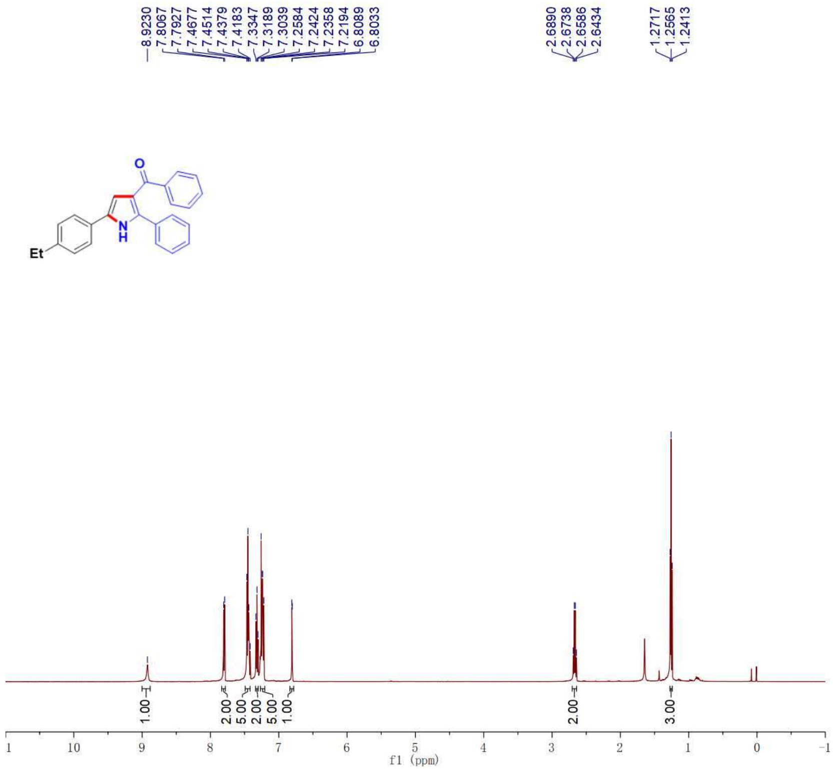

Parameters

	Parameter	Value
1	Data File Name	D:/ 核磁数据/ 20201011/ 2020-1-syj-H/ 313/ fid
2	标题	2020-1-syj-H.313.fid
3	Comment	1H zsw
4	Origin	Bruker BioSpin GmbH
5	Owner	nmrsu
6	Site	
7	Instrument	spect
8	Author	
9	Solvent	CDCl3
10	Temperature	296.5
11	Pulse Sequence	zg30
12	Experiment	1D
13	Probe	Z119470_0117 (PA BBO 500S1 BBF-H-D-05 Z SP)
14	Number of Scans	4
15	Receiver Gain	95.4
16	Relaxation Delay	2.0000
17	Pulse Width	12.0000
18	Presaturation Frequency	
19	Acquisition Time	1.8175
20	Acquisition Date	2020-08-16T09:50:00
21	Modification Date	2020-08-16T09:50:02
22	Class	
23	Spectrometer Frequency	500.16
24	Spectral Width	9014.4
25	Lowest Frequency	-1519.4
26	Nucleus	1H
27	Acquired Size	16384
28	Spectral Size	65536

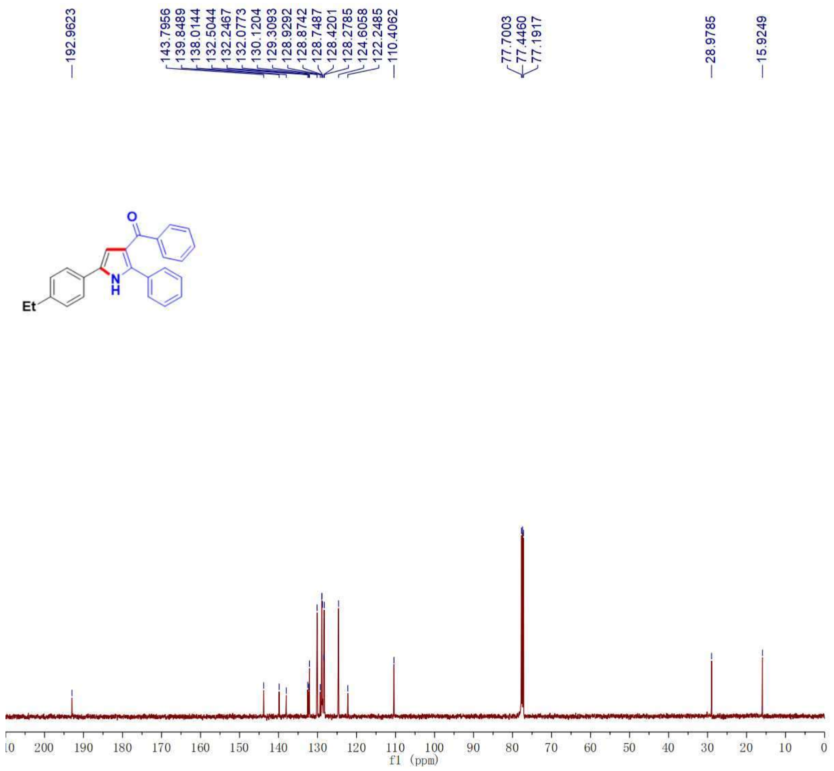

Parameters

	Parameter	Value
1	Data File Name	D:/ 核磁数据/ 20201011/ 2020-1-syj-C/ 145/ fid
2	标题	2020-1-syj-C.145.fid
3	Comment	13C ztt
4	Origin	Bruker BioSpin GmbH
5	Owner	nmrsu
6	Site	
7	Instrument	spect
8	Author	
9	Solvent	CDCl3
10	Temperature	297.1
11	Pulse Sequence	zgpg30
12	Experiment	1D
13	Probe	Z119470_0117 (PA BBO 500S1 BBF-H-D-05 Z SP)
14	Number of Scans	140
15	Receiver Gain	188.8
16	Relaxation Delay	2.0000
17	Pulse Width	10.0000
18	Presaturation Frequency	
19	Acquisition Time	0.5505
20	Acquisition Date	2020-08-18T10:12:04
21	Modification Date	2020-08-18T10:12:06
22	Class	
23	Spectrometer Frequency	125.78
24	Spectral Width	29761.9
25	Lowest Frequency	-1627.5
26	Nucleus	13C
27	Acquired Size	16384
28	Spectral Size	65536

(5-(4-Methoxyphenyl)-2-phenyl-1*H*-pyrrol-3-yl)(phenyl)methanone (5d)

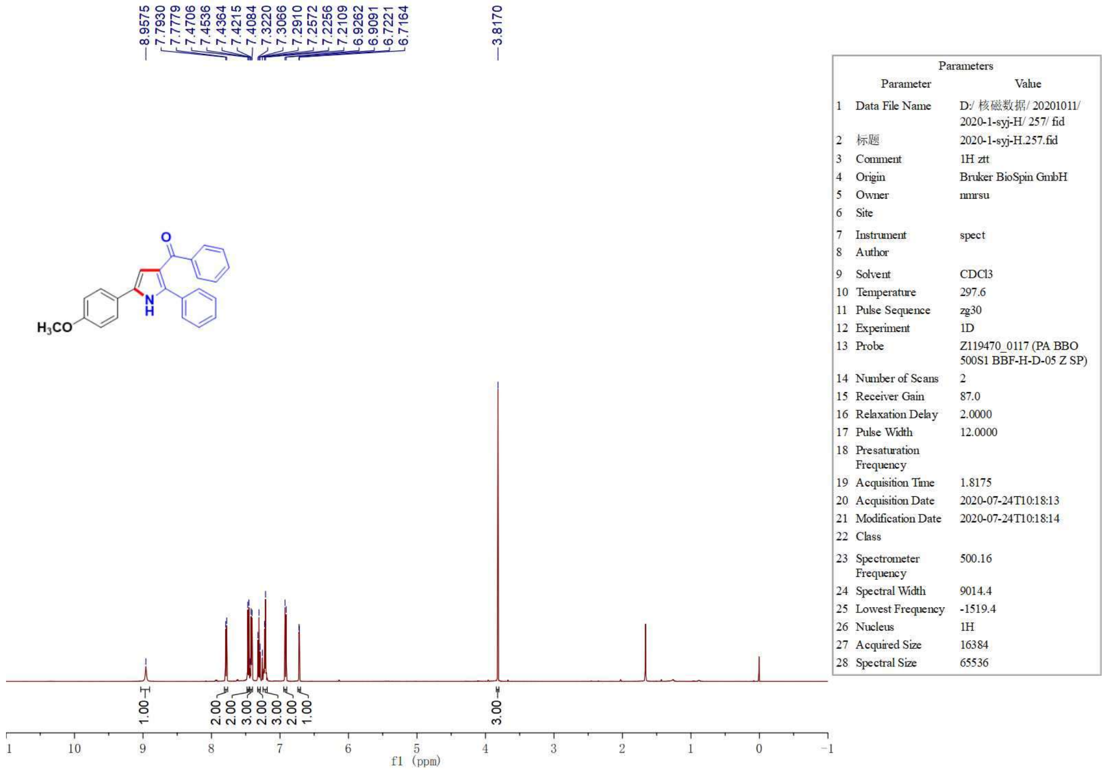

Parameters		
	Parameter	Value
1	Data File Name	D:/ 核磁数据/ 20201011/ 2020-1-syj-H/ 257/ fid
2	标题	2020-1-syj-H.257.fid
3	Comment	1H ztt
4	Origin	Bruker BioSpin GmbH
5	Owner	nmrsu
6	Site	
7	Instrument	spect
8	Author	
9	Solvent	CDCl3
10	Temperature	297.6
11	Pulse Sequence	zg30
12	Experiment	1D
13	Probe	Z119470_0117 (PA BBO 500S1 BBF-H-D-05 Z SP)
14	Number of Scans	2
15	Receiver Gain	87.0
16	Relaxation Delay	2.0000
17	Pulse Width	12.0000
18	Presaturation Frequency	
19	Acquisition Time	1.8175
20	Acquisition Date	2020-07-24T10:18:13
21	Modification Date	2020-07-24T10:18:14
22	Class	
23	Spectrometer Frequency	500.16
24	Spectral Width	9014.4
25	Lowest Frequency	-1519.4
26	Nucleus	1H
27	Acquired Size	16384
28	Spectral Size	65536

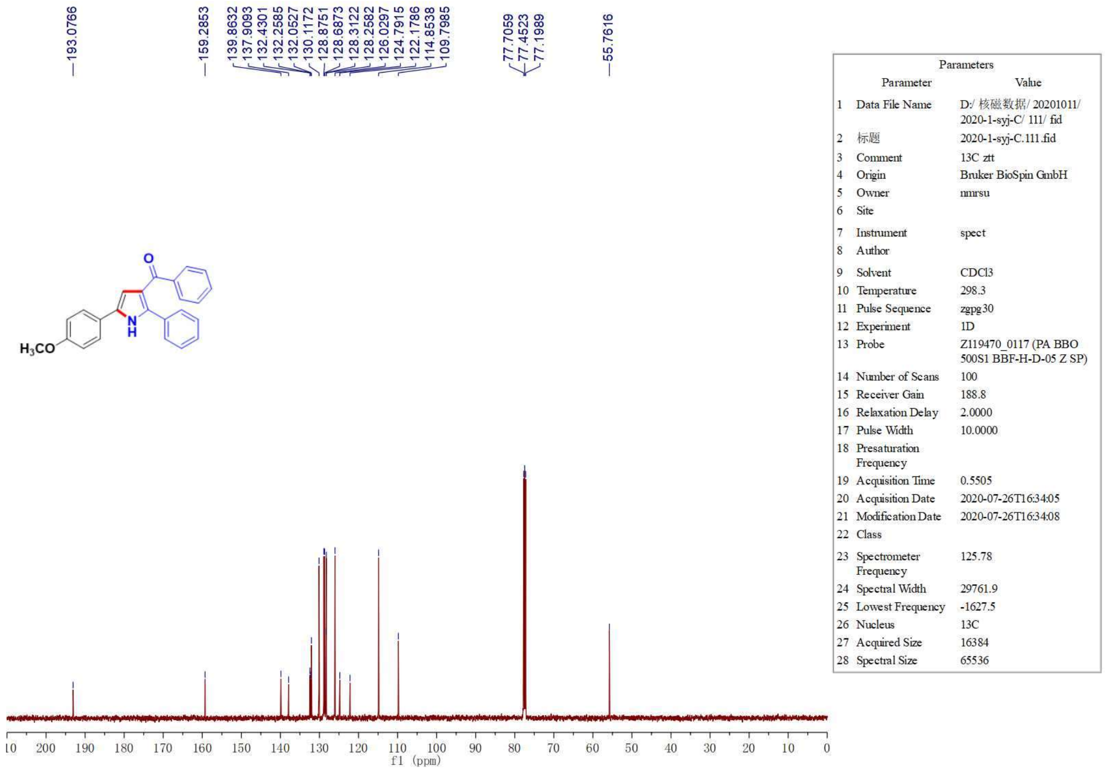

Parameters		
	Parameter	Value
1	Data File Name	D:/ 核磁数据/ 20201011/ 2020-1-syj-C/ 111/ fid
2	标题	2020-1-syj-C.111.fid
3	Comment	13C ztt
4	Origin	Bruker BioSpin GmbH
5	Owner	nmrsu
6	Site	
7	Instrument	spect
8	Author	
9	Solvent	CDCl3
10	Temperature	298.3
11	Pulse Sequence	zgpg30
12	Experiment	1D
13	Probe	Z119470_0117 (PA BBO 500S1 BBF-H-D-05 Z SP)
14	Number of Scans	100
15	Receiver Gain	188.8
16	Relaxation Delay	2.0000
17	Pulse Width	10.0000
18	Presaturation Frequency	
19	Acquisition Time	0.5505
20	Acquisition Date	2020-07-26T16:34:05
21	Modification Date	2020-07-26T16:34:08
22	Class	
23	Spectrometer Frequency	125.78
24	Spectral Width	29761.9
25	Lowest Frequency	-1627.5
26	Nucleus	13C
27	Acquired Size	16384
28	Spectral Size	65536

(5-(4-Fluorophenyl)-2-phenyl-1*H*-pyrrol-3-yl)(phenyl)methanone (5e)

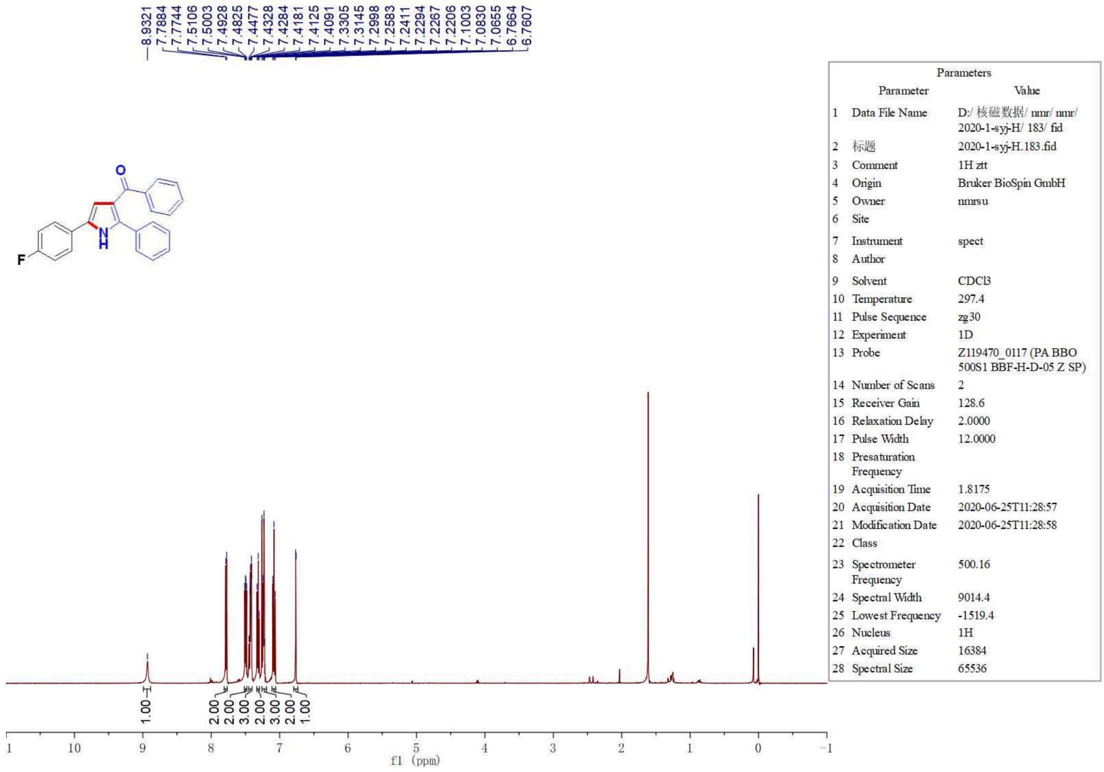

Parameters		
	Parameter	Value
1	Data File Name	D:/ 核磁数据/ nmr/ nmr/ 2020-1-syj-H/ 183/ fid
2	标题	2020-1-syj-H.183.fid
3	Comment	1H ztt
4	Origin	Bruker BioSpin GmbH
5	Owner	nmrsu
6	Site	
7	Instrument	spect
8	Author	
9	Solvent	CDCl3
10	Temperature	297.4
11	Pulse Sequence	zg30
12	Experiment	1D
13	Probe	Z119470_0117 (PA BBO 500S1 BBF-H-D-05 Z SP)
14	Number of Scans	2
15	Receiver Gain	128.6
16	Relaxation Delay	2.0000
17	Pulse Width	12.0000
18	Presaturation Frequency	
19	Acquisition Time	1.8175
20	Acquisition Date	2020-06-25T11:28:57
21	Modification Date	2020-06-25T11:28:58
22	Class	
23	Spectrometer Frequency	500.16
24	Spectral Width	9014.4
25	Lowest Frequency	-1519.4
26	Nucleus	1H
27	Acquired Size	16384
28	Spectral Size	65536

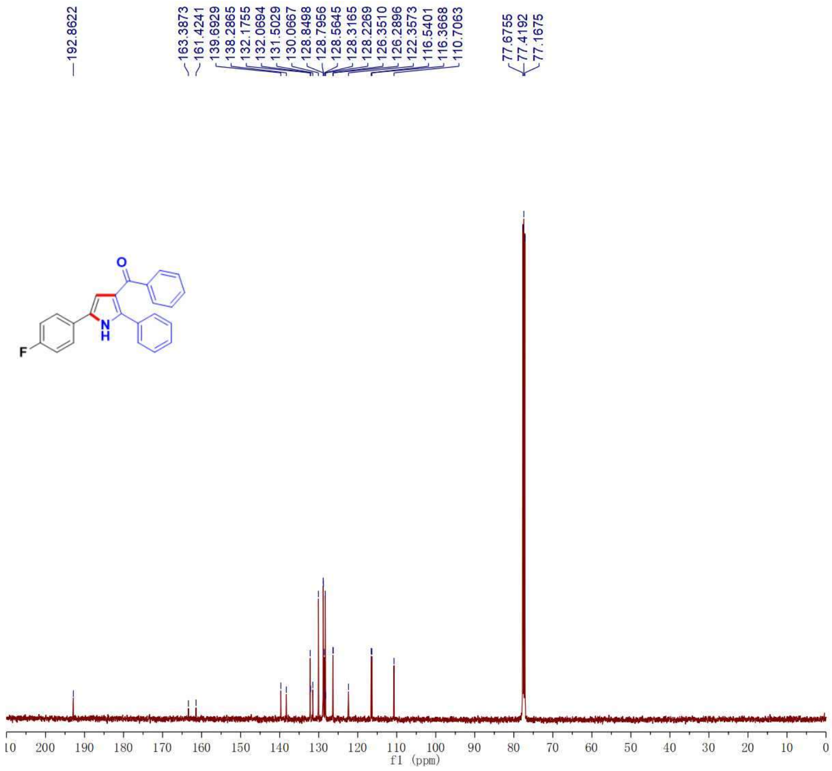

Parameters		
	Parameter	Value
1	Data File Name	D:/ 核磁数据/ nmr/ nmr/ 2020-1-syj-C/ 78/ fid
2	标题	2020-1-syj-C.78.fid
3	Comment	13C ztt
4	Origin	Bruker BioSpin GmbH
5	Owner	nmrsu
6	Site	
7	Instrument	spect
8	Author	
9	Solvent	CDCl3
10	Temperature	298.1
11	Pulse Sequence	zgpg30
12	Experiment	1D
13	Probe	Z119470_0117 (PA BBO 500S1 BBF-H-D-05 Z SP)
14	Number of Scans	500
15	Receiver Gain	188.8
16	Relaxation Delay	2.0000
17	Pulse Width	10.0000
18	Presaturation Frequency	
19	Acquisition Time	0.5505
20	Acquisition Date	2020-06-26T08:18:00
21	Modification Date	2020-06-26T08:18:02
22	Class	
23	Spectrometer Frequency	125.78
24	Spectral Width	29761.9
25	Lowest Frequency	-1627.5
26	Nucleus	13C
27	Acquired Size	16384
28	Spectral Size	65536

(5-(4-Chlorophenyl)-2-phenyl-1*H*-pyrrol-3-yl)(phenyl)methanone (5f)

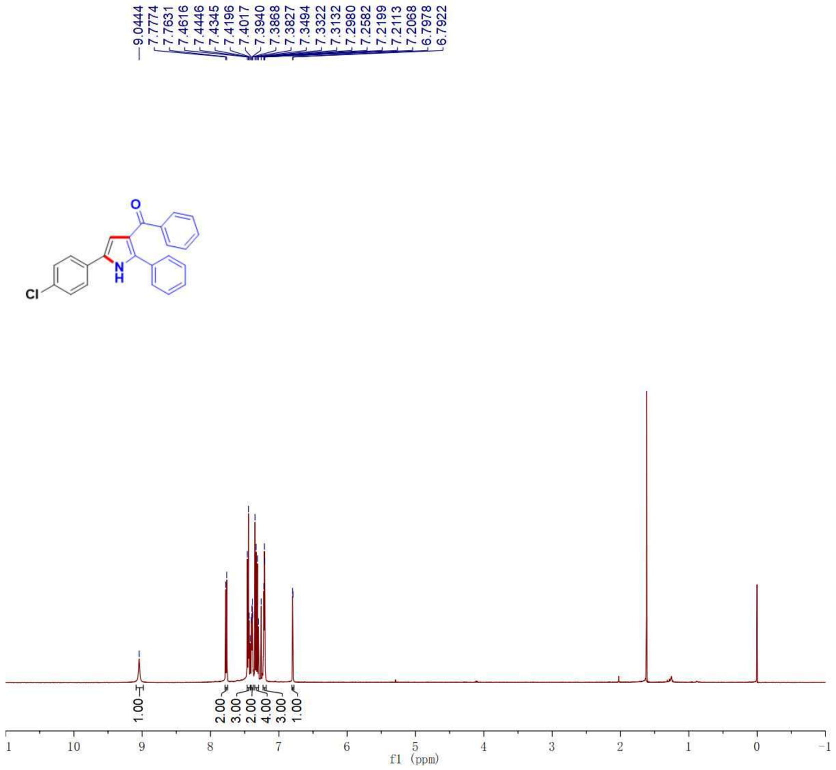

	Parameter	Value
1	Data File Name	D:/ 核磁数据/ 20201011/ 2020-1-syj-H/ 267/ fid
2	标题	2020-1-syj-H.267.fid
3	Comment	1H ztt
4	Origin	Bruker BioSpin GmbH
5	Owner	nmrsu
6	Site	
7	Instrument	spect
8	Author	
9	Solvent	CDCl3
10	Temperature	297.4
11	Pulse Sequence	zg30
12	Experiment	1D
13	Probe	Z119470_0117 (PA BBO 500S1 BBF-H-D-05 Z SP)
14	Number of Scans	2
15	Receiver Gain	114.1
16	Relaxation Delay	2.0000
17	Pulse Width	12.0000
18	Presaturation Frequency	
19	Acquisition Time	1.8175
20	Acquisition Date	2020-07-26T11:37:31
21	Modification Date	2020-07-26T11:37:32
22	Class	
23	Spectrometer Frequency	500.16
24	Spectral Width	9014.4
25	Lowest Frequency	-1519.4
26	Nucleus	1H
27	Acquired Size	16384
28	Spectral Size	65536

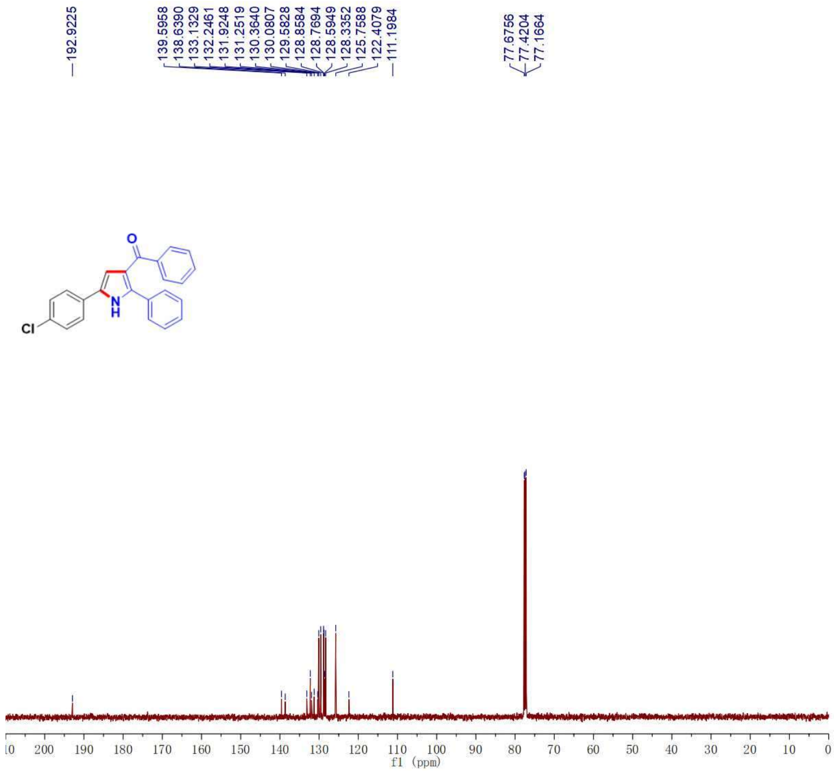

	Parameter	Value
1	Data File Name	D:/ 核磁数据/ 20201011/ 2020-1-syj-C/ 114/ fid
2	标题	2020-1-syj-C.114.fid
3	Comment	13C ztt
4	Origin	Bruker BioSpin GmbH
5	Owner	nmrsu
6	Site	
7	Instrument	spect
8	Author	
9	Solvent	CDCl3
10	Temperature	298.3
11	Pulse Sequence	zgpg30
12	Experiment	1D
13	Probe	Z119470_0117 (PA BBO 500S1 BBF-H-D-05 Z SP)
14	Number of Scans	83
15	Receiver Gain	188.8
16	Relaxation Delay	2.0000
17	Pulse Width	10.0000
18	Presaturation Frequency	
19	Acquisition Time	0.5505
20	Acquisition Date	2020-07-26T16:59:52
21	Modification Date	2020-07-26T16:59:54
22	Class	
23	Spectrometer Frequency	125.78
24	Spectral Width	29761.9
25	Lowest Frequency	-1627.5
26	Nucleus	13C
27	Acquired Size	16384
28	Spectral Size	65536

(5-(4-Bromophenyl)-2-phenyl-1*H*-pyrrol-3-yl)(phenyl)methanone (5g)

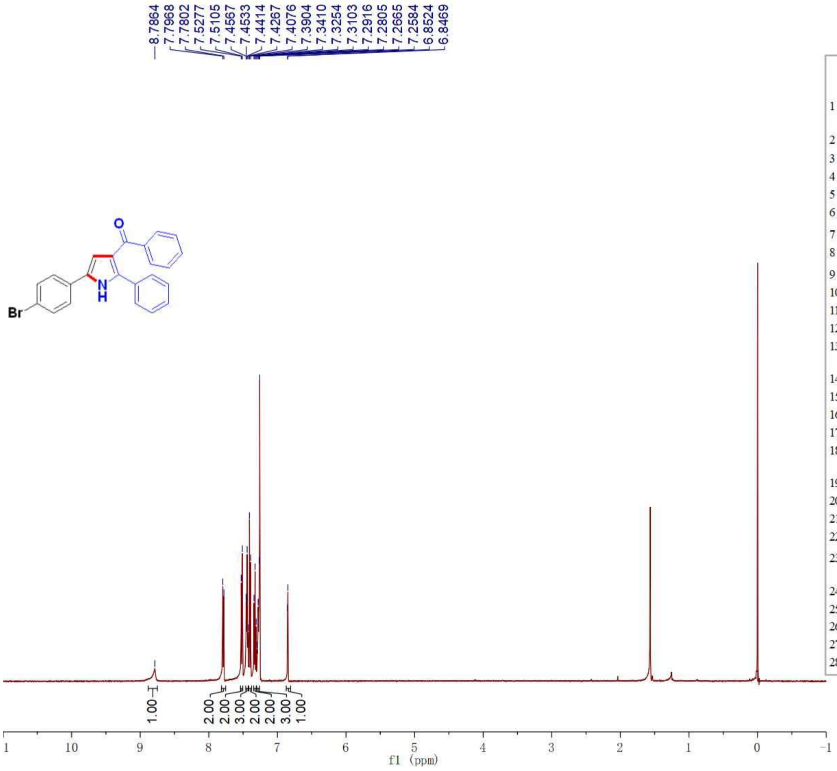

	Parameter	Value
1	Data File Name	D:/ 核磁数据/ nmr/ nmr/ 2020-1-syj-H/ 127/ fid
2	标题	2020-1-syj-H.127.fid
3	Comment	1H ztt
4	Origin	Bruker BioSpin GmbH
5	Owner	nmrsu
6	Site	
7	Instrument	spect
8	Author	
9	Solvent	CDCl3
10	Temperature	297.2
11	Pulse Sequence	zg30
12	Experiment	1D
13	Probe	Z119470_0117 (PA BBO 500S1 BBF-H-D-05 Z SP)
14	Number of Scans	2
15	Receiver Gain	188.8
16	Relaxation Delay	2.0000
17	Pulse Width	12.0000
18	Presaturation Frequency	
19	Acquisition Time	1.8175
20	Acquisition Date	2020-06-04T12:55:35
21	Modification Date	2020-06-04T12:55:36
22	Class	
23	Spectrometer Frequency	500.16
24	Spectral Width	9014.4
25	Lowest Frequency	-1519.4
26	Nucleus	1H
27	Acquired Size	16384
28	Spectral Size	65536

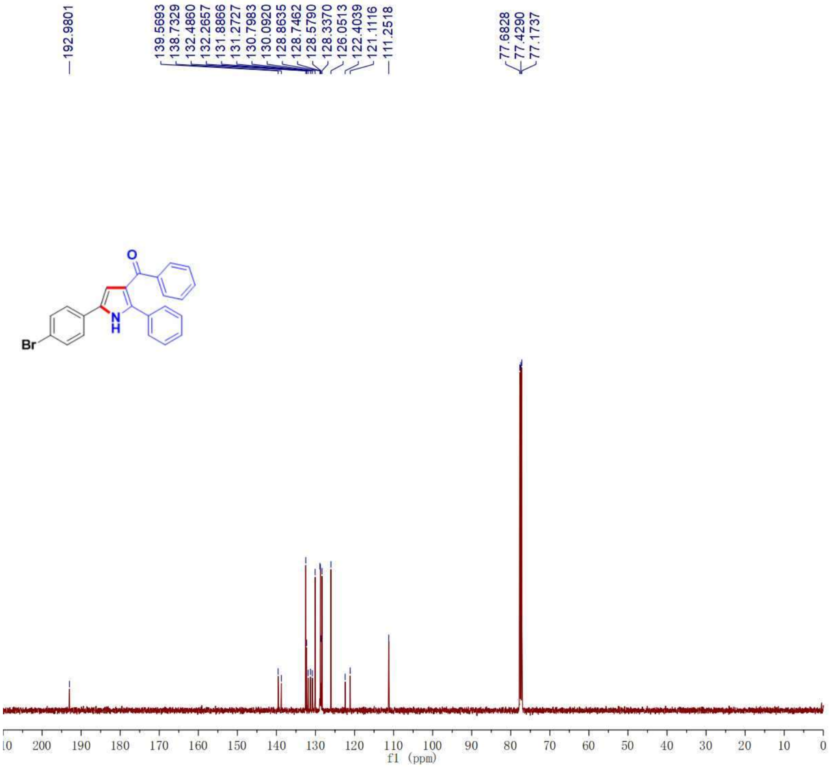

	Parameter	Value
1	Data File Name	D:/ 核磁数据/ nmr/ nmr/ 2020-1-syj-C/ 51/ fid
2	标题	2020-1-syj-C.51.fid
3	Comment	13C ztt
4	Origin	Bruker BioSpin GmbH
5	Owner	nmrsu
6	Site	
7	Instrument	spect
8	Author	
9	Solvent	CDCl3
10	Temperature	298.0
11	Pulse Sequence	zgpg30
12	Experiment	1D
13	Probe	Z119470_0117 (PA BBO 500S1 BBF-H-D-05 Z SP)
14	Number of Scans	201
15	Receiver Gain	188.8
16	Relaxation Delay	2.0000
17	Pulse Width	10.0000
18	Presaturation Frequency	
19	Acquisition Time	0.5505
20	Acquisition Date	2020-06-05T12:23:03
21	Modification Date	2020-06-05T12:23:06
22	Class	
23	Spectrometer Frequency	125.78
24	Spectral Width	29761.9
25	Lowest Frequency	-1627.5
26	Nucleus	13C
27	Acquired Size	16384
28	Spectral Size	65536

(5-(4-(*tert*-Butyl)phenyl)-2-phenyl-1*H*-pyrrol-3-yl)(phenyl)methanone (**5h**)

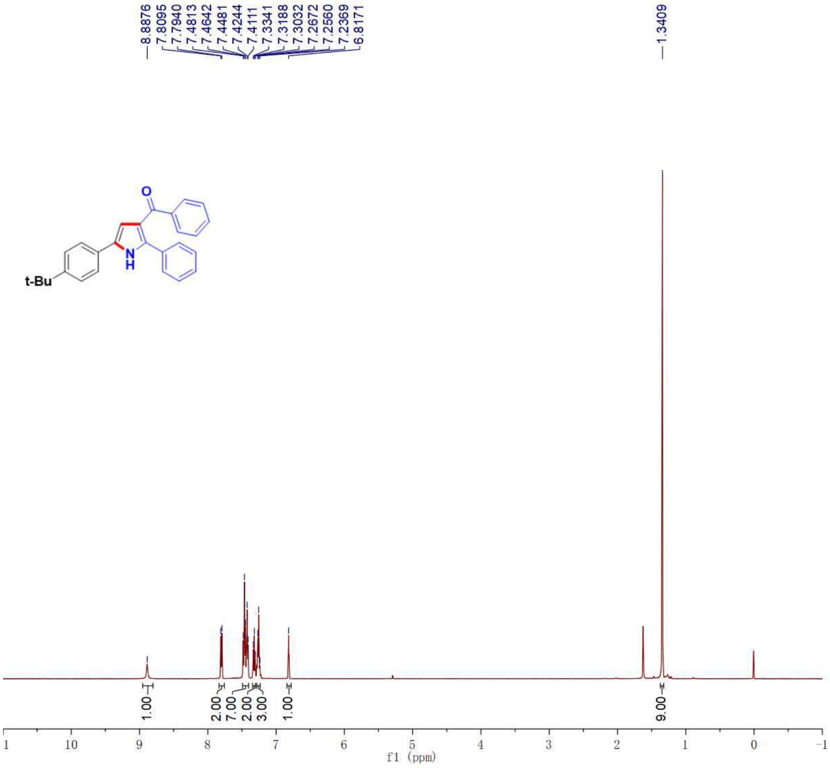

Parameter	Value
1 Data File Name	D:/ 核磁数据/ 20201011/ 2020-1-syj-H/ 268/ fid
2 标题	2020-1-syj-H.268.fid
3 Comment	1H ztt
4 Origin	Bruker BioSpin GmbH
5 Owner	nmrsu
6 Site	
7 Instrument	spect
8 Author	
9 Solvent	CDCl3
10 Temperature	297.4
11 Pulse Sequence	zg30
12 Experiment	1D
13 Probe	Z119470_0117 (PA BBO 500S1 BBF-H-D-05 Z SP)
14 Number of Scans	2
15 Receiver Gain	87.0
16 Relaxation Delay	2.0000
17 Pulse Width	12.0000
18 Presaturation Frequency	
19 Acquisition Time	1.8175
20 Acquisition Date	2020-07-26T11:42:58
21 Modification Date	2020-07-26T11:43:00
22 Class	
23 Spectrometer Frequency	500.16
24 Spectral Width	9014.4
25 Lowest Frequency	-1519.4
26 Nucleus	1H
27 Acquired Size	16384
28 Spectral Size	65536

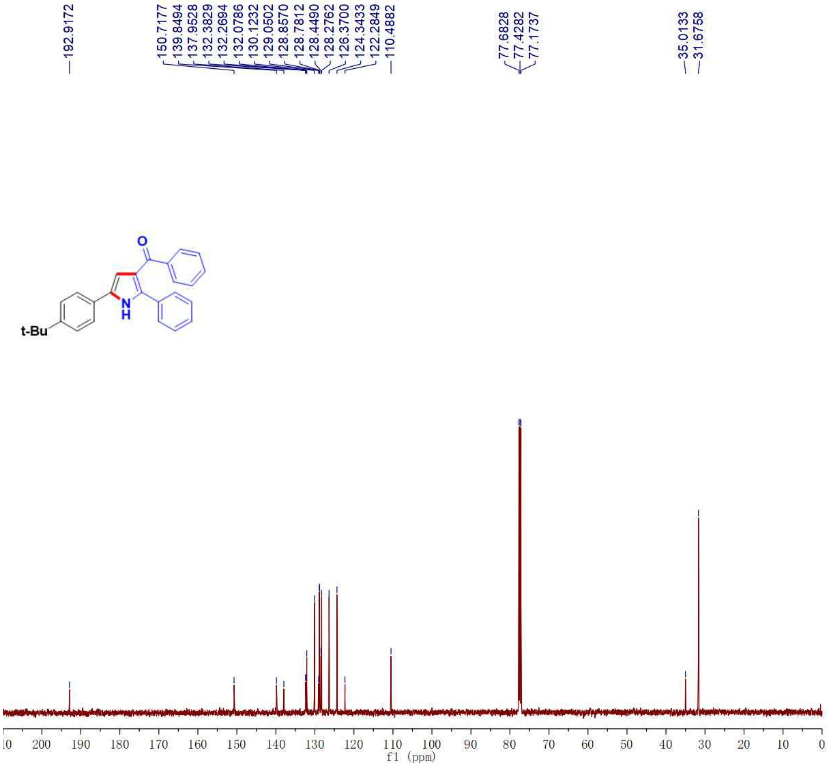

Parameter	Value
1 Data File Name	D:/ 核磁数据/ 20201011/ 2020-1-syj-C/ 112/ fid
2 标题	2020-1-syj-C.112.fid
3 Comment	13C ztt
4 Origin	Bruker BioSpin GmbH
5 Owner	nmrsu
6 Site	
7 Instrument	spect
8 Author	
9 Solvent	CDCl3
10 Temperature	298.2
11 Pulse Sequence	zgpg30
12 Experiment	1D
13 Probe	Z119470_0117 (PA BBO 500S1 BBF-H-D-05 Z SP)
14 Number of Scans	123
15 Receiver Gain	188.8
16 Relaxation Delay	2.0000
17 Pulse Width	10.0000
18 Presaturation Frequency	
19 Acquisition Time	0.5505
20 Acquisition Date	2020-07-26T16:42:14
21 Modification Date	2020-07-26T16:42:16
22 Class	
23 Spectrometer Frequency	125.78
24 Spectral Width	29761.9
25 Lowest Frequency	-1627.5
26 Nucleus	13C
27 Acquired Size	16384
28 Spectral Size	65536

Phenyl(2-phenyl-5-(4'-propyl-[1,1'-biphenyl]-4-yl)-1*H*-pyrrol-3-yl)methanone (5i)

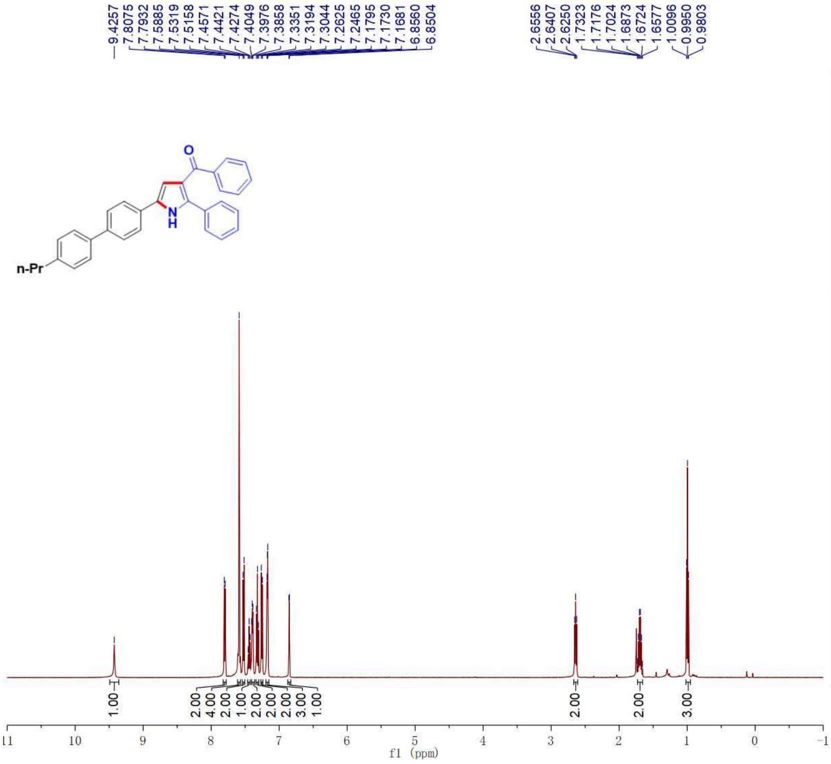

Parameters		
	Parameter	Value
1	Data File Name	D:/ 核磁数据/ 20201011/ 2020-1-syj-H/ 314/ fid
2	标题	2020-1-syj-H.314.fid
3	Comment	1H ztt
4	Origin	Bruker BioSpin GmbH
5	Owner	nmrsu
6	Site	
7	Instrument	spect
8	Author	
9	Solvent	CDCl3
10	Temperature	296.5
11	Pulse Sequence	zg30
12	Experiment	1D
13	Probe	Z119470_0117 (PA BBO 500S1 BBF-H-D-05 Z SP)
14	Number of Scans	4
15	Receiver Gain	27.1
16	Relaxation Delay	2.0000
17	Pulse Width	12.0000
18	Presaturation Frequency	
19	Acquisition Time	1.8175
20	Acquisition Date	2020-08-16T09:54:42
21	Modification Date	2020-08-16T09:54:44
22	Class	
23	Spectrometer Frequency	500.16
24	Spectral Width	9014.4
25	Lowest Frequency	-1519.4
26	Nucleus	1H
27	Acquired Size	16384
28	Spectral Size	65536

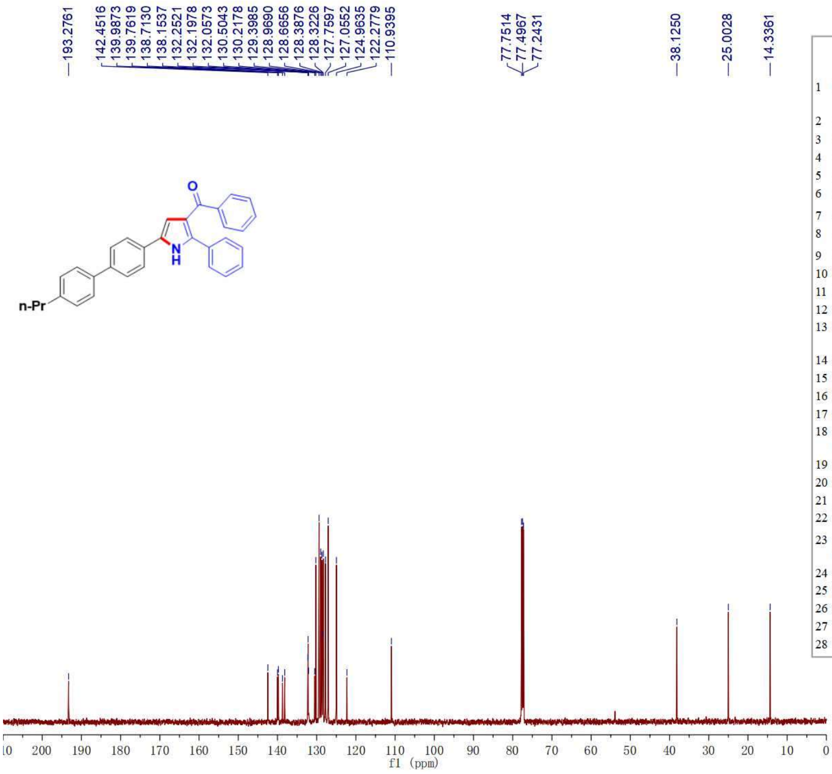

Parameters		
	Parameter	Value
1	Data File Name	D:/ 核磁数据/ 2020-syj-HC/ 2020-1-syj-C/ 358/ fid
2	标题	2020-1-syj-C.358.fid
3	Comment	13C ztt
4	Origin	Bruker BioSpin GmbH
5	Owner	nmrsu
6	Site	
7	Instrument	spect
8	Author	
9	Solvent	CDCl3
10	Temperature	295.4
11	Pulse Sequence	zgpg30
12	Experiment	1D
13	Probe	Z119470_0117 (PA BBO 500S1 BBF-H-D-05 Z SP)
14	Number of Scans	100
15	Receiver Gain	188.8
16	Relaxation Delay	2.0000
17	Pulse Width	10.0000
18	Presaturation Frequency	
19	Acquisition Time	0.5505
20	Acquisition Date	2020-12-24T19:25:57
21	Modification Date	2020-12-24T19:26:00
22	Class	
23	Spectrometer Frequency	125.78
24	Spectral Width	29761.9
25	Lowest Frequency	-1627.5
26	Nucleus	13C
27	Acquired Size	16384
28	Spectral Size	65536

Phenyl(2-phenyl-5-(*m*-tolyl)-1*H*-pyrrol-3-yl)methanone (5j)

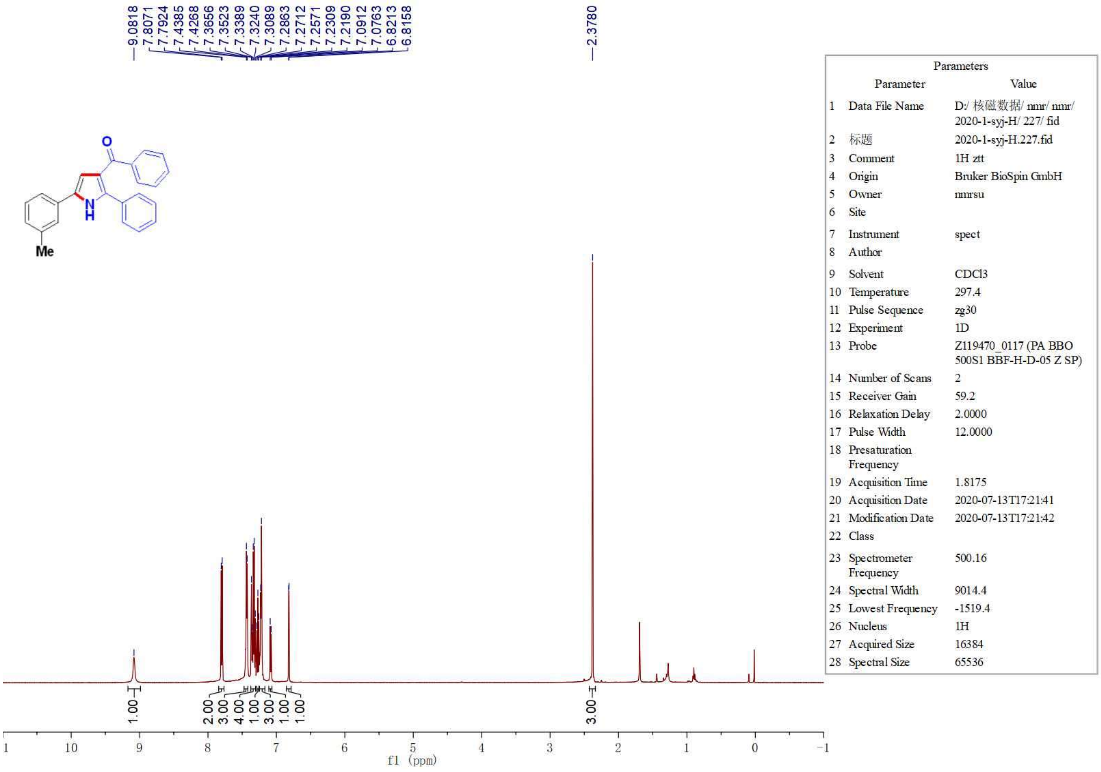

Parameter	Value
1 Data File Name	D:/ 核磁数据/ nmr/ nmr/ 2020-1-syj-H/ 227/ fid
2 标题	2020-1-syj-H.227.fid
3 Comment	1H ztt
4 Origin	Bruker BioSpin GmbH
5 Owner	nmrsu
6 Site	
7 Instrument	spect
8 Author	
9 Solvent	CDCl3
10 Temperature	297.4
11 Pulse Sequence	zg30
12 Experiment	1D
13 Probe	Z119470_0117 (PA BBO 500S1 BBF-H-D-05 Z SP)
14 Number of Scans	2
15 Receiver Gain	59.2
16 Relaxation Delay	2.0000
17 Pulse Width	12.0000
18 Presaturation Frequency	
19 Acquisition Time	1.8175
20 Acquisition Date	2020-07-13T17:21:41
21 Modification Date	2020-07-13T17:21:42
22 Class	
23 Spectrometer Frequency	500.16
24 Spectral Width	9014.4
25 Lowest Frequency	-1519.4
26 Nucleus	1H
27 Acquired Size	16384
28 Spectral Size	65536

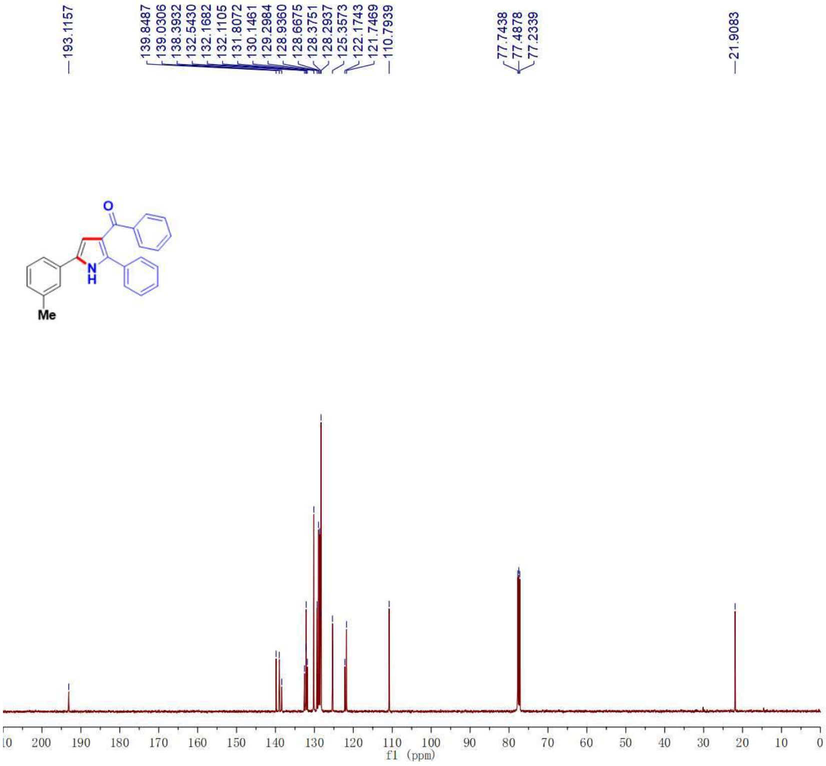

Parameter	Value
1 Data File Name	D:/ 核磁数据/ nmr/ nmr/ 2020-1-syj-C/ 90/ fid
2 标题	2020-1-syj-C.90.fid
3 Comment	13C ztt
4 Origin	Bruker BioSpin GmbH
5 Owner	nmrsu
6 Site	
7 Instrument	spect
8 Author	
9 Solvent	CDCl3
10 Temperature	298.4
11 Pulse Sequence	zgpg30
12 Experiment	1D
13 Probe	Z119470_0117 (PA BBO 500S1 BBF-H-D-05 Z SP)
14 Number of Scans	330
15 Receiver Gain	188.8
16 Relaxation Delay	2.0000
17 Pulse Width	10.0000
18 Presaturation Frequency	
19 Acquisition Time	0.5505
20 Acquisition Date	2020-07-14T10:29:55
21 Modification Date	2020-07-14T10:29:58
22 Class	
23 Spectrometer Frequency	125.78
24 Spectral Width	29761.9
25 Lowest Frequency	-1627.5
26 Nucleus	13C
27 Acquired Size	16384
28 Spectral Size	65536

(5-(3-Chlorophenyl)-2-phenyl-1*H*-pyrrol-3-yl)(phenyl)methanone (5k)

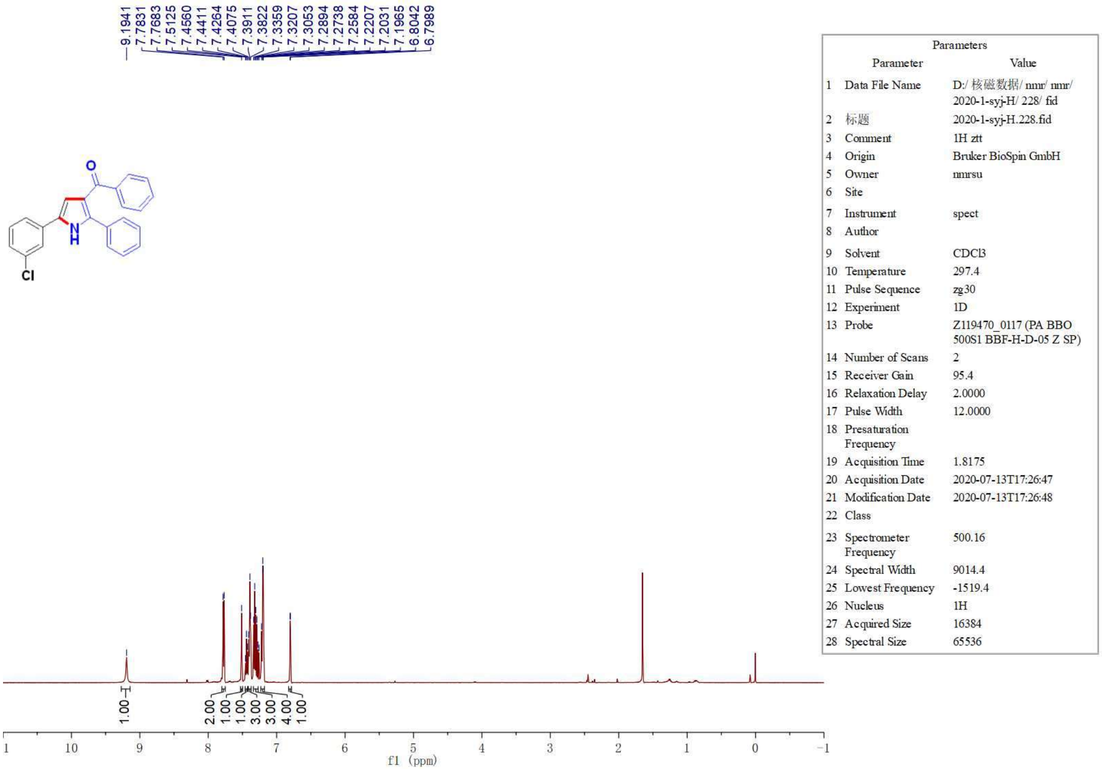

	Parameter	Value
1	Data File Name	D:/ 核磁数据/ nmr/ nmr/ 2020-1-syj-H/ 228/ fid
2	标题	2020-1-syj-H.228.fid
3	Comment	1H ztt
4	Origin	Bruker BioSpin GmbH
5	Owner	nmrsu
6	Site	
7	Instrument	spect
8	Author	
9	Solvent	CDCl3
10	Temperature	297.4
11	Pulse Sequence	zg30
12	Experiment	1D
13	Probe	Z119470_0117 (PA BBO 500S1 BBF-H-D-05 Z SP)
14	Number of Scans	2
15	Receiver Gain	95.4
16	Relaxation Delay	2.0000
17	Pulse Width	12.0000
18	Presaturation Frequency	
19	Acquisition Time	1.8175
20	Acquisition Date	2020-07-13T17:26:47
21	Modification Date	2020-07-13T17:26:48
22	Class	
23	Spectrometer Frequency	500.16
24	Spectral Width	9014.4
25	Lowest Frequency	-1519.4
26	Nucleus	1H
27	Acquired Size	16384
28	Spectral Size	65536

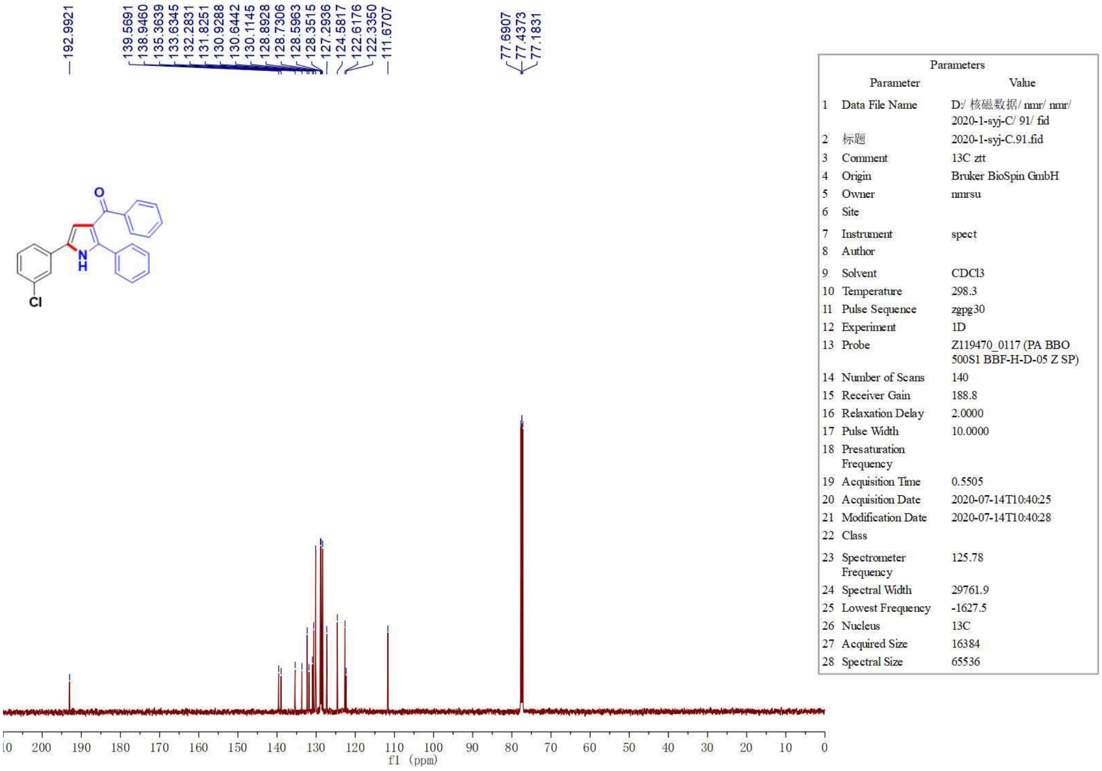

	Parameter	Value
1	Data File Name	D:/ 核磁数据/ nmr/ nmr/ 2020-1-syj-C/ 91/ fid
2	标题	2020-1-syj-C.91.fid
3	Comment	13C ztt
4	Origin	Bruker BioSpin GmbH
5	Owner	nmrsu
6	Site	
7	Instrument	spect
8	Author	
9	Solvent	CDCl3
10	Temperature	298.3
11	Pulse Sequence	zgpg30
12	Experiment	1D
13	Probe	Z119470_0117 (PA BBO 500S1 BBF-H-D-05 Z SP)
14	Number of Scans	140
15	Receiver Gain	188.8
16	Relaxation Delay	2.0000
17	Pulse Width	10.0000
18	Presaturation Frequency	
19	Acquisition Time	0.5505
20	Acquisition Date	2020-07-14T10:40:25
21	Modification Date	2020-07-14T10:40:28
22	Class	
23	Spectrometer Frequency	125.78
24	Spectral Width	29761.9
25	Lowest Frequency	-1627.5
26	Nucleus	13C
27	Acquired Size	16384
28	Spectral Size	65536

(5-(3-Bromophenyl)-2-phenyl-1*H*-pyrrol-3-yl)(phenyl)methanone (5l)

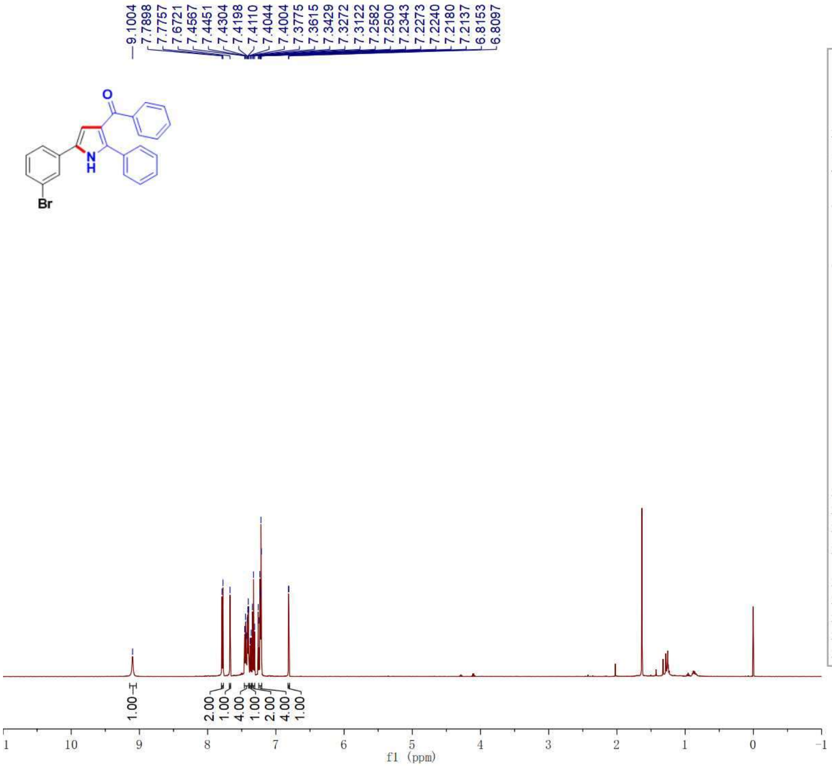

Parameters

	Parameter	Value
1	Data File Name	D:/ 核磁数据/ 20201011/ 2020-1-syj-H/ 266/ fid
2	标题	2020-1-syj-H.266.fid
3	Comment	1H ztt
4	Origin	Bruker BioSpin GmbH
5	Owner	nmrsu
6	Site	
7	Instrument	spect
8	Author	
9	Solvent	CDCl3
10	Temperature	297.4
11	Pulse Sequence	zg30
12	Experiment	1D
13	Probe	Z119470_0117 (PA BBO 500S1 BBF-H-D-05 Z SP)
14	Number of Scans	2
15	Receiver Gain	114.1
16	Relaxation Delay	2.0000
17	Pulse Width	12.0000
18	Presaturation Frequency	
19	Acquisition Time	1.8175
20	Acquisition Date	2020-07-26T11:32:32
21	Modification Date	2020-07-26T11:32:34
22	Class	
23	Spectrometer Frequency	500.16
24	Spectral Width	9014.4
25	Lowest Frequency	-1519.4
26	Nucleus	1H
27	Acquired Size	16384
28	Spectral Size	65536

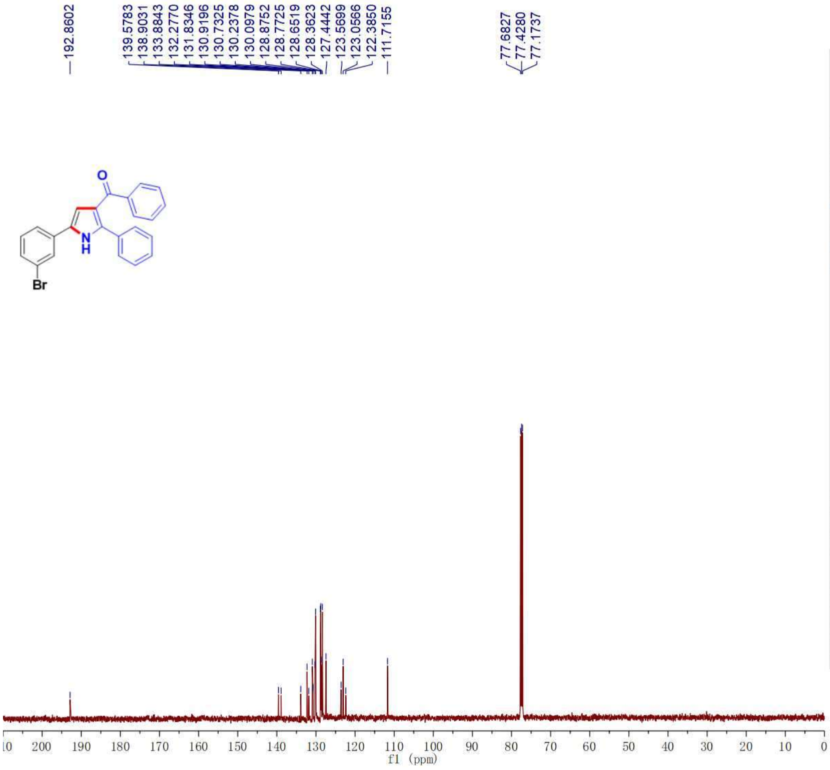

Parameters

	Parameter	Value
1	Data File Name	D:/ 核磁数据/ 20201011/ 2020-1-syj-C/ 113/ fid
2	标题	2020-1-syj-C.113.fid
3	Comment	13C ztt
4	Origin	Bruker BioSpin GmbH
5	Owner	nmrsu
6	Site	
7	Instrument	spect
8	Author	
9	Solvent	CDCl3
10	Temperature	298.3
11	Pulse Sequence	zgpg30
12	Experiment	1D
13	Probe	Z119470_0117 (PA BBO 500S1 BBF-H-D-05 Z SP)
14	Number of Scans	99
15	Receiver Gain	188.8
16	Relaxation Delay	2.0000
17	Pulse Width	10.0000
18	Presaturation Frequency	
19	Acquisition Time	0.5505
20	Acquisition Date	2020-07-26T16:50:55
21	Modification Date	2020-07-26T16:50:58
22	Class	
23	Spectrometer Frequency	125.78
24	Spectral Width	29761.9
25	Lowest Frequency	-1627.5
26	Nucleus	13C
27	Acquired Size	16384
28	Spectral Size	65536

(5-(2-Fluorophenyl)-2-phenyl-1*H*-pyrrol-3-yl)(phenyl)methanone (5m)

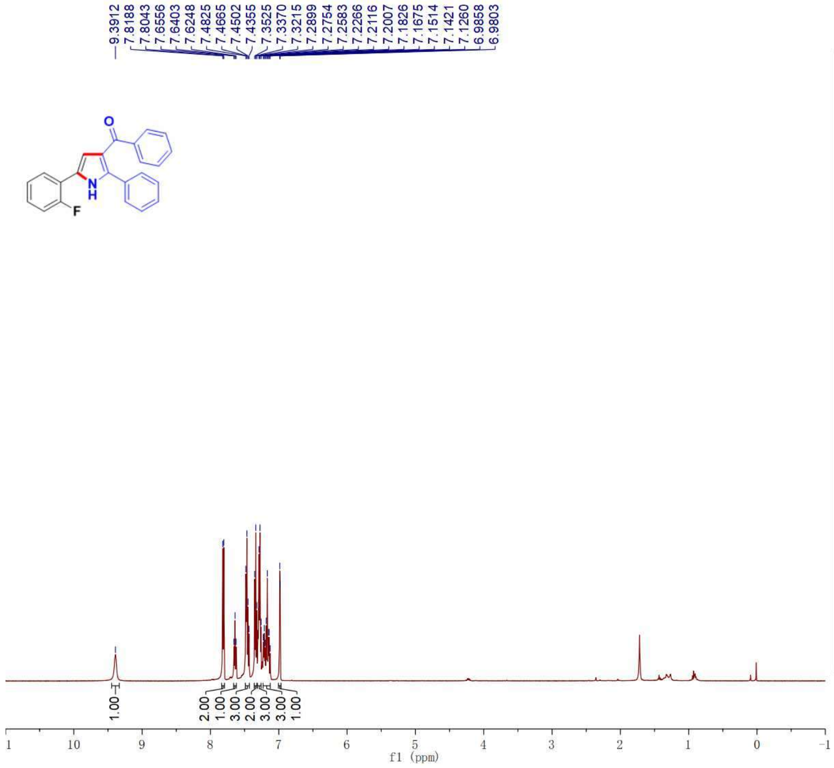

Parameters

	Parameter	Value
1	Data File Name	D:/ 核磁数据/ 20201011/ 2020-1-syj-H/ 292/ fid
2	标题	2020-1-syj-H.292.fid
3	Comment	1H ztt
4	Origin	Bruker BioSpin GmbH
5	Owner	nmrsu
6	Site	
7	Instrument	spect
8	Author	
9	Solvent	CDCl3
10	Temperature	292.8
11	Pulse Sequence	zg30
12	Experiment	1D
13	Probe	Z119470_0117 (PA BBO 500S1 BBF-H-D-05 Z SP)
14	Number of Scans	4
15	Receiver Gain	59.2
16	Relaxation Delay	2.0000
17	Pulse Width	12.0000
18	Presaturation Frequency	
19	Acquisition Time	1.8175
20	Acquisition Date	2020-08-10T11:02:25
21	Modification Date	2020-08-10T11:02:26
22	Class	
23	Spectrometer Frequency	500.16
24	Spectral Width	9014.4
25	Lowest Frequency	-1519.4
26	Nucleus	1H
27	Acquired Size	16384
28	Spectral Size	65536

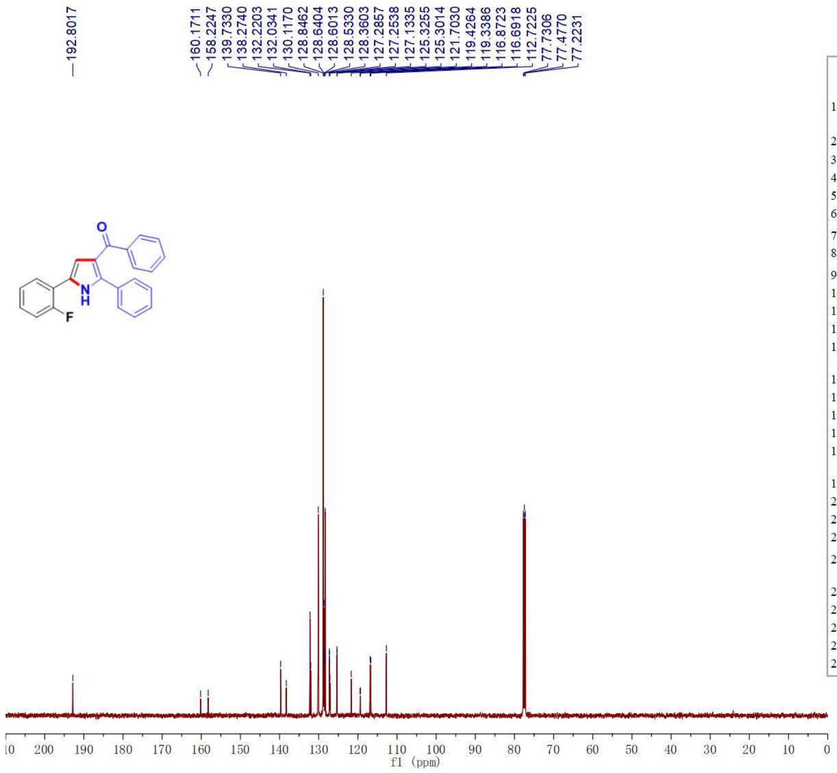

Parameters

	Parameter	Value
1	Data File Name	D:/ 核磁数据/ 20201011/ 2020-1-syj-C/ 130/ fid
2	标题	2020-1-syj-C.130.fid
3	Comment	13C ztt
4	Origin	Bruker BioSpin GmbH
5	Owner	nmrsu
6	Site	
7	Instrument	spect
8	Author	
9	Solvent	CDCl3
10	Temperature	293.6
11	Pulse Sequence	zgpg30
12	Experiment	1D
13	Probe	Z119470_0117 (PA BBO 500S1 BBF-H-D-05 Z SP)
14	Number of Scans	100
15	Receiver Gain	188.8
16	Relaxation Delay	2.0000
17	Pulse Width	10.0000
18	Presaturation Frequency	
19	Acquisition Time	0.5505
20	Acquisition Date	2020-08-10T12:41:04
21	Modification Date	2020-08-10T12:41:06
22	Class	
23	Spectrometer Frequency	125.78
24	Spectral Width	29761.9
25	Lowest Frequency	-1627.5
26	Nucleus	13C
27	Acquired Size	16384
28	Spectral Size	65536

(4-Chlorophenyl)(2-(4-chlorophenyl)-5-phenyl-1*H*-pyrrol-3-yl) methanone (5n)

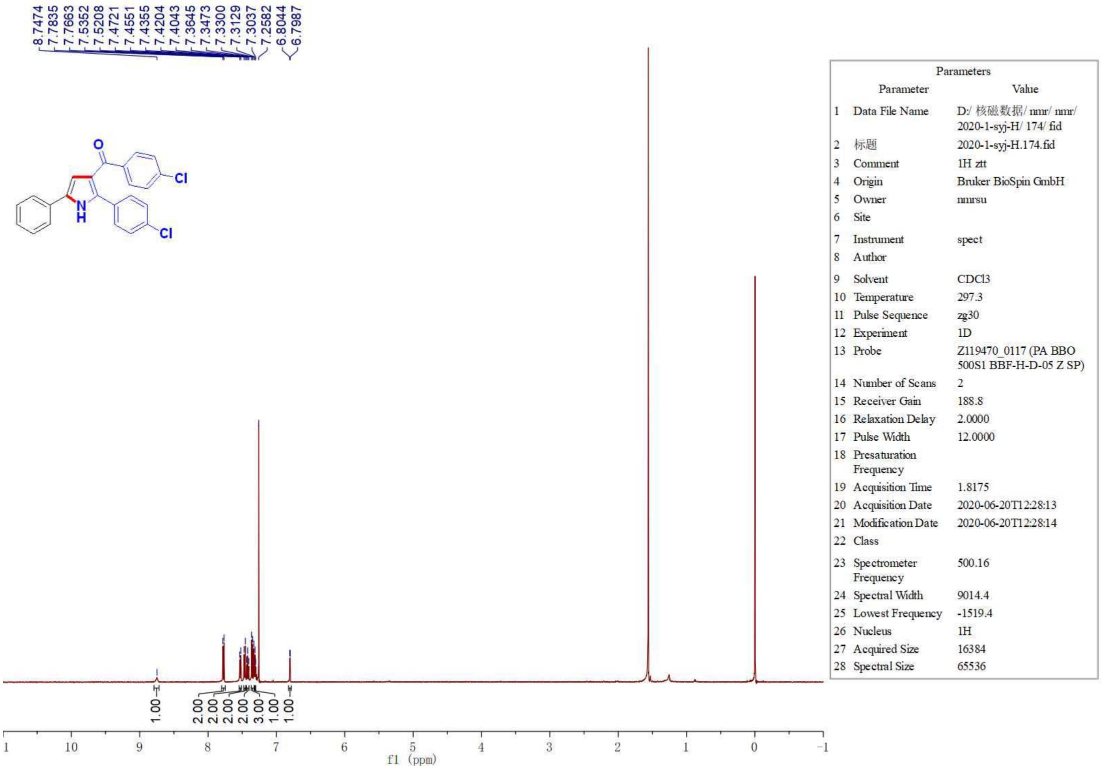

	Parameter	Value
1	Data File Name	D:/ 核磁数据/ nmr/ nmr/ 2020-1-syj-H/ 174/ fid
2	标题	2020-1-syj-H.174.fid
3	Comment	1H ztt
4	Origin	Bruker BioSpin GmbH
5	Owner	nmrsu
6	Site	
7	Instrument	spect
8	Author	
9	Solvent	CDCl3
10	Temperature	297.3
11	Pulse Sequence	zg30
12	Experiment	1D
13	Probe	Z119470_0117 (PA BBO 500S1 BBF-H-D-05 Z SP)
14	Number of Scans	2
15	Receiver Gain	188.8
16	Relaxation Delay	2.0000
17	Pulse Width	12.0000
18	Presaturation Frequency	
19	Acquisition Time	1.8175
20	Acquisition Date	2020-06-20T12:28:13
21	Modification Date	2020-06-20T12:28:14
22	Class	
23	Spectrometer Frequency	500.16
24	Spectral Width	9014.4
25	Lowest Frequency	-1519.4
26	Nucleus	1H
27	Acquired Size	16384
28	Spectral Size	65536

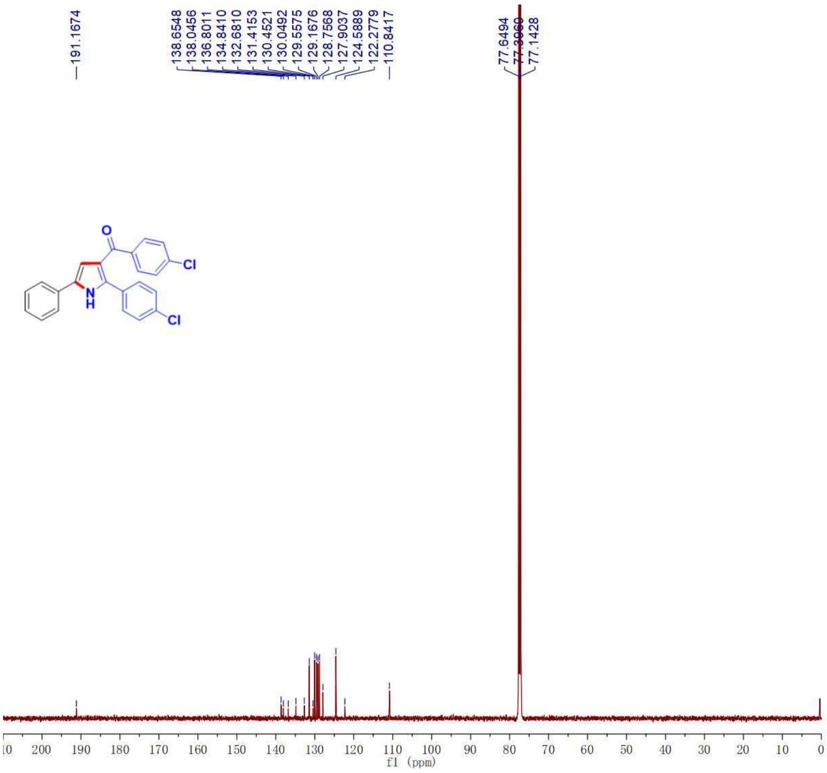

	Parameter	Value
1	Data File Name	D:/ 核磁数据/ nmr/ nmr/ 2020-1-syj-C/ 70/ fid
2	标题	2020-1-syj-C.70.fid
3	Comment	13C ztt
4	Origin	Bruker BioSpin GmbH
5	Owner	nmrsu
6	Site	
7	Instrument	spect
8	Author	
9	Solvent	CDCl3
10	Temperature	298.5
11	Pulse Sequence	zgpg30
12	Experiment	1D
13	Probe	Z119470_0117 (PA BBO 500S1 BBF-H-D-05 Z SP)
14	Number of Scans	4100
15	Receiver Gain	188.8
16	Relaxation Delay	2.0000
17	Pulse Width	10.0000
18	Presaturation Frequency	
19	Acquisition Time	0.5505
20	Acquisition Date	2020-06-20T19:10:40
21	Modification Date	2020-06-20T19:10:42
22	Class	
23	Spectrometer Frequency	125.78
24	Spectral Width	29761.9
25	Lowest Frequency	-1627.5
26	Nucleus	13C
27	Acquired Size	16384
28	Spectral Size	65536

(2,5-Diphenyl-1*H*-pyrrol-3-yl) (naphthalen-2-yl) methanone (5o)

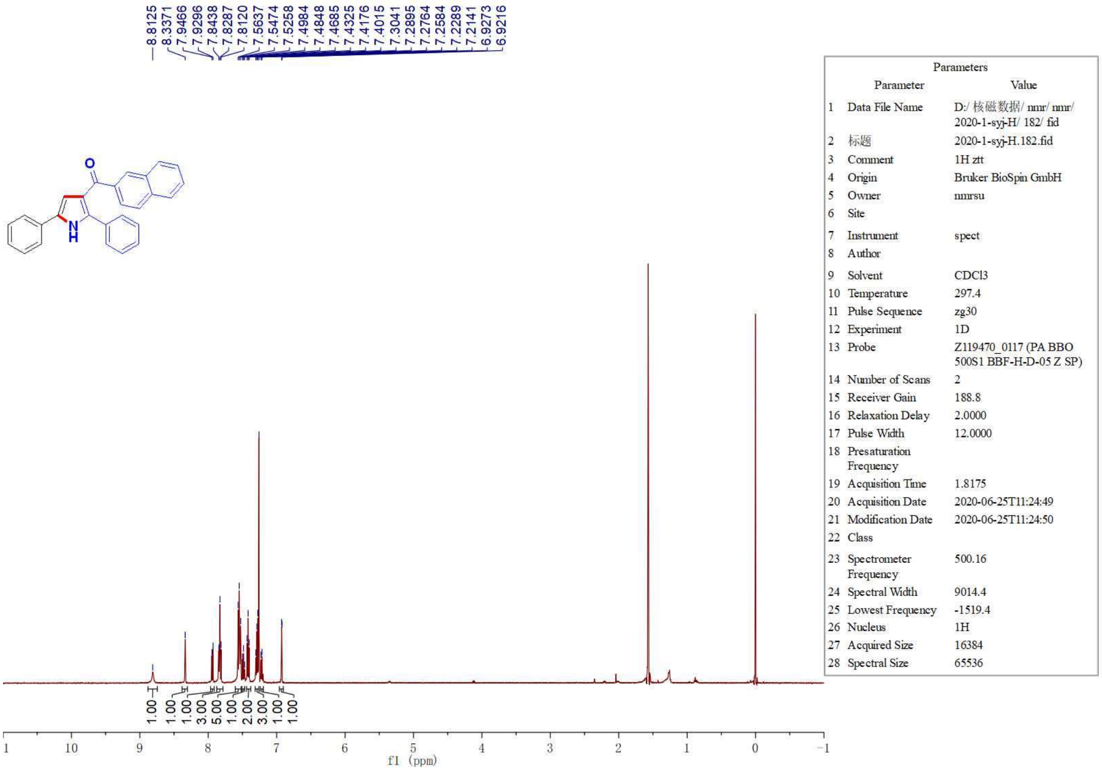

Parameters		
	Parameter	Value
1	Data File Name	D:/ 核磁数据/ nmr/ nmr/ 2020-1-syj-H/ 182/ fid
2	标题	2020-1-syj-H.182.fid
3	Comment	1H ztt
4	Origin	Bruker BioSpin GmbH
5	Owner	nmrsu
6	Site	
7	Instrument	spect
8	Author	
9	Solvent	CDCl3
10	Temperature	297.4
11	Pulse Sequence	zg30
12	Experiment	1D
13	Probe	Z119470_0117 (PA BBO 500S1 BBF-H-D-05 Z SP)
14	Number of Scans	2
15	Receiver Gain	188.8
16	Relaxation Delay	2.0000
17	Pulse Width	12.0000
18	Presaturation Frequency	
19	Acquisition Time	1.8175
20	Acquisition Date	2020-06-25T11:24:49
21	Modification Date	2020-06-25T11:24:50
22	Class	
23	Spectrometer Frequency	500.16
24	Spectral Width	9014.4
25	Lowest Frequency	-1519.4
26	Nucleus	1H
27	Acquired Size	16384
28	Spectral Size	65536

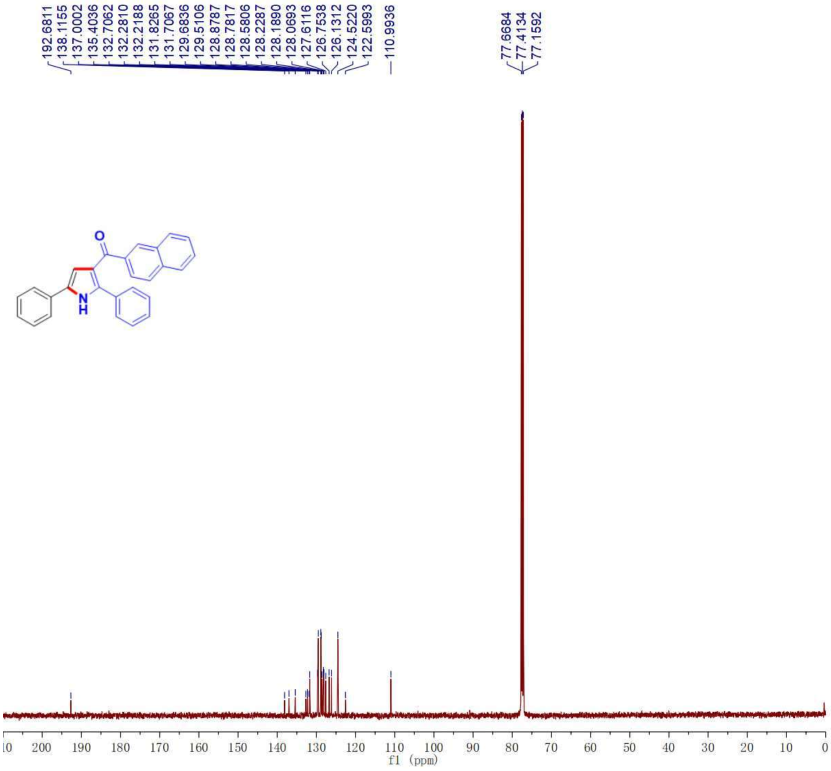

Parameters		
	Parameter	Value
1	Data File Name	D:/ 核磁数据/ nmr/ nmr/ 2020-1-syj-C/ 77/ fid
2	标题	2020-1-syj-C.77.fid
3	Comment	13C ztt
4	Origin	Bruker BioSpin GmbH
5	Owner	nmrsu
6	Site	
7	Instrument	spect
8	Author	
9	Solvent	CDCl3
10	Temperature	298.2
11	Pulse Sequence	zgpg30
12	Experiment	1D
13	Probe	Z119470_0117 (PA BBO 500S1 BBF-H-D-05 Z SP)
14	Number of Scans	600
15	Receiver Gain	188.8
16	Relaxation Delay	2.0000
17	Pulse Width	10.0000
18	Presaturation Frequency	
19	Acquisition Time	0.5505
20	Acquisition Date	2020-06-26T07:52:58
21	Modification Date	2020-06-26T07:53:00
22	Class	
23	Spectrometer Frequency	125.78
24	Spectral Width	29761.9
25	Lowest Frequency	-1627.5
26	Nucleus	13C
27	Acquired Size	16384
28	Spectral Size	65536

(2,5-Diphenyl-1*H*-pyrrol-3-yl) (thiophen-2-yl) methanone (5p)

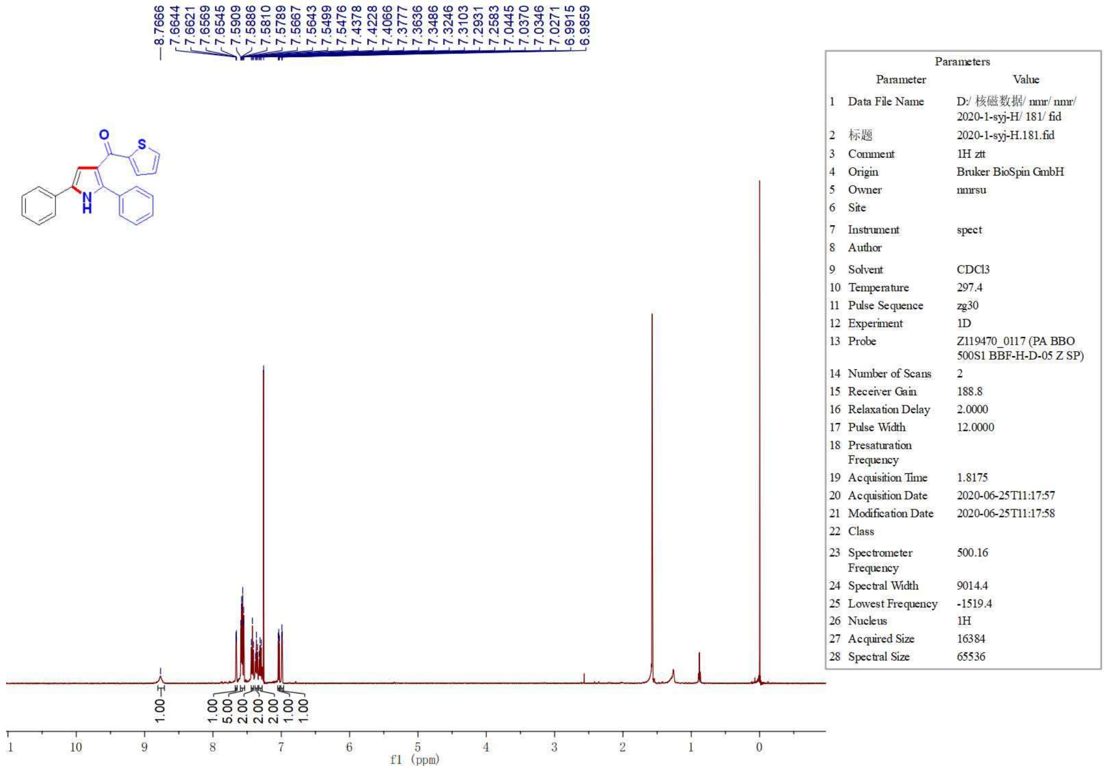

Parameter		Value
1	Data File Name	D:/ 核磁数据/ nmr/ nmr/ 2020-1-syj-H/ 181/ fid
2	标题	2020-1-syj-H.181.fid
3	Comment	1H ztt
4	Origin	Bruker BioSpin GmbH
5	Owner	nmrsu
6	Site	
7	Instrument	spect
8	Author	
9	Solvent	CDCl3
10	Temperature	297.4
11	Pulse Sequence	zg30
12	Experiment	1D
13	Probe	Z119470_0117 (PA BBO 500S1 BBF-H-D-05 Z SP)
14	Number of Scans	2
15	Receiver Gain	188.8
16	Relaxation Delay	2.0000
17	Pulse Width	12.0000
18	Presaturation Frequency	
19	Acquisition Time	1.8175
20	Acquisition Date	2020-06-25T11:17:57
21	Modification Date	2020-06-25T11:17:58
22	Class	
23	Spectrometer Frequency	500.16
24	Spectral Width	9014.4
25	Lowest Frequency	-1519.4
26	Nucleus	1H
27	Acquired Size	16384
28	Spectral Size	65536

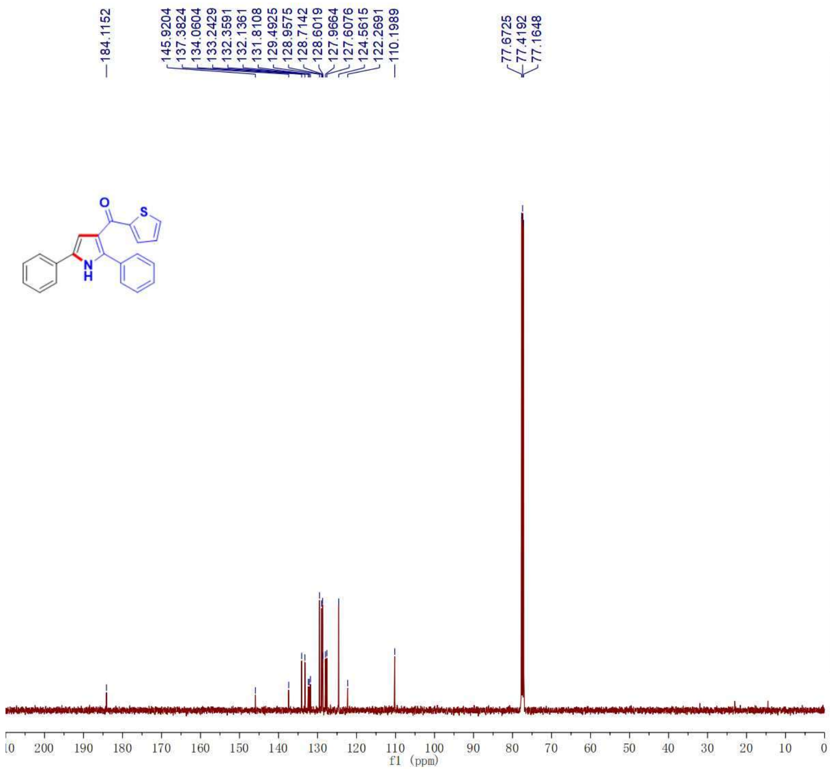

Parameter		Value
1	Data File Name	D:/ 核磁数据/ nmr/ nmr/ 2020-1-syj-C/ 76/ fid
2	标题	2020-1-syj-C.76.fid
3	Comment	13C ztt
4	Origin	Bruker BioSpin GmbH
5	Owner	nmrsu
6	Site	
7	Instrument	spect
8	Author	
9	Solvent	CDCl3
10	Temperature	298.5
11	Pulse Sequence	zgpg30
12	Experiment	1D
13	Probe	Z119470_0117 (PA BBO 500S1 BBF-H-D-05 Z SP)
14	Number of Scans	254
15	Receiver Gain	188.8
16	Relaxation Delay	2.0000
17	Pulse Width	10.0000
18	Presaturation Frequency	
19	Acquisition Time	0.5505
20	Acquisition Date	2020-06-26T07:24:08
21	Modification Date	2020-06-26T07:24:10
22	Class	
23	Spectrometer Frequency	125.78
24	Spectral Width	29761.9
25	Lowest Frequency	-1627.5
26	Nucleus	13C
27	Acquired Size	16384
28	Spectral Size	65536

(2-Isopropyl-5-phenyl-1*H*-pyrrol-3-yl) (phenyl)methanone (5q)

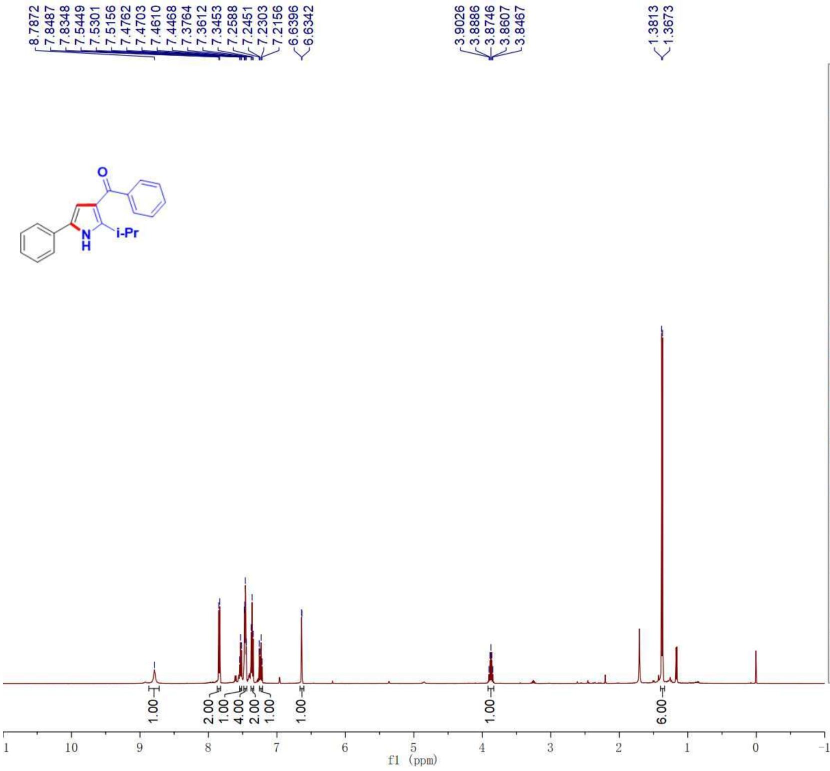

	Parameter	Value
	**Parameters**	
1	Data File Name	D:/ 核磁数据/ 20201011/ 2020-1-syj-H/ 295/ fid
2	标题	2020-1-syj-H.295.fid
3	Comment	1H ztt
4	Origin	Bruker BioSpin GmbH
5	Owner	nmrsu
6	Site	
7	Instrument	spect
8	Author	
9	Solvent	CDCl3
10	Temperature	292.9
11	Pulse Sequence	zg30
12	Experiment	1D
13	Probe	Z119470_0117 (PA BBO 500S1 BBF-H-D-05 Z SP)
14	Number of Scans	4
15	Receiver Gain	66.5
16	Relaxation Delay	2.0000
17	Pulse Width	12.0000
18	Presaturation Frequency	
19	Acquisition Time	1.8175
20	Acquisition Date	2020-08-10T11:24:54
21	Modification Date	2020-08-10T11:24:56
22	Class	
23	Spectrometer Frequency	500.16
24	Spectral Width	9014.4
25	Lowest Frequency	-1519.4
26	Nucleus	1H
27	Acquired Size	16384
28	Spectral Size	65536

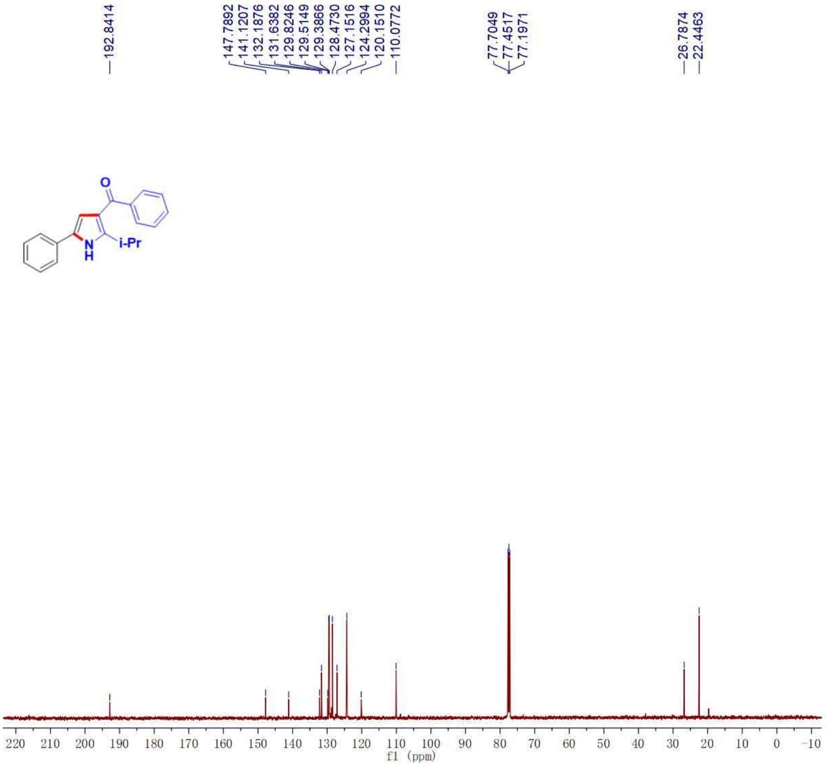

	Parameter	Value
	**Parameters**	
1	Data File Name	D:/ 核磁数据/ 20201011/ 2020-1-syj-C/ 133/ fid
2	标题	2020-1-syj-C.133.fid
3	Comment	13C ztt
4	Origin	Bruker BioSpin GmbH
5	Owner	nmrsu
6	Site	
7	Instrument	spect
8	Author	
9	Solvent	CDCl3
10	Temperature	294.0
11	Pulse Sequence	zgpg30
12	Experiment	1D
13	Probe	Z119470_0117 (PA BBO 500S1 BBF-H-D-05 Z SP)
14	Number of Scans	120
15	Receiver Gain	188.8
16	Relaxation Delay	2.0000
17	Pulse Width	10.0000
18	Presaturation Frequency	
19	Acquisition Time	0.5505
20	Acquisition Date	2020-08-10T13:09:58
21	Modification Date	2020-08-10T13:10:00
22	Class	
23	Spectrometer Frequency	125.78
24	Spectral Width	29761.9
25	Lowest Frequency	-1627.5
26	Nucleus	13C
27	Acquired Size	16384
28	Spectral Size	65536

Phenyl(5-phenyl-2-propyl-1*H*-pyrrol-3-yl) methanone (5r)

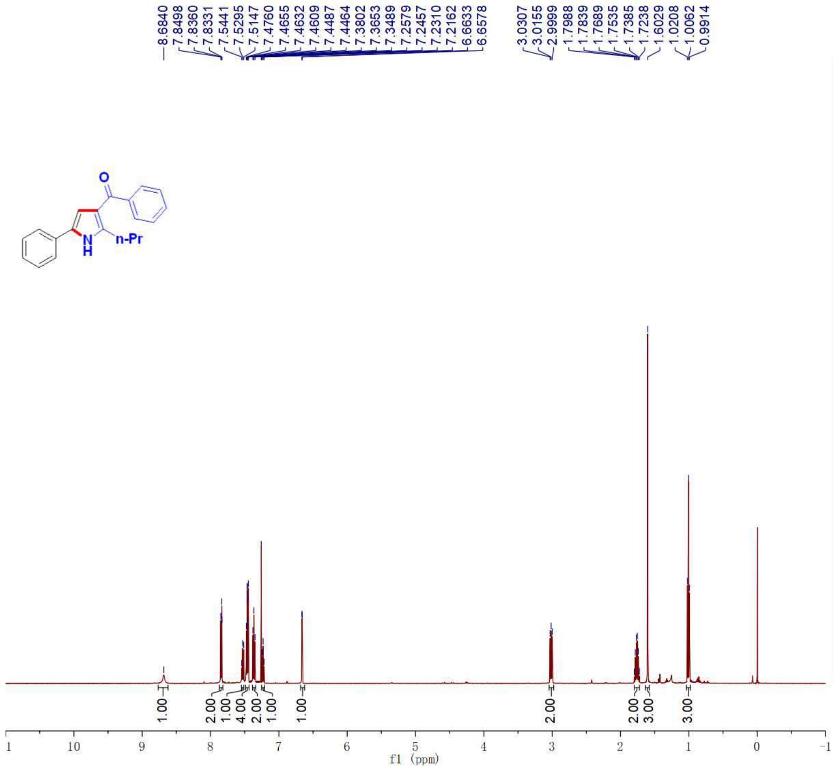

Parameters		
	Parameter	Value
1	Data File Name	D:/ 核磁数据/ nmr/ nmr/ 2020-1-syj-H/ 225/ fid
2	标题	2020-1-syj-H.225.fid
3	Comment	1H ztt
4	Origin	Bruker BioSpin GmbH
5	Owner	nmrsu
6	Site	
7	Instrument	spect
8	Author	
9	Solvent	CDCl3
10	Temperature	297.4
11	Pulse Sequence	zg30
12	Experiment	1D
13	Probe	Z119470_0117 (PA BBO 500S1 BBF-H-D-05 Z SP)
14	Number of Scans	2
15	Receiver Gain	128.6
16	Relaxation Delay	2.0000
17	Pulse Width	12.0000
18	Presaturation Frequency	
19	Acquisition Time	1.8175
20	Acquisition Date	2020-07-13T17:00:08
21	Modification Date	2020-07-13T17:00:10
22	Class	
23	Spectrometer Frequency	500.16
24	Spectral Width	9014.4
25	Lowest Frequency	-1519.4
26	Nucleus	1H
27	Acquired Size	16384
28	Spectral Size	65536

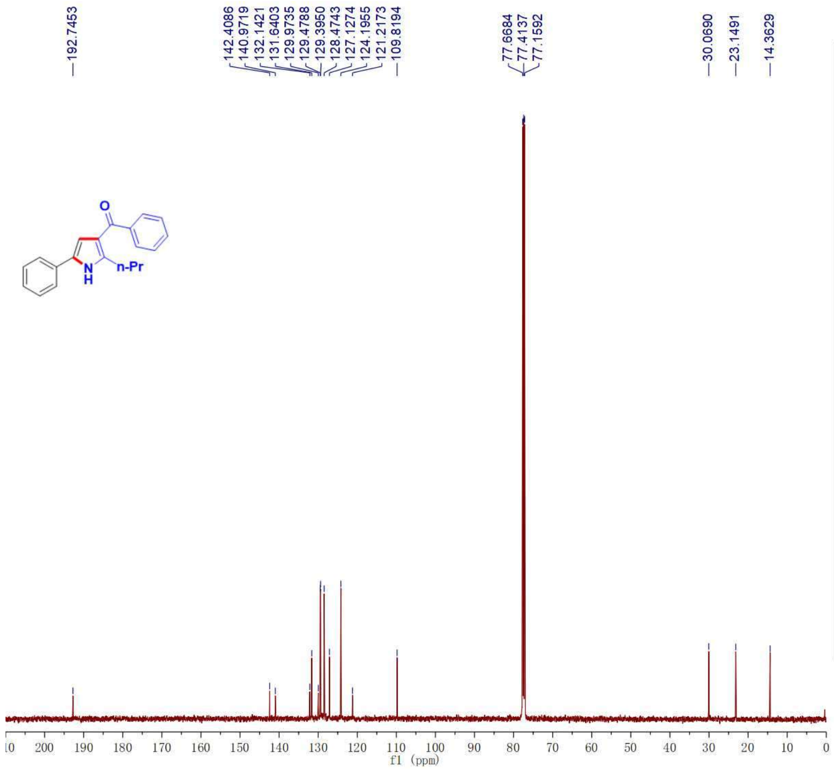

Parameters		
	Parameter	Value
1	Data File Name	D:/ 核磁数据/ nmr/ nmr/ 2020-1-syj-C/ 88/ fid
2	标题	2020-1-syj-C.88.fid
3	Comment	13C ztt
4	Origin	Bruker BioSpin GmbH
5	Owner	nmrsu
6	Site	
7	Instrument	spect
8	Author	
9	Solvent	CDCl3
10	Temperature	298.3
11	Pulse Sequence	zgpg30
12	Experiment	1D
13	Probe	Z119470_0117 (PA BBO 500S1 BBF-H-D-05 Z SP)
14	Number of Scans	660
15	Receiver Gain	188.8
16	Relaxation Delay	2.0000
17	Pulse Width	10.0000
18	Presaturation Frequency	
19	Acquisition Time	0.5505
20	Acquisition Date	2020-07-14T09:50:55
21	Modification Date	2020-07-14T09:50:58
22	Class	
23	Spectrometer Frequency	125.78
24	Spectral Width	29761.9
25	Lowest Frequency	-1627.5
26	Nucleus	13C
27	Acquired Size	16384
28	Spectral Size	65536

(2-Butyl-5-phenyl-1*H*-pyrrol-3-yl) (phenyl)methanone (5s)

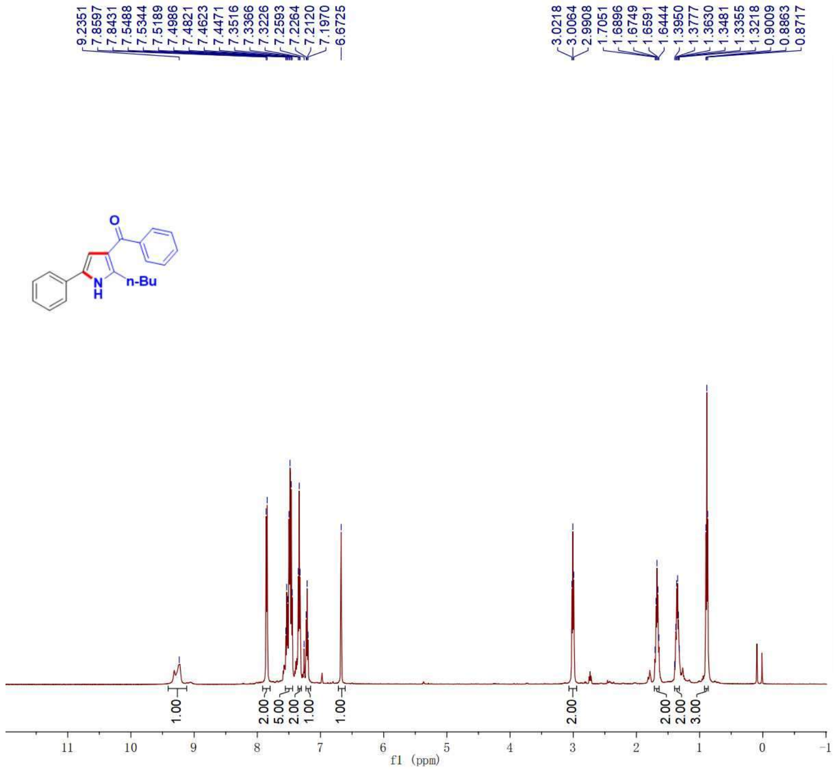

	Parameters	
	Parameter	Value
1	Data File Name	D:/ 核磁数据/ 2021-syj-500/ 2021-1-syj-H/ 6/ fid
2	标题	2021-1-syj-H.6.fid
3	Comment	1H ztt
4	Origin	Bruker BioSpin GmbH
5	Owner	nmrsu
6	Site	
7	Instrument	spect
8	Author	
9	Solvent	CDCl3
10	Temperature	291.5
11	Pulse Sequence	zg30
12	Experiment	1D
13	Probe	Z119470_0117 (PA BBO 500S1 BBF-H-D-05 Z SP)
14	Number of Scans	2
15	Receiver Gain	53.4
16	Relaxation Delay	2.0000
17	Pulse Width	12.0000
18	Presaturation Frequency	
19	Acquisition Time	1.8175
20	Acquisition Date	2021-01-10T16:00:03
21	Modification Date	2021-01-10T16:00:04
22	Class	
23	Spectrometer Frequency	500.16
24	Spectral Width	9014.4
25	Lowest Frequency	-1519.4
26	Nucleus	1H
27	Acquired Size	16384
28	Spectral Size	65536

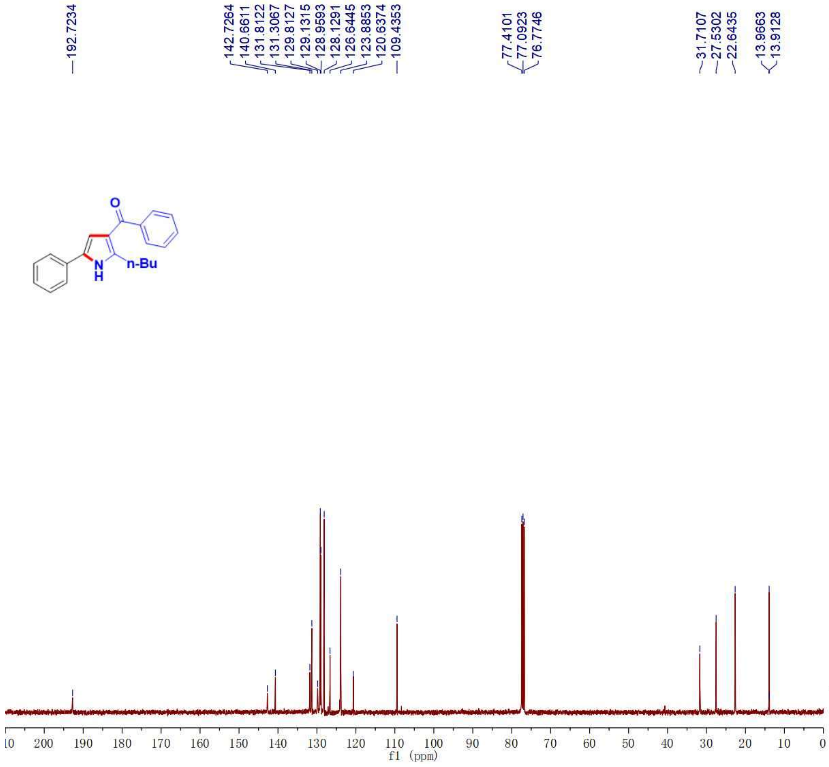

	Parameters	
	Parameter	Value
1	Data File Name	D:/ 核磁数据/ 400/ 2021-1-syj-C/ 13/ fid
2	标题	2021-1-syj-C.13.fid
3	Comment	
4	Origin	Bruker BioSpin GmbH
5	Owner	nmrsu
6	Site	
7	Instrument	Avance
8	Author	
9	Solvent	CDCl3
10	Temperature	294.7
11	Pulse Sequence	zgpg30
12	Experiment	1D
13	Probe	Z163739_0032 (PI HR-400-S1-BBF/ H/ D-5.0-Z SP)
14	Number of Scans	191
15	Receiver Gain	48.1
16	Relaxation Delay	2.0000
17	Pulse Width	10.0000
18	Presaturation Frequency	
19	Acquisition Time	1.3763
20	Acquisition Date	2021-01-17T14:08:08
21	Modification Date	2021-01-17T14:07:32
22	Class	
23	Spectrometer Frequency	100.62
24	Spectral Width	23809.5
25	Lowest Frequency	-1843.5
26	Nucleus	13C
27	Acquired Size	32768
28	Spectral Size	65536

(1,2-Dimethyl-5-phenyl-1*H*-pyrrol-3-yl) (phenyl)methanone (7a)

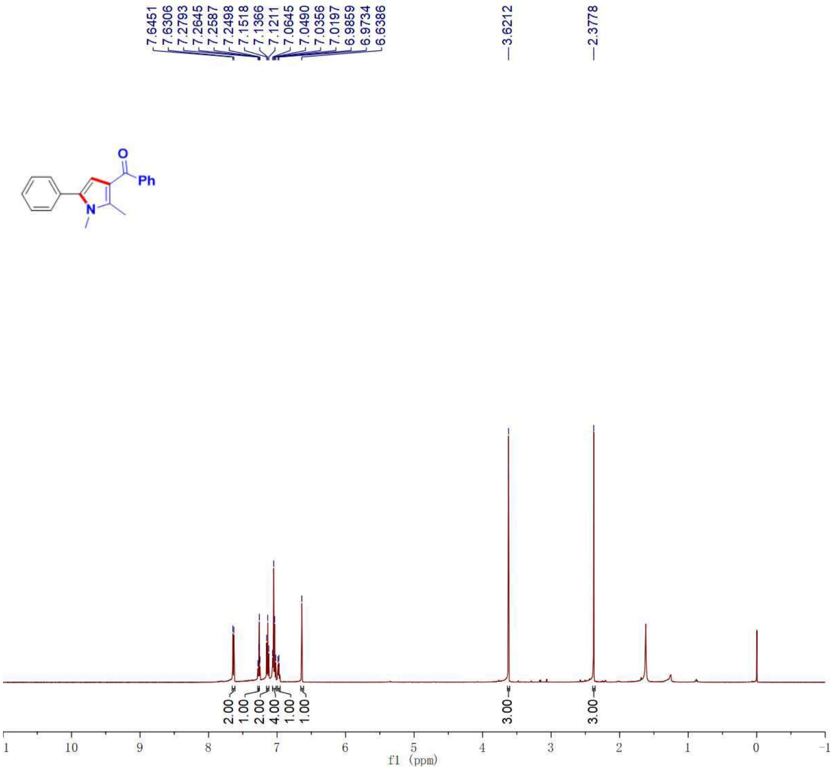

Parameters		
	Parameter	Value
1	Data File Name	D:/ 核磁数据/ 20201011/ 2020-1-syj-H/ 305/ fid
2	标题	2020-1-syj-H.305.fid
3	Comment	1H ztt
4	Origin	Bruker BioSpin GmbH
5	Owner	nmrsu
6	Site	
7	Instrument	spect
8	Author	
9	Solvent	CDCl3
10	Temperature	293.7
11	Pulse Sequence	zg30
12	Experiment	1D
13	Probe	Z119470_0117 (PA BBO 500S1 BBF-H-D-05 Z SP)
14	Number of Scans	4
15	Receiver Gain	114.1
16	Relaxation Delay	2.0000
17	Pulse Width	12.0000
18	Presaturation Frequency	
19	Acquisition Time	1.8175
20	Acquisition Date	2020-08-14T11:19:29
21	Modification Date	2020-08-14T11:19:30
22	Class	
23	Spectrometer Frequency	500.16
24	Spectral Width	9014.4
25	Lowest Frequency	-1519.4
26	Nucleus	1H
27	Acquired Size	16384
28	Spectral Size	65536

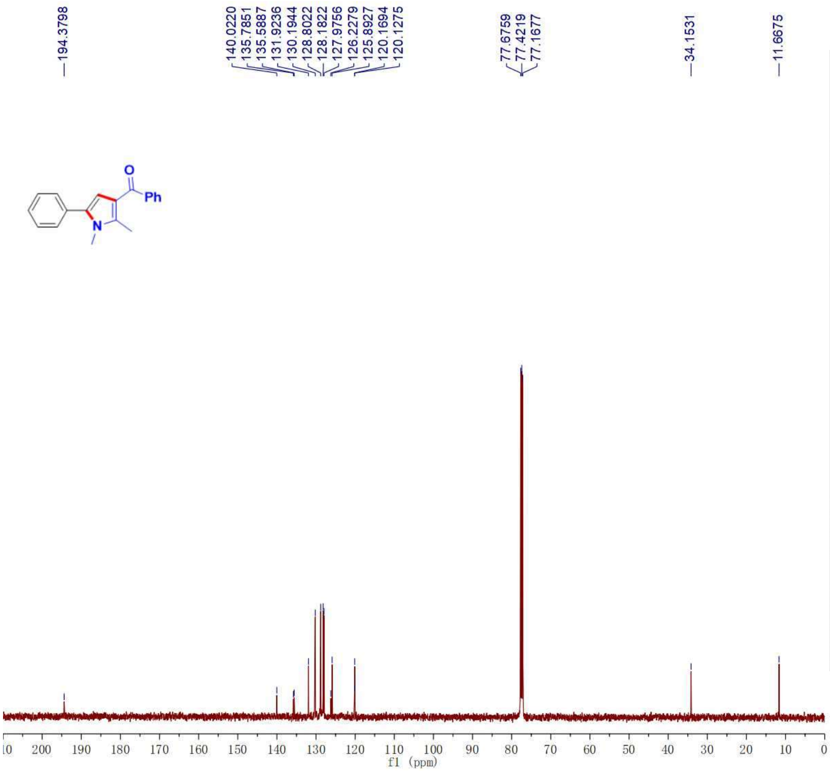

Parameters		
	Parameter	Value
1	Data File Name	D:/ 核磁数据/ 20201011/ 2020-1-syj-C/ 138/ fid
2	标题	2020-1-syj-C.138.fid
3	Comment	13C ztt
4	Origin	Bruker BioSpin GmbH
5	Owner	nmrsu
6	Site	
7	Instrument	spect
8	Author	
9	Solvent	CDCl3
10	Temperature	297.2
11	Pulse Sequence	zgpg30
12	Experiment	1D
13	Probe	Z119470_0117 (PA BBO 500S1 BBF-H-D-05 Z SP)
14	Number of Scans	208
15	Receiver Gain	188.8
16	Relaxation Delay	2.0000
17	Pulse Width	10.0000
18	Presaturation Frequency	
19	Acquisition Time	0.5505
20	Acquisition Date	2020-08-16T17:48:08
21	Modification Date	2020-08-16T17:48:10
22	Class	
23	Spectrometer Frequency	125.78
24	Spectral Width	29761.9
25	Lowest Frequency	-1627.5
26	Nucleus	13C
27	Acquired Size	16384
28	Spectral Size	65536

1-(2-Methyl-5-phenyl-1-(*p*-tolyl)-1*H*-pyrrol-3-yl) ethan-1-one (7b)

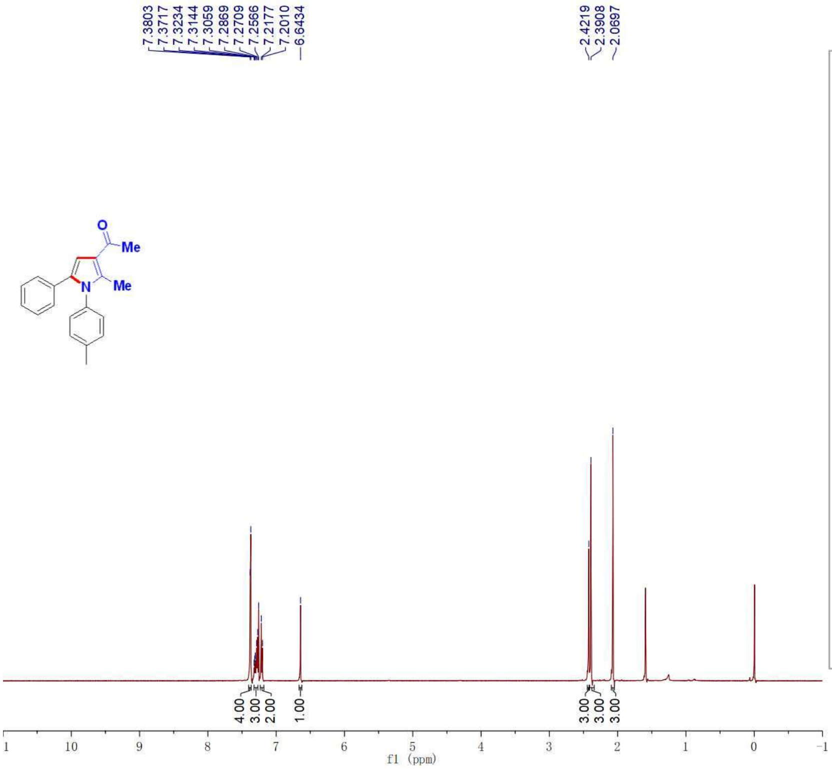

Parameter		Value
1	Data File Name	D:/ 核磁数据/ nmr/ nmr/ 2020-1-syj-H/ 23/ fid
2	标题	2020-1-syj-H.23.fid
3	Comment	1H ztt
4	Origin	Bruker BioSpin GmbH
5	Owner	root
6	Site	
7	Instrument	spect
8	Author	
9	Solvent	CDCl3
10	Temperature	293.8
11	Pulse Sequence	zg30
12	Experiment	1D
13	Probe	Z119470_0117 (PA BBO 500S1 BBF-H-D-05 Z SP)
14	Number of Scans	2
15	Receiver Gain	114.1
16	Relaxation Delay	2.0000
17	Pulse Width	12.0000
18	Presaturation Frequency	
19	Acquisition Time	1.8175
20	Acquisition Date	2020-01-02T11:31:26
21	Modification Date	2020-01-02T11:31:28
22	Class	
23	Spectrometer Frequency	500.16
24	Spectral Width	9014.4
25	Lowest Frequency	-1519.4
26	Nucleus	1H
27	Acquired Size	16384
28	Spectral Size	65536

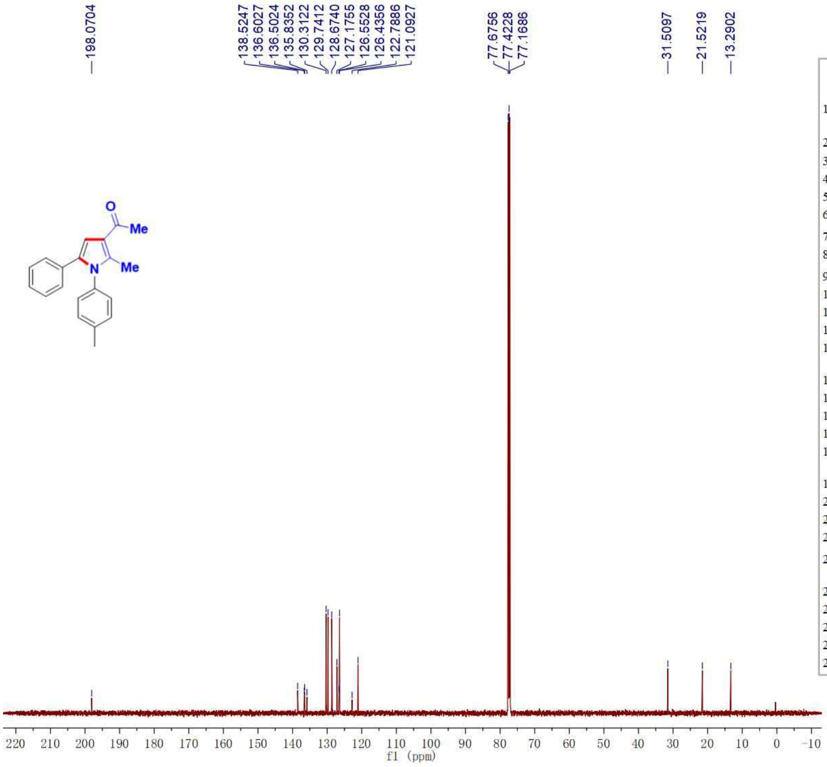

Parameter		Value
1	Data File Name	D:/ 核磁数据/ nmr/ nmr/ 2020-1-syj-C/ 7/ fid
2	标题	2020-1-syj-C.7.fid
3	Comment	13C ztt
4	Origin	Bruker BioSpin GmbH
5	Owner	root
6	Site	
7	Instrument	spect
8	Author	
9	Solvent	CDCl3
10	Temperature	294.0
11	Pulse Sequence	zgpg30
12	Experiment	1D
13	Probe	Z119470_0117 (PA BBO 500S1 BBF-H-D-05 Z SP)
14	Number of Scans	500
15	Receiver Gain	188.8
16	Relaxation Delay	2.0000
17	Pulse Width	10.0000
18	Presaturation Frequency	
19	Acquisition Time	0.5505
20	Acquisition Date	2020-01-02T12:14:21
21	Modification Date	2020-01-02T12:14:24
22	Class	
23	Spectrometer Frequency	125.78
24	Spectral Width	29761.9
25	Lowest Frequency	-1627.5
26	Nucleus	13C
27	Acquired Size	16384
28	Spectral Size	65536

2-Methyl-4-phenyl-1*H*-imidazole (8)

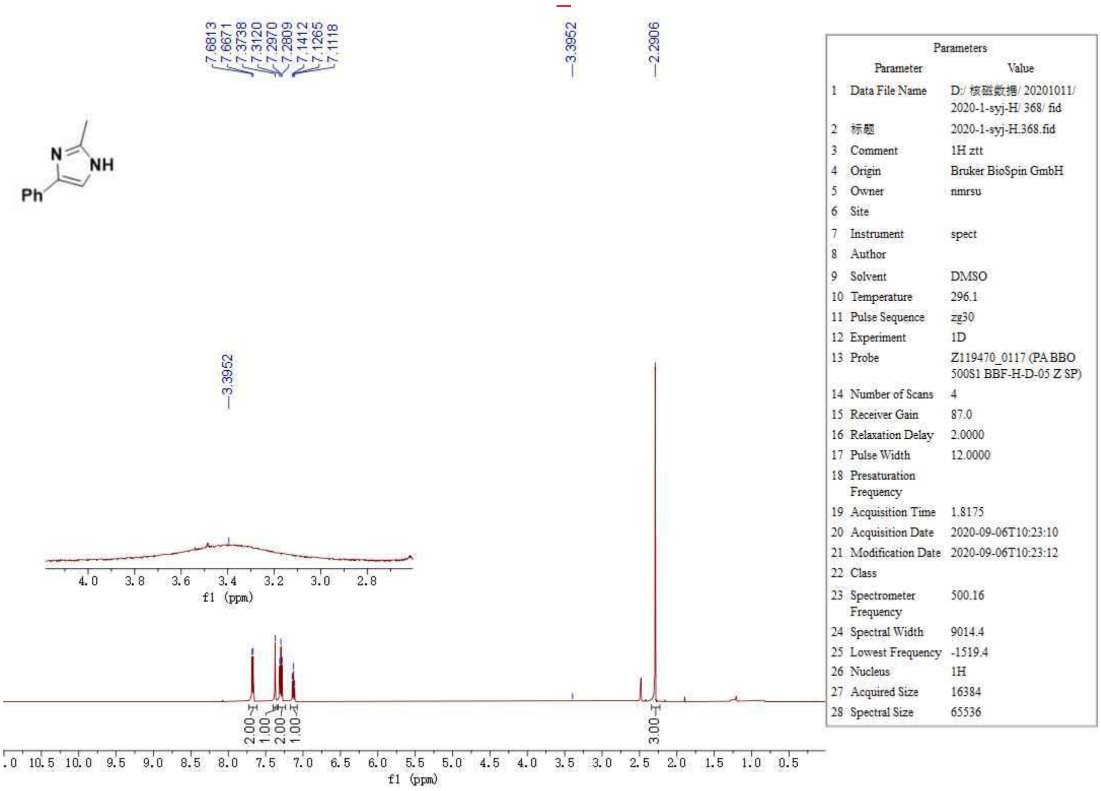

Parameter		Value
1	Data File Name	D:/ 核磁数据/ 20201011/ 2020-1-syj-H/ 368/ fid
2	标题	2020-1-syj-H.368.fid
3	Comment	1H ztt
4	Origin	Bruker BioSpin GmbH
5	Owner	nmrsu
6	Site	
7	Instrument	spect
8	Author	
9	Solvent	DMSO
10	Temperature	296.1
11	Pulse Sequence	zg30
12	Experiment	1D
13	Probe	Z119470_0117 (PA BBO 500S1 BBF-H-D-05 Z SP)
14	Number of Scans	4
15	Receiver Gain	87.0
16	Relaxation Delay	2.0000
17	Pulse Width	12.0000
18	Presaturation Frequency	
19	Acquisition Time	1.8175
20	Acquisition Date	2020-09-06T10:23:10
21	Modification Date	2020-09-06T10:23:12
22	Class	
23	Spectrometer Frequency	500.16
24	Spectral Width	9014.4
25	Lowest Frequency	-1519.4
26	Nucleus	1H
27	Acquired Size	16384
28	Spectral Size	65536

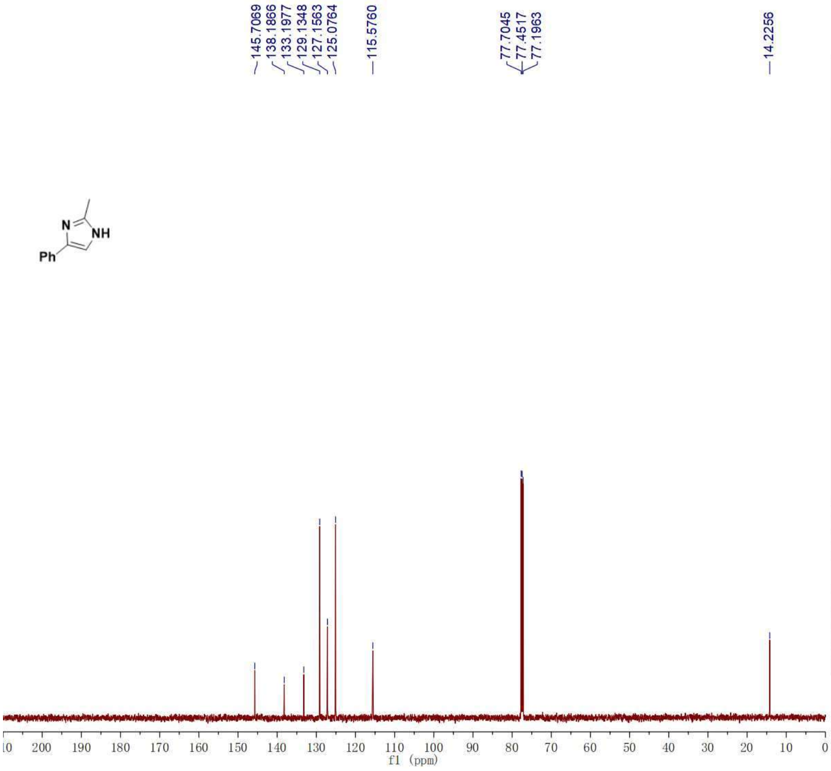

Parameter		Value
1	Data File Name	D:/ 核磁数据/ 20201011/ 2020-1-syj-C/ 160/ fid
2	标题	2020-1-syj-C.160.fid
3	Comment	13C ztt
4	Origin	Bruker BioSpin GmbH
5	Owner	nmrsu
6	Site	
7	Instrument	spect
8	Author	
9	Solvent	CDCl3
10	Temperature	296.7
11	Pulse Sequence	zgpg30
12	Experiment	1D
13	Probe	Z119470_0117 (PA BBO 500S1 BBF-H-D-05 Z SP)
14	Number of Scans	47
15	Receiver Gain	188.8
16	Relaxation Delay	2.0000
17	Pulse Width	10.0000
18	Presaturation Frequency	
19	Acquisition Time	0.5505
20	Acquisition Date	2020-09-03T10:59:11
21	Modification Date	2020-09-03T10:59:14
22	Class	
23	Spectrometer Frequency	125.78
24	Spectral Width	29761.9
25	Lowest Frequency	-1627.5
26	Nucleus	13C
27	Acquired Size	16384
28	Spectral Size	65536

(*E*)-(2,5-Diphenyl-1*H*-pyrrol-3-yl) (phenyl)methanone oxime (9)

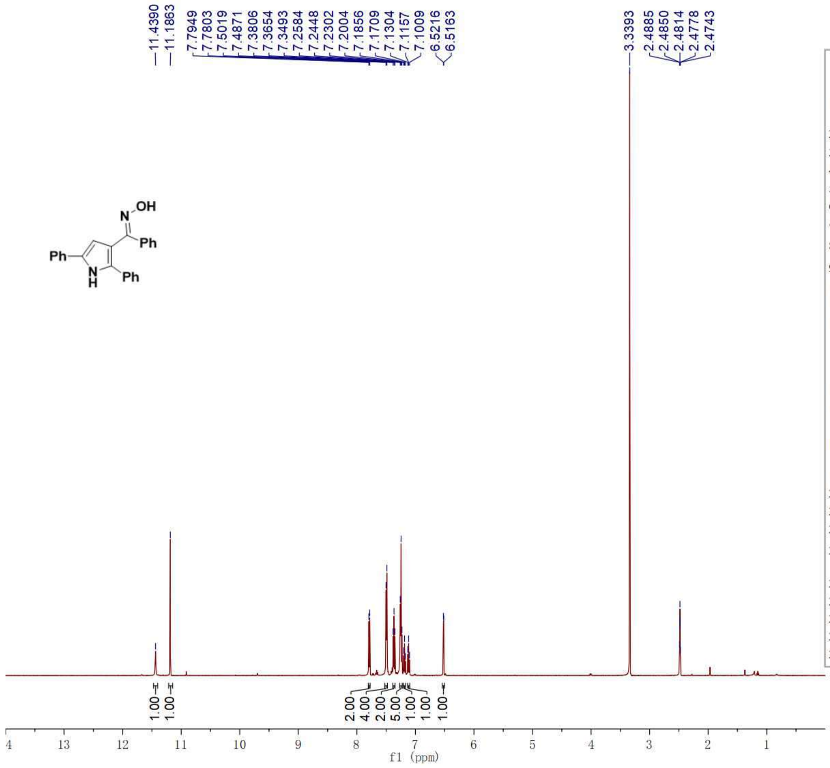

Parameters		
	Parameter	Value
1	Data File Name	D:/ 核磁数据/ 20201011/ 2020-1-syj-H/ 367/ fid
2	标题	2020-1-syj-H.367.fid
3	Comment	1H ztt
4	Origin	Bruker BioSpin GmbH
5	Owner	nmrsu
6	Site	
7	Instrument	spect
8	Author	
9	Solvent	DMSO
10	Temperature	296.1
11	Pulse Sequence	zg30
12	Experiment	1D
13	Probe	Z119470_0117 (PA BBO 500S1 BBF-H-D-05 Z SP)
14	Number of Scans	4
15	Receiver Gain	87.0
16	Relaxation Delay	2.0000
17	Pulse Width	12.0000
18	Presaturation Frequency	
19	Acquisition Time	1.8175
20	Acquisition Date	2020-09-06T10:18:44
21	Modification Date	2020-09-06T10:18:46
22	Class	
23	Spectrometer Frequency	500.16
24	Spectral Width	9014.4
25	Lowest Frequency	-1519.4
26	Nucleus	1H
27	Acquired Size	16384
28	Spectral Size	65536

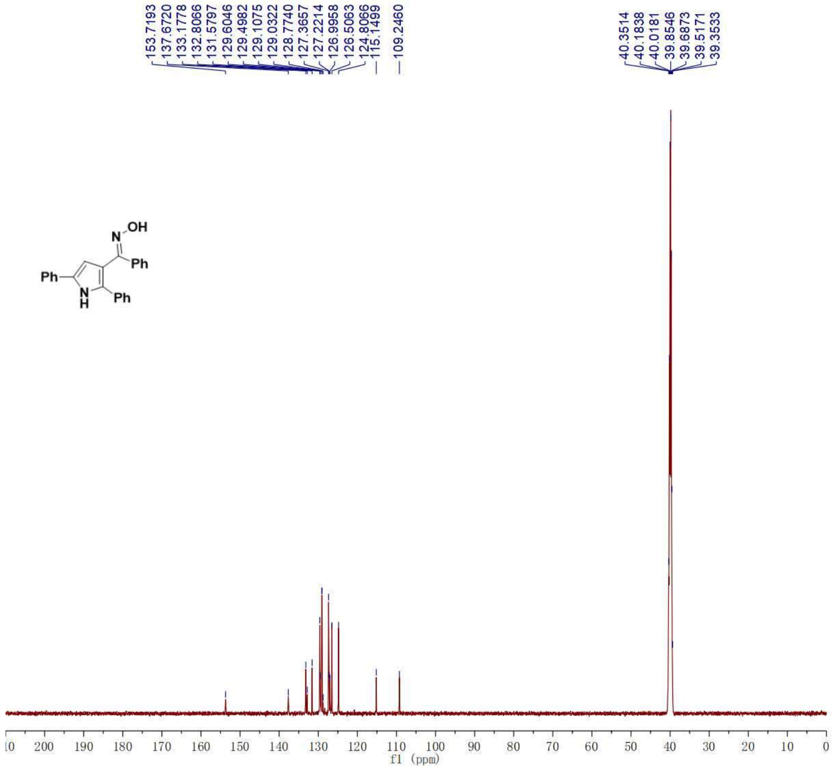

Parameters		
	Parameter	Value
1	Data File Name	D:/ 核磁数据/ 20201011/ 2020-1-syj-C/ 166/ fid
2	标题	2020-1-syj-C.166.fid
3	Comment	13C ztt
4	Origin	Bruker BioSpin GmbH
5	Owner	nmrsu
6	Site	
7	Instrument	spect
8	Author	
9	Solvent	DMSO
10	Temperature	296.7
11	Pulse Sequence	zgpg30
12	Experiment	1D
13	Probe	Z119470_0117 (PA BBO 500S1 BBF-H-D-05 Z SP)
14	Number of Scans	1105
15	Receiver Gain	188.8
16	Relaxation Delay	2.0000
17	Pulse Width	10.0000
18	Presaturation Frequency	
19	Acquisition Time	0.5505
20	Acquisition Date	2020-09-07T17:17:40
21	Modification Date	2020-09-07T17:17:42
22	Class	
23	Spectrometer Frequency	125.78
24	Spectral Width	29761.9
25	Lowest Frequency	-1627.5
26	Nucleus	13C
27	Acquired Size	16384
28	Spectral Size	65536

(1-Methyl-2,5-diphenyl-1*H*-pyrrol-3-yl) (phenyl)methanone (10)

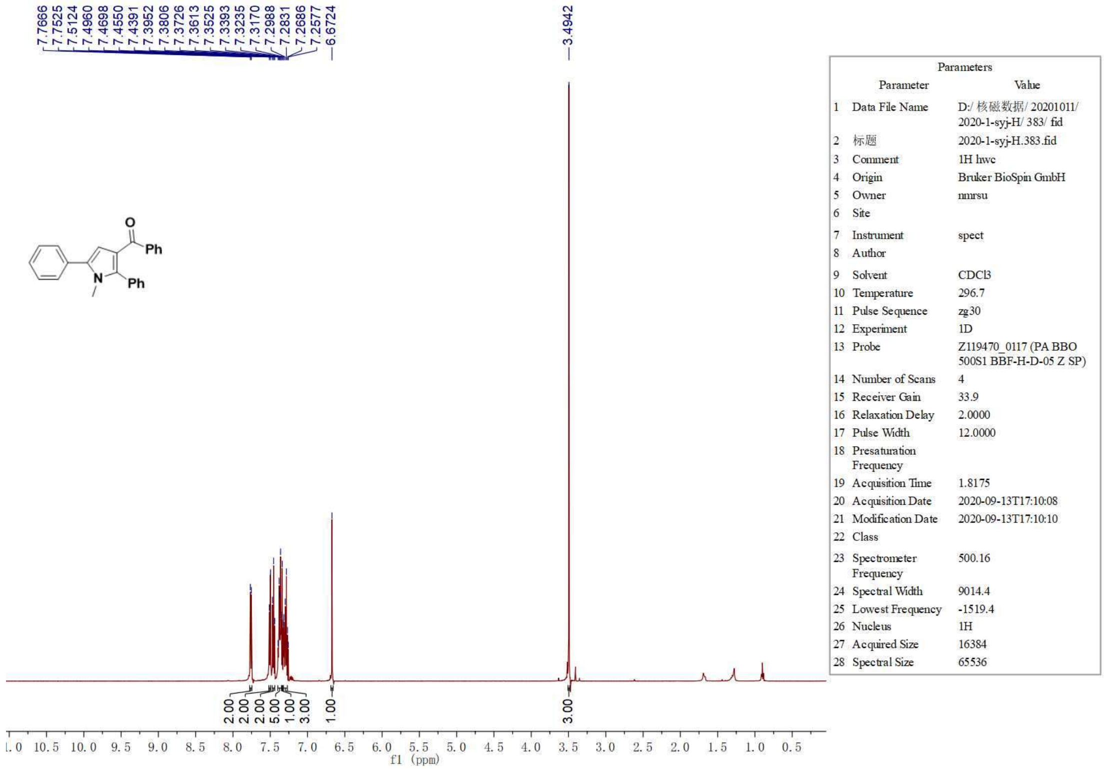

Parameter	Value
1 Data File Name	D:/ 核磁数据/ 20201011/ 2020-1-syj-H/ 383/ fid
2 标题	2020-1-syj-H.383.fid
3 Comment	1H hwc
4 Origin	Bruker BioSpin GmbH
5 Owner	nmrsu
6 Site	
7 Instrument	spect
8 Author	
9 Solvent	CDCl3
10 Temperature	296.7
11 Pulse Sequence	zg30
12 Experiment	1D
13 Probe	Z119470_0117 (PA BBO 500S1 BBF-H-D-05 Z SP)
14 Number of Scans	4
15 Receiver Gain	33.9
16 Relaxation Delay	2.0000
17 Pulse Width	12.0000
18 Presaturation Frequency	
19 Acquisition Time	1.8175
20 Acquisition Date	2020-09-13T17:10:08
21 Modification Date	2020-09-13T17:10:10
22 Class	
23 Spectrometer Frequency	500.16
24 Spectral Width	9014.4
25 Lowest Frequency	-1519.4
26 Nucleus	1H
27 Acquired Size	16384
28 Spectral Size	65536

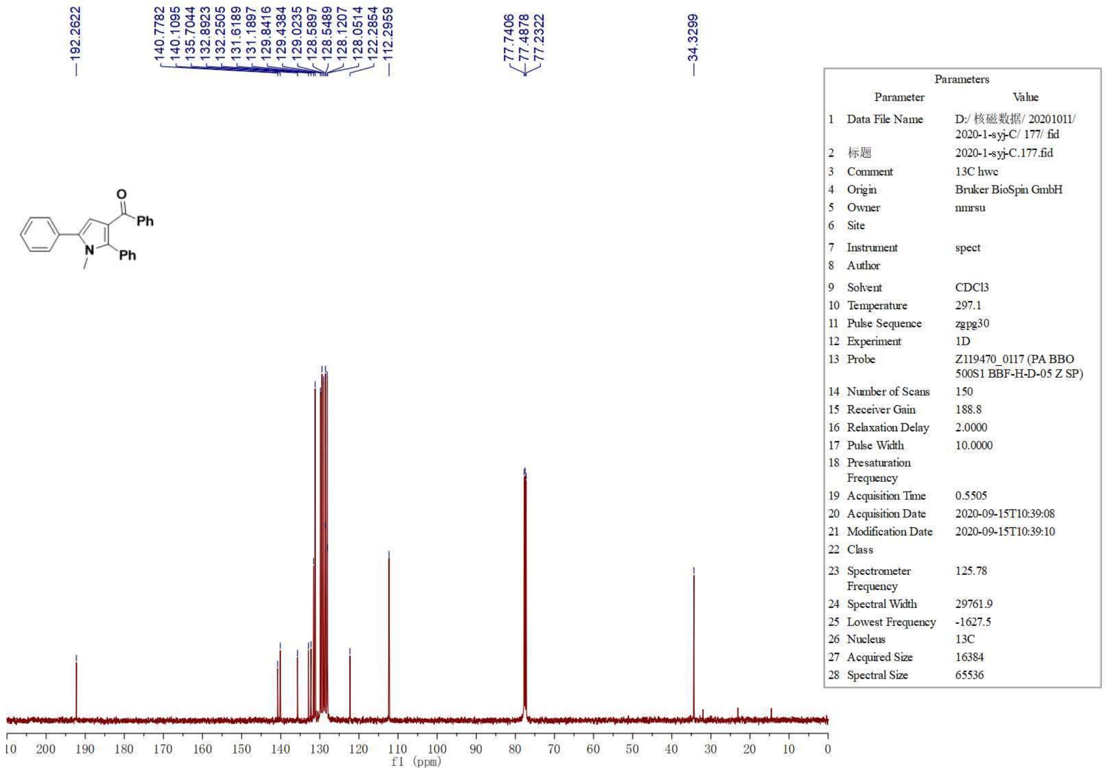

Parameter	Value
1 Data File Name	D:/ 核磁数据/ 20201011/ 2020-1-syj-C/ 177/ fid
2 标题	2020-1-syj-C.177.fid
3 Comment	13C hwc
4 Origin	Bruker BioSpin GmbH
5 Owner	nmrsu
6 Site	
7 Instrument	spect
8 Author	
9 Solvent	CDCl3
10 Temperature	297.1
11 Pulse Sequence	zgpg30
12 Experiment	1D
13 Probe	Z119470_0117 (PA BBO 500S1 BBF-H-D-05 Z SP)
14 Number of Scans	150
15 Receiver Gain	188.8
16 Relaxation Delay	2.0000
17 Pulse Width	10.0000
18 Presaturation Frequency	
19 Acquisition Time	0.5505
20 Acquisition Date	2020-09-15T10:39:08
21 Modification Date	2020-09-15T10:39:10
22 Class	
23 Spectrometer Frequency	125.78
24 Spectral Width	29761.9
25 Lowest Frequency	-1627.5
26 Nucleus	13C
27 Acquired Size	16384
28 Spectral Size	65536

4. GC-MS spectra for mechanistic investigations

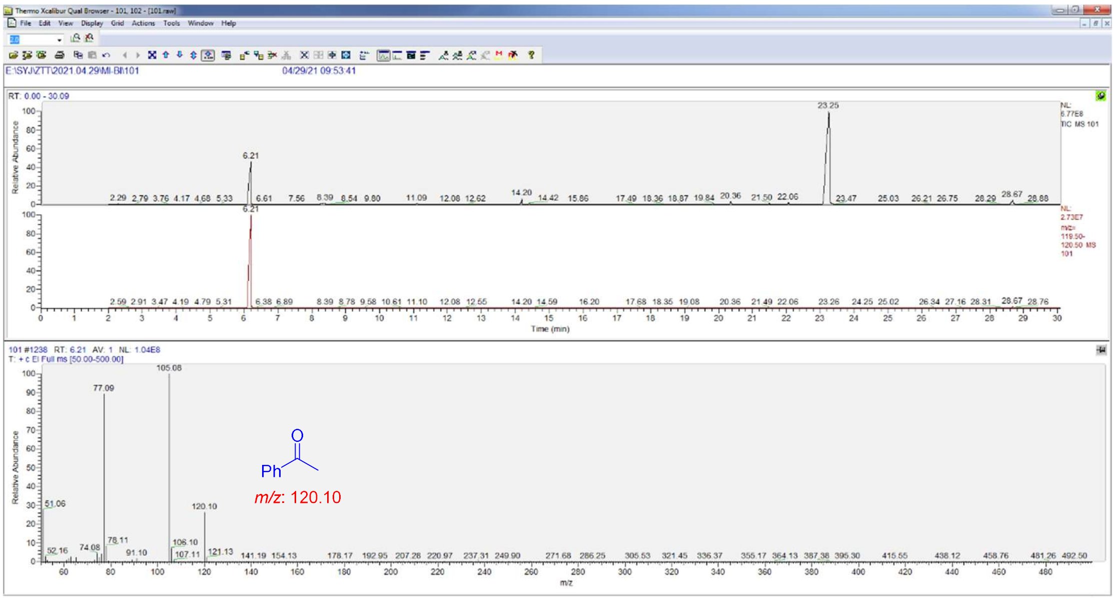

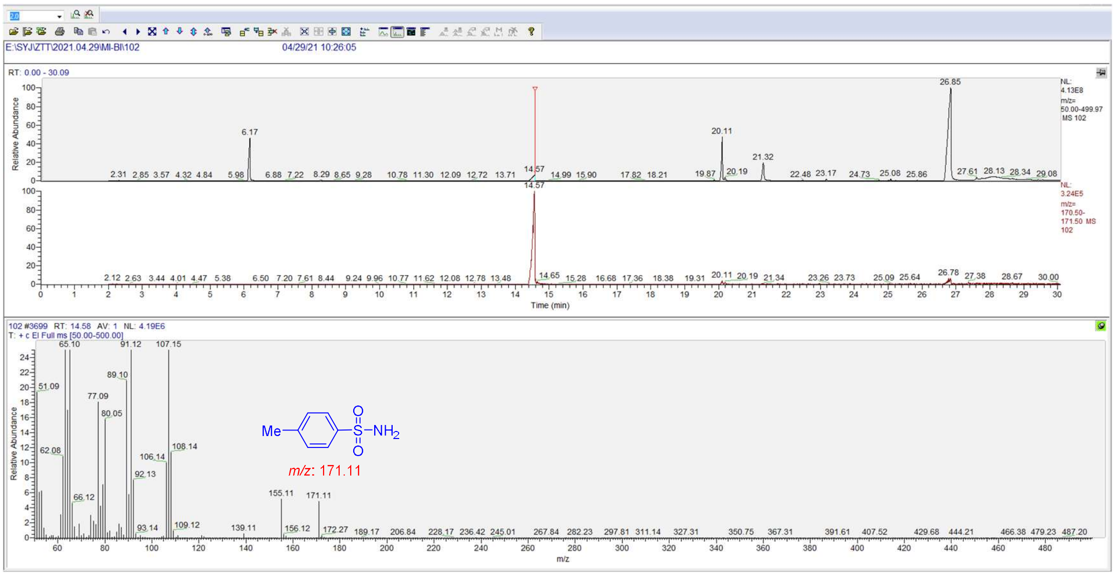

Supplement: Supplementary file 1 [file molecules-28-04416-s001.zip › molecules-2419981-supplementary.pdf]
